# Cyaphide‐Azide 1,3‐Dipolar Cycloaddition Reactions: Scope and Applicability

**DOI:** 10.1002/chem.202301648

**Published:** 2023-08-10

**Authors:** Eric S. Yang, Alex Mapp, Andrew Taylor, Paul D. Beer, Jose M. Goicoechea

**Affiliations:** ^1^ Department of Chemistry University of Oxford Chemistry Research Laboratory 12 Mansfield Rd. Oxford OX1 3TA U.K.; ^2^ Department of Chemistry Indiana University 800 E. Kirkwood Ave. Bloomington IN-47405 USA

**Keywords:** azide, click chemistry, cyaphide, phosphorus, triazaphospholes

## Abstract

Several examples of the cyaphide‐azide 1,3‐dipolar cycloaddition reaction to afford metallo‐triazaphospholes are reported. The gold(I) triazaphospholes Au(IDipp)(CPN_3_R) (IDipp=1,3‐bis(2,6‐diisopropylphenyl)imidazol‐2‐ylidene; R=^*t*^Bu, Ad, Dipp), magnesium(II) triazaphospholes, {Mg(^Dipp^NacNac)(CPN_3_R)}_2_ (^Dipp^NacNac=CH{C(CH_3_)N(Dipp)}_2_, Dipp=2,6‐diisopropylphenyl; R=^*t*^Bu, Bn), and germanium(II) triazaphosphole Ge(^Dipp^NacNac)‐(CPN_3_^*t*^Bu) can be prepared straightforwardly, under mild conditions and in good yields, in a manner reminiscent of the classic alkyne‐azide click reaction (albeit without a catalyst). This reactivity can be extended to compounds with two azide functional groups such as 1,3‐diazidobenzene. It is shown that the resulting metallo‐triazaphospholes can be used as precursors to carbon‐functionalized species, including protio‐ and iodo‐triazaphospholes.

## Graphical abstract

**The use of metal cyaphido compounds** in 1,3‐dipolar cycloaddition reactions with azides to afford metallo‐triazaphospholes is described. The reactions proceed straightforwardly, under mild conditions and in good yields, in a manner reminiscent of the classic alkyne‐azide click reaction. The resulting metallo‐triazaphospholes can be used as precursors to protio‐ and iodo‐triazaphospholes.

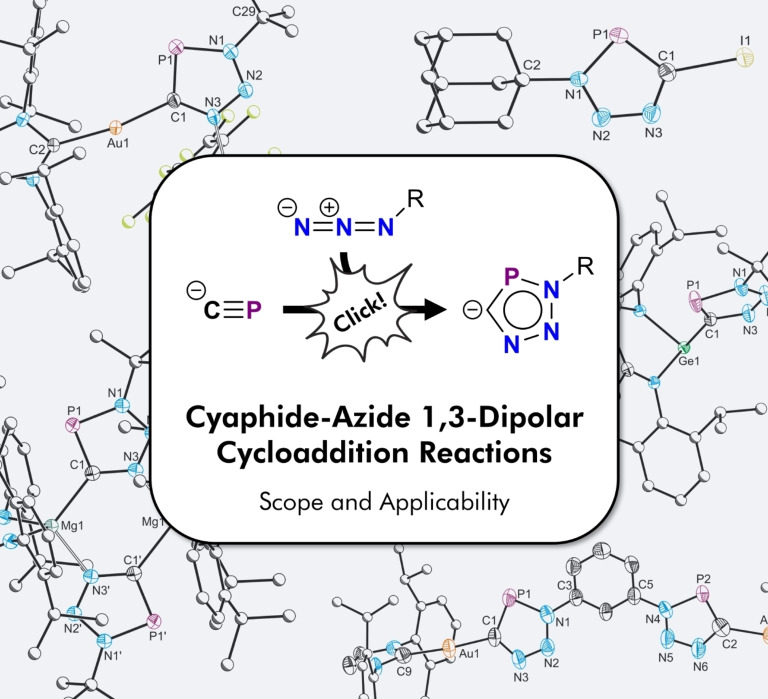

## Introduction

The 1,3‐dipolar cycloaddition reaction between azides and alkynes has become an indispensable reaction in many areas of chemistry.[^1^, ^2^, ^3^, ^4^, ^5^] Variations of this transformation are known as “click” reactions due to their broad applicability, mild reaction conditions, and good selectivity. These include the copper(I)‐catalyzed azide–alkyne “click” reaction (CuAAC),[^6^, ^7^] which has played a pivotal role in the synthesis of mechanically interlocked molecules by active metal templation,[^8^, ^9^] as well as the bio‐orthogonal strain‐promoted azide–alkyne “click” reaction (SPAAC) used in biological labelling.[^10^, ^11^] In inorganic chemistry, 1,3‐dipolar cycloadditions involving metal azides or acetylides provide a useful route to multi‐metallic architectures.^[12]^

Phosphaalkynes (R−C≡P) also undergo 1,3‐dipolar cycloadditions with organic azides to form triazaphospholes, which have garnered attention in recent years as tunable ligand scaffolds and as π‐conjugated luminescent materials.[^13^, ^14^, ^15^] However, the range of accessible triazaphospholes is severely limited by the dearth of kinetically stable phosphaalkynes;^[16]^ only triazaphospholes with carbon‐functionalization by simple alkyl, aryl, or silyl substituents have been demonstrated via this route.^[17]^

**Figure 1:**
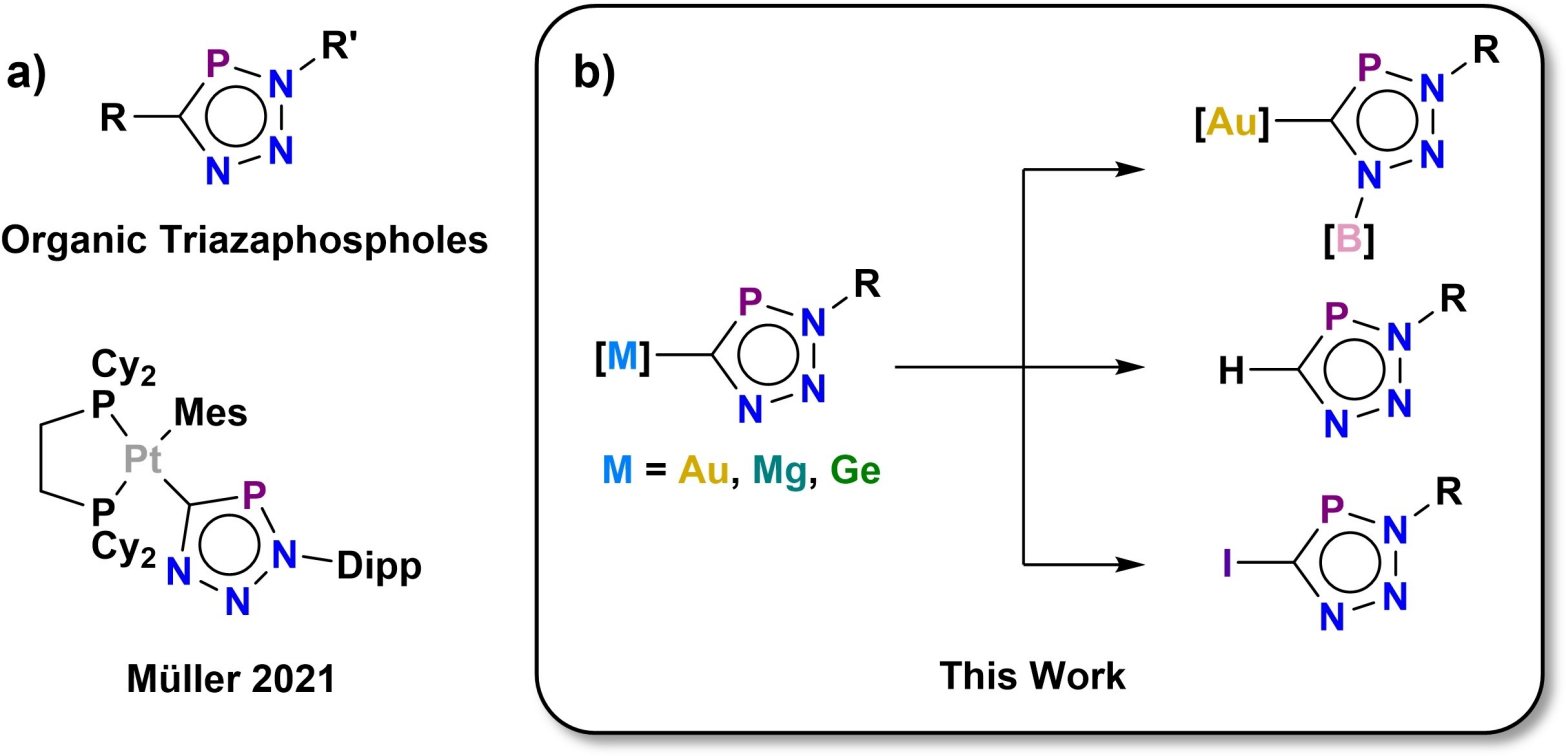
a) Previously reported triazaphospholes; b) Metal triazaphosphole complexes and their reactivity as presented in this work.

The cyaphide ion, C≡P^−^, is a heavy analogue of the cyanide and acetylide ions. To date, there is only one example of a 1,3‐dipolar cycloaddition reaction involving a metal cyaphide complex, reported by Müller, Jones, and co‐workers for a platinum cyaphide complex with N_3_Dipp (Figure 1a), though the reactivity of the resulting triazaphosphole complex was not explored further.^[18]^

The lack of other literature examples is due to the historic inaccessibility of metal cyaphido complexes. Recently, our group has developed a magnesium cyaphide complex that acts as a cyaphide transfer reagent, offering a general route to a variety of metal cyaphido complexes by simple salt metathesis reactions.^[19]^

We reasoned that the highly reactive nature of these metal cyaphide complexes, which are prone to oligomerization in formal [2+2] and [2+2+2] cycloaddition reactions,^[20]^ should allow for facile 1,3‐dipolar cycloadditions with organic azides. We have found that these reactions proceed straightforwardly for a broad range of cyaphide complexes under mild conditions in a manner reminiscent of classic “click” reactions. In this work, we report several examples of the cyaphide‐azide “click” reaction, using cyaphido complexes of s, p, and d block metals to afford metallo‐triazaphospholes (Figure 1b). The reactivity of these metallo‐triazaphospholes to synthesize Lewis acid adducts, as well as protio‐ and iodo‐triazaphospholes is also explored.

## Results and Discussion

**Figure 2:**
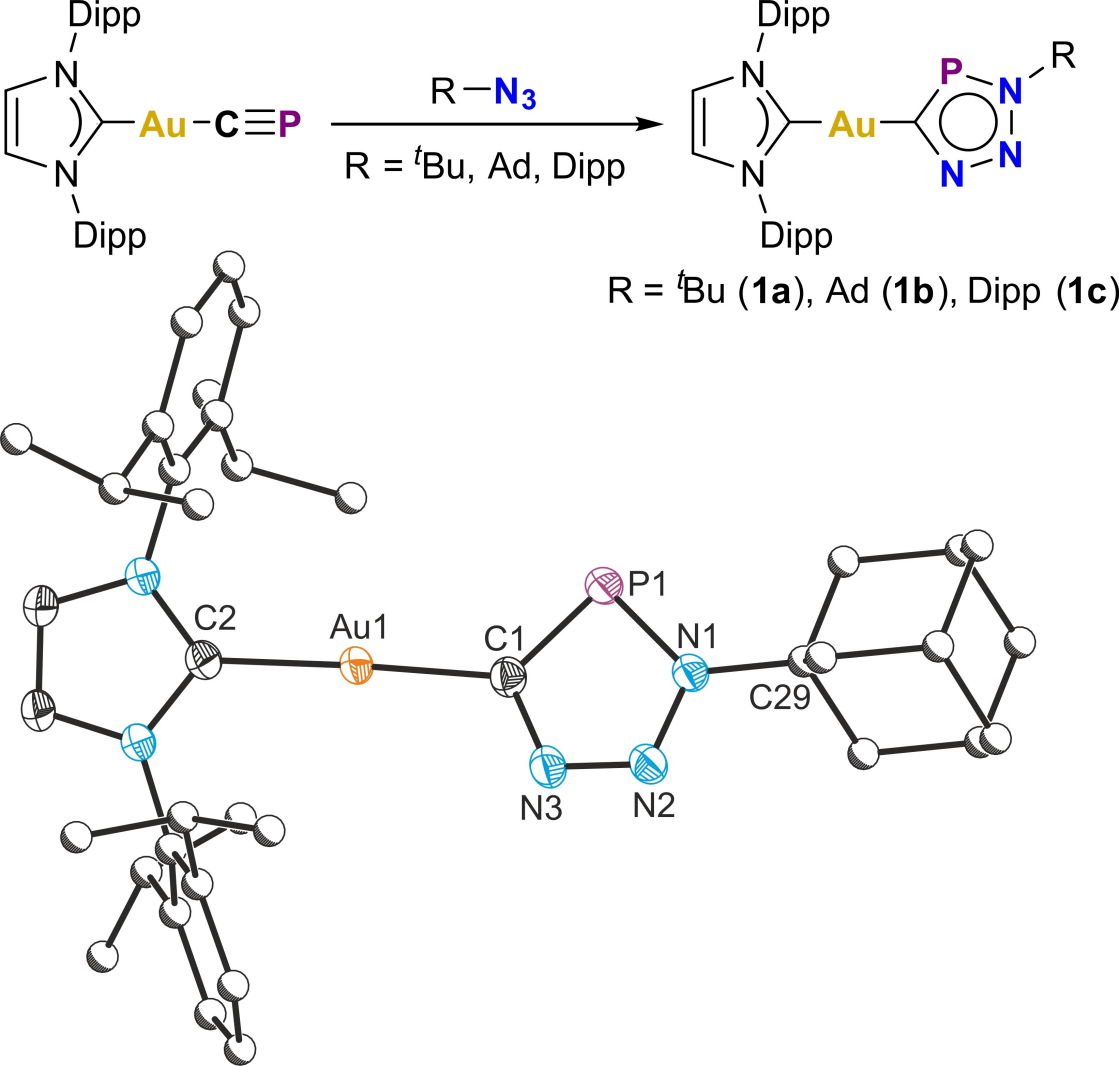
Top: Synthesis of gold(I) triazaphospholes **1a**–**c**. Bottom: Solid‐state structure of **1b**. Anisotropic displacement ellipsoids depicted at 50 % probability. Hydrogen atoms and solvent of crystallization omitted for clarity. Carbon atoms of Dipp and Ad substituents pictured as spheres of arbitrary radius. Selected bond lengths (Å) and angles (°): Au1−C1 2.024(2), Au1−C2 2.028(2), C1−P1 1.715(2), P1−N1 1.701(2), N1−N2 1.339(2), N2−N3 1.311(2), N3−C1 1.371(2), N1−C29 1.487(2), C1−Au1−C2 177.80(7), Au1−C1−P1 127.73(10), C1−P1−N1 88.44(8), P1−N1−N2 114.10(12), N1−N2−N3 112.11(15), N2−N3−C1, 114.39(15), N3−C1−P1, 110.96(13).

We have previously demonstrated that the gold(I) cyaphide complex Au(IDipp)(CP) has phosphaalkyne‐like reactivity toward transition metals,^[21]^ and reasoned that it may also behave as a metallo‐phosphaalkyne in reactions with organic azides. The addition of organic azides to toluene solutions of Au(IDipp)(CP) at room temperature results in the quantitative formation of the gold(I) triazaphosphole complexes Au(IDipp)(CPN_3_R) [R=^*t*^Bu (**1a**), Ad (**1b**), Dipp (**1c**); Figure 2], which can be isolated in very good yields (86–90 %). Complexes **1a**–**c** exhibit ^31^P{^1^H} NMR resonances with chemical shifts similar to organic triazaphospholes (**1a**: 191.2 ppm, **1b**: 189.3 ppm, **1c**: 209.3 ppm).

The solid‐state structures of **1a**–**1c** were determined by single crystal X‐ray crystallography, and reveal linear gold(I) centers κ‐C bonded to the triazaphospholes (**∠**C1−Au1−C2: 179.5(3), 177.80(7) and 177.25(16)° for **1a**–**1c**, respectively). The Au−C bond lengths for the gold‐carbene and gold‐triazaphospholes interactions are statistically identical at approx. 2.02 Å. Similar to organic triazaphospholes,^[17]^ complexes **1a**–**c** are thermally robust and fairly tolerant to air and moisture; in benzene solution, complex **1c** has a half‐life of approx. 4 days in air at room temperature.

**Figure 3:**
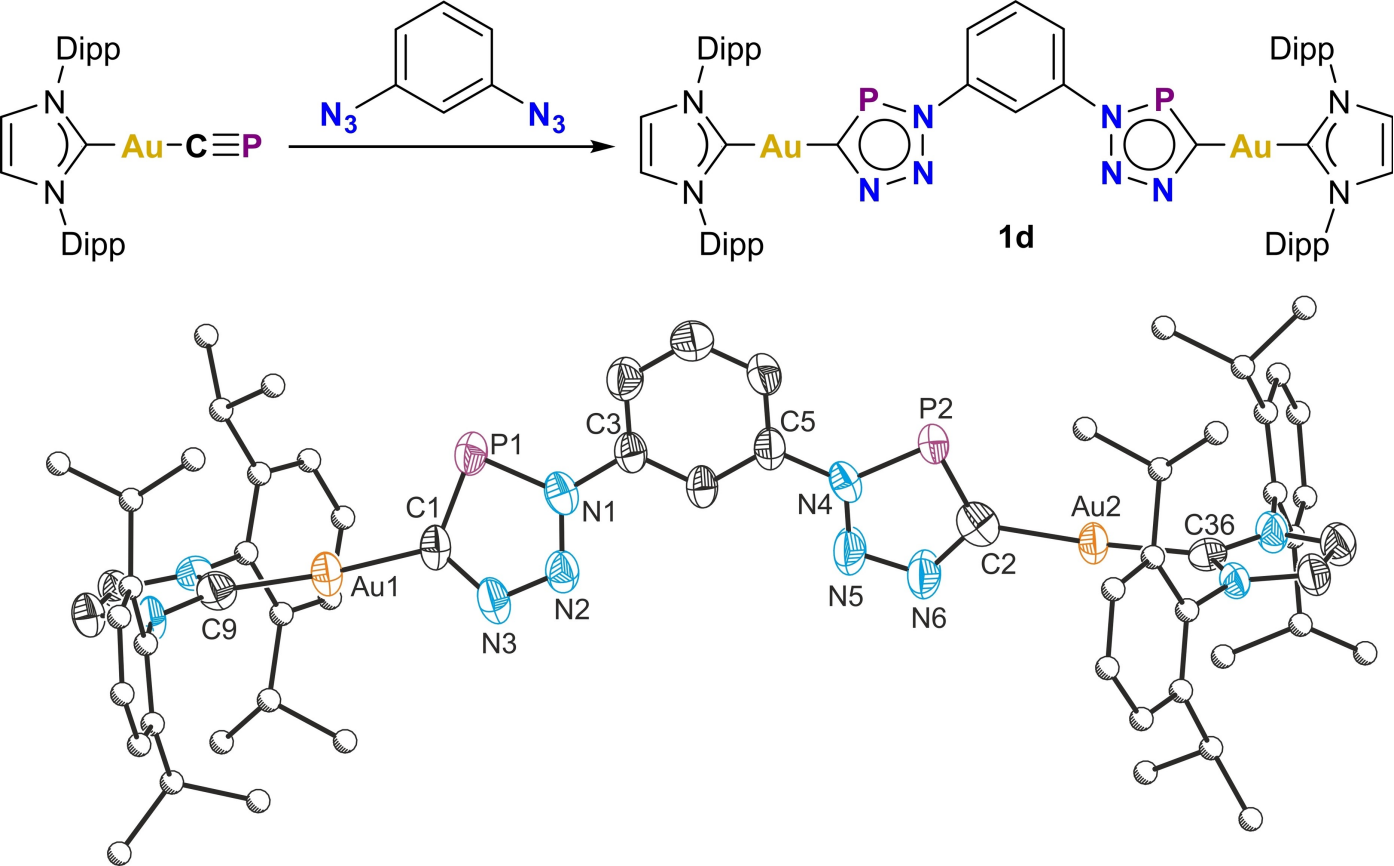
Top: Synthesis of the gold(I) 1,3‐bis(triazaphosphole) complex **1d**. Bottom: Solid‐state structure of **1d**. Anisotropic displacement ellipsoids depicted at 50 % probability. Hydrogen atoms and solvent of crystallization omitted for clarity. Carbon atoms of Dipp substituents pictured as spheres of arbitrary radius. Selected bond lengths (Å) and angles (°): Au1−C1 1.998(8), Au2−C2 2.036(10), C1−P1 1.725(10), P1−N1 1.727(8), N1−N2 1.340(11), N2−N3 1.299(11), N3−C1 1.374(12), N1−C3 1.422(11), C2−P2 1.707(10), P2−N4 1.709(7), N4−N5 1.329(11), N5−N6 1.335(11), N6−C2 1.368(13), N4−C5 1.427(11); C1−Au1−C9 173.9(3), C2−Au2−C36 172.5(4).

Having demonstrated that triazaphospholes are readily accessible using a Au(IDipp)(CP) precursor, we turned our attention to the reactivity of this species towards molecules containing two azide moieties. Molecules containing 1,3‐bis(1,2,3‐triazole) motifs are employed as pincer ligands in organometallic complexes,^[22]^ and as scaffolds for anion sensing applications.[^23^, ^24^] As such, the synthesis of a bimetallic 1,3‐triazaphosphole complex was targeted. The reaction of 1,3‐diazidobenzene with two equivalents of Au(IDipp)(CP) results in the clean formation of the bimetallic gold(I) 1,3‐bis(triazaphosphole)benzene complex [Au(IDipp)(CPN_3_)]_2_(C_6_H_4_) (**1d**; Figure 3). The ^31^P{^1^H} NMR spectrum of **1d** displays a singlet resonance at 200.8 ppm, and its solid‐state structure reveals a formally dianionic 1,3‐bis(triazaphospholide) ligand bridged between two gold(I) centers.

The structure of **1d** was determined by single‐crystal X‐ray diffraction confirming that both of the azide moieties of the diazidobenzene precursor have undergone a 1,3‐dipolar cycloaddition with Au(IDipp)(CP). The asymmetric unit contains two crystallographically unique [Au(IDipp)(CPN_3_)]_2_(C_6_H_4_) molecules, both of which exhibit comparable bond metric parameters. As with the structures of **1a**–**1 c**, the Au−C distances to both the carbene and the triazaphosphole moieties are approx. 2.02 Å, and the C−Au−C bond angles approach 180°.

**Figure 4:**
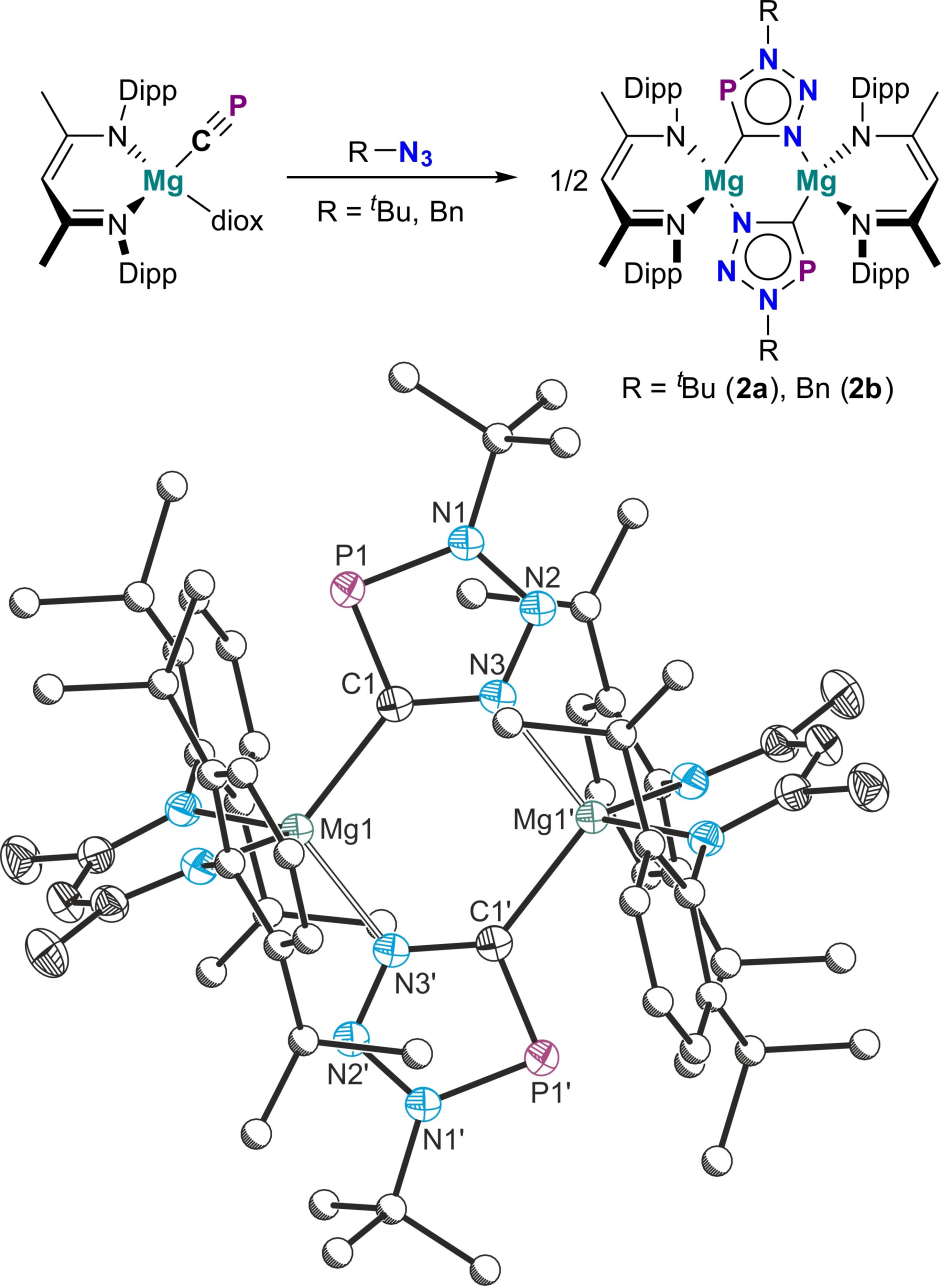
Top: Synthesis of dimeric magnesium(II) triazaphospholes **2a**/**2b**. Bottom: Solid‐state structure of **2a**. Anisotropic displacement ellipsoids depicted at 50 % probability. Hydrogen atoms omitted for clarity. Carbon atoms of Dipp and ^*t*^Bu substituents pictured as spheres of arbitrary radius. Selected bond lengths (Å) and angles (°): Mg1−C1 2.162(2), C1−P1 1.714(2), P1−N1 1.699(2), N1−N2 1.330(2), N2−N3 1.326(2), N3−C1 1.377(2), N3−Mg1′ 2.105(2); Mg1−C1−P1 120.86(8), C1−P1−N1 89.44(7), P1−N1−N2 115.02(10), N1−N2−N3 110.00(12), N2−N3−C1, 116.78(12), N3−C1−P1, 108.77(11), N2−N3−Mg1′ 119.27(10). Symmetry operation ′: ‐*x*, ‐*y*, 1‐*z*.

Compounds **1a**–**d** are spectroscopically and structurally similar to organic triazaphospholes, likely due to the highly covalent nature of Au−C sigma bonds.^[25]^ In order to assess the scope of the cyaphide‐azide cycloaddition reaction, the reactions of organic azides with the highly ionic cyaphide complex Mg(^Dipp^NacNac)(dioxane)(CP) were investigated. The addition of aliphatic azides to freshly prepared toluene solutions of Mg(^Dipp^NacNac)(dioxane)(CP) at room temperature results in the rapid, quantitative formation of the dimeric magnesium(II) triazaphospholes {Mg(^Dipp^NacNac)(CPN_3_R)}_2_ [R=^*t*^Bu (**2a**), Bn (**2b**); Figure 4]. In contrast, the reaction of Mg(^Dipp^NacNac)(dioxane)(CP) with the aromatic azide N_3_Dipp – which successfully reacts with platinum(II) and gold(I) cyaphide complexes – results in an intractable mixture of products. This suggests that the combination of a highly electron‐rich cyaphide in Mg(^Dipp^NacNac)(dioxane)(CP) paired with a relatively electron‐poor aryl azide is sufficient to enable alternative regiochemical outcomes, indicating a potential electronic limitation to the cyaphide‐azide cycloaddition reaction.

On standing, **2a**/**2b** crystallize from toluene solutions at room temperature in good yields. The ^31^P{^1^H} NMR spectrum of **2a** exhibits higher chemical shifts (224.3 ppm) than typical organic triazaphospholes and the gold(I) complexes **1a**–**c**. The poor solubility of **2**  in all the common solvents trialed precluded characterization by NMR spectroscopy, although analysis by single crystal X‐ray diffraction confirms the proposed structure, and elemental analysis supports compositional purity.

The solid‐state structures of **2a**/**2b** reveal dimeric structures in which the triazaphospholes bridge the two magnesium centers by a carbon atom and the adjacent nitrogen atom, in a manner similar to previously reported magnesium *β*‐diketiminato complexes of N‐heterocycles.^[26]^ The Mg1−C1 bond length, 2.162(2) Å, is slightly longer than that observed for Mg(^Dipp^NacNac)(dioxane)(CP) (2.118(2) Å), presumably due to the weaker σ‐donor ability of the triazaphosphole ring and the increased steric bulk around the magnesium(II) center. On cyclization the C−P bond length increases significantly from 1.553(2) to 1.714(2) Å. The dimeric structure of **2a** is retained in solution as evidenced by the presence of upfield‐shifted isopropyl resonances due to neighboring aryl diatropic ring currents (contrasting with comparable monomeric species; see below). ^1^H DOSY experiments further support a dimeric structure in solution. Notably, the structure of **2a** persists even in donor solvents (e.g. THF, MeCN, pyridine) or in the presence of strong Lewis bases (e.g. DMAP) or strong Lewis acids (e.g. B(C_6_F_5_)_3_).

**Figure 5:**
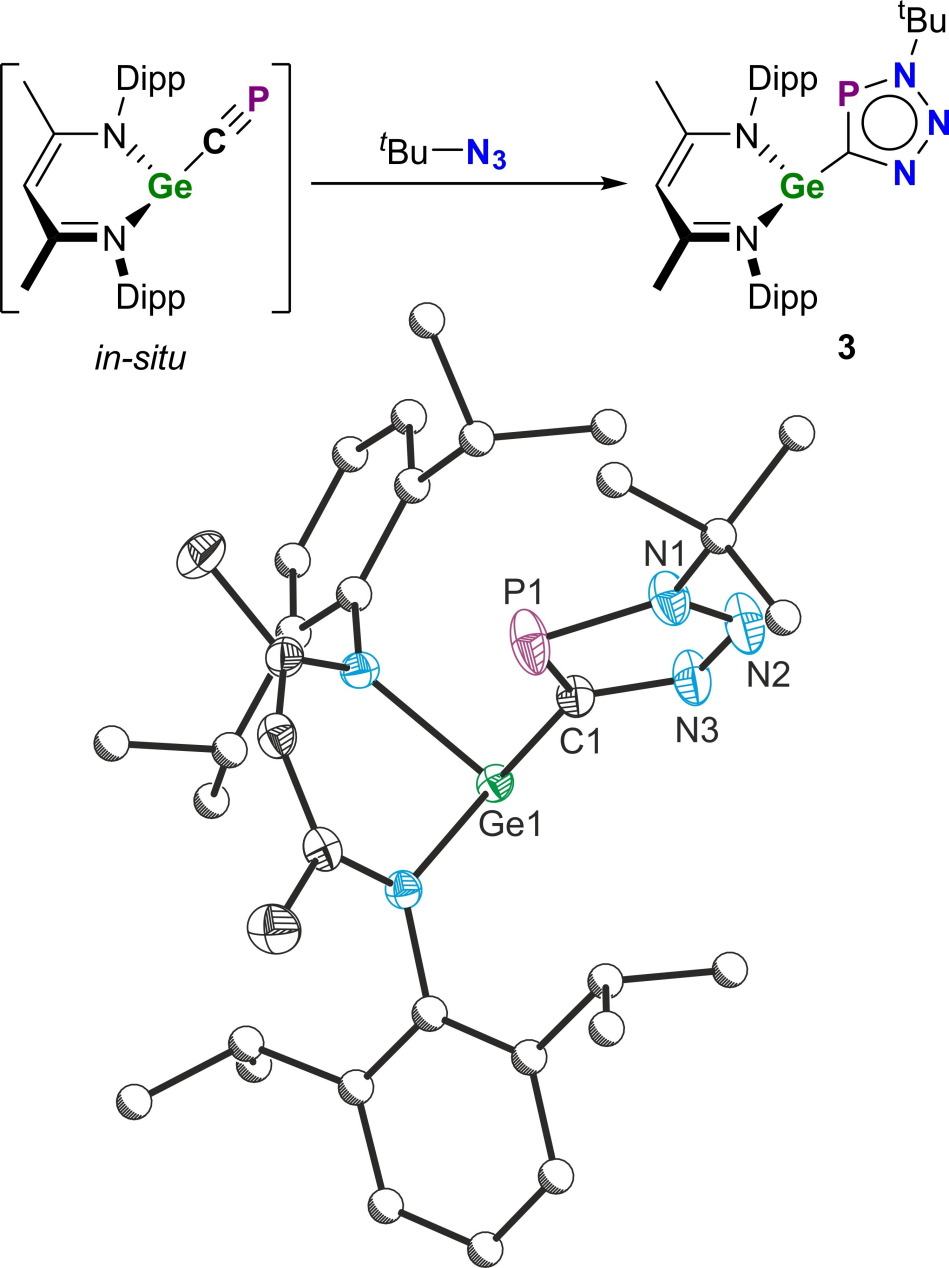
Top: Synthesis of the germanium(II) triazaphosphole **3**. Bottom: Solid‐state structure of **3**. Anisotropic displacement ellipsoids depicted at 50 % probability. Hydrogen atoms omitted for clarity. Carbon atoms of Dipp and ^*t*^Bu substituents pictured as spheres of arbitrary radius. Selected bond lengths (Å) and angles (°): Ge1−C1 2.038(2), C1−P1 1.717(2), P1−N1 1.691(2), N1−N2 1.335(2), N2−N3 1.312(2), N3−C1 1.357(2); Ge1−C1−P1 134.43(8), C1−P1−N1 88.00(6), P1−N1−N2 114.75(10), N1−N2−N3 111.49(12), N2−N3−C1, 114.66(12), N3−C1−P1, 111.10(10).

The mild conditions and relatively quick reaction times of the cyaphide‐azide cycloaddition reaction to form **1a**–**c** and **2a**/**2b** prompted us to consider whether this could be used to trap highly reactive cyaphide‐containing molecules. We previously reported a germanium(II) cyaphide complex Ge(^Dipp^NacNac)(CP) which was kinetically unstable and not isolable.^[19]^ Nevertheless, the addition of *tert*‐butyl azide to a freshly prepared toluene solution of Ge(^Dipp^NacNac)(CP) resulted in the rapid formation of the germanium(II) triazaphosphole complex Ge(^Dipp^NacNac)‐(CPN_3_^*t*^Bu) (**3**; Figure 5). **3** exhibits a chemical shift at 196.9 ppm in its ^31^P{^1^H} NMR spectrum.

The solid‐state structure of **3** was determined by single crystal X‐ray diffraction. The bond metric parameters of the triazaphosphole ring are similar to those observed for **2a**/**2b**. The Ge1−C1 bond length is 2.038(2) Å, which is comparable to that of other three‐coordinate *β*‐diketiminato‐stabilized germanium(II) complexes such as Ge(^Dipp^NacNac)(2‐thienyl) (1.966(9) Å).^[27]^ In contrast to the unstable, highly sensitive cyaphido precursor, **3** is air‐ and moisture‐tolerant and can be handled in air for short periods of time.

**Figure 6:**
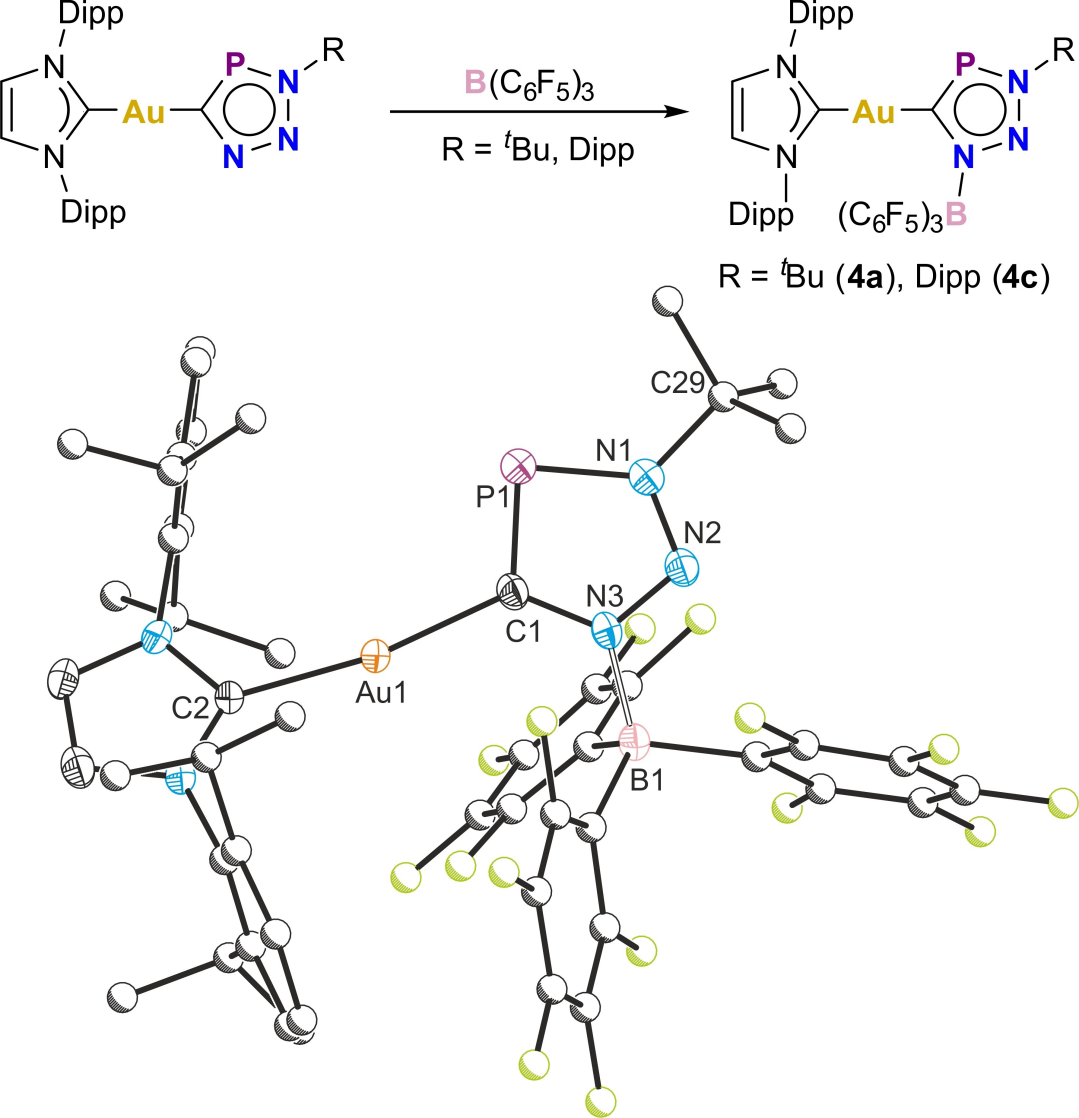
Top: Coordination of **1a**/**1c** to B(C_6_F_5_)_3_ to afford the Lewis adducts **4a**/**4c**. Bottom: Solid‐state structure of **4a**. Anisotropic displacement ellipsoids depicted at 50 % probability. Hydrogen atoms omitted for clarity. Carbon atoms of Dipp, ^*t*^Bu and C_6_F_5_ substituents pictured as spheres of arbitrary radius. Selected bond lengths (Å) and angles (°): Au1−C1 2.028(4), Au1−C2 2.028(4), C1−P1 1.706(5), P1−N1 1.715(4), N1−N2 1.315(6), N2−N3 1.328(5), N3−C1 1.361(6), N1−C29 1.501(6), N3−B1 1.606(6); C1−Au1−C2 170.35(19), Au1−C1−P1 118.2(2), C1−P1−N1 88.2(2), P1−N1−N2 115.5(3), N1−N2−N3 109.7(4), N2−N3−C1 116.6(4), N3−C1−P1 110.0(3), N2−N3−B1 117.5(3).

The triazaphosphole ligands in complexes **1**–**3** are donor rich, with four *exo* lone pairs. The dimeric structure of the magnesium(II) complexes **2a**/**b** with trisubstituted triazaphosphole ligands suggest that the disubstituted gold(I) triazaphospholes **1a**–**c** should be capable of further coordination to Lewis acids. Indeed, the reaction of **1a** or **1c** with B(C_6_F_5_)_3_ results in the trisubstituted triazaphosphole complexes **4a**/**4c** (Figure 6). Compounds **4a**/**4c** exhibit ^31^P{^1^H} NMR chemical shifts at 203.5 and 220.4 ppm, respectively, slightly downfield shifted compared to the precursors. **4a** and **4c** each have one broad resonance in their ^11^B{^1^H} NMR spectra at −5.52 and −5.02 ppm, respectively (typical of B(C_6_F_5_)_3_ Lewis adducts), and complex ^19^F{^1^H} NMR spectra due to restricted rotation of the B(C_6_F_5_)_3_ groups.

The solid‐state structures of **4a** and **4c** were determined by single‐crystal X‐ray diffraction, revealing trisubstituted triazaphosphole ligands coordinated to the Au(IDipp) moiety via their carbon atoms, and to the B(C_6_F_5_)_3_ fragment via the adjacent nitrogen atoms. The bond metrics of the compounds are largely identical with those of **1a**/**1c**, with comparable Au−C bond distances to both substituents. **4a** and **4c** are isolobal to a phosphorus‐containing carbene analogue previously reported by Müller and co‐workers,^[28]^ and can be considered as gold(I) complexes of anionic phosphorus‐containing abnormal carbenes.

**Scheme 1:**
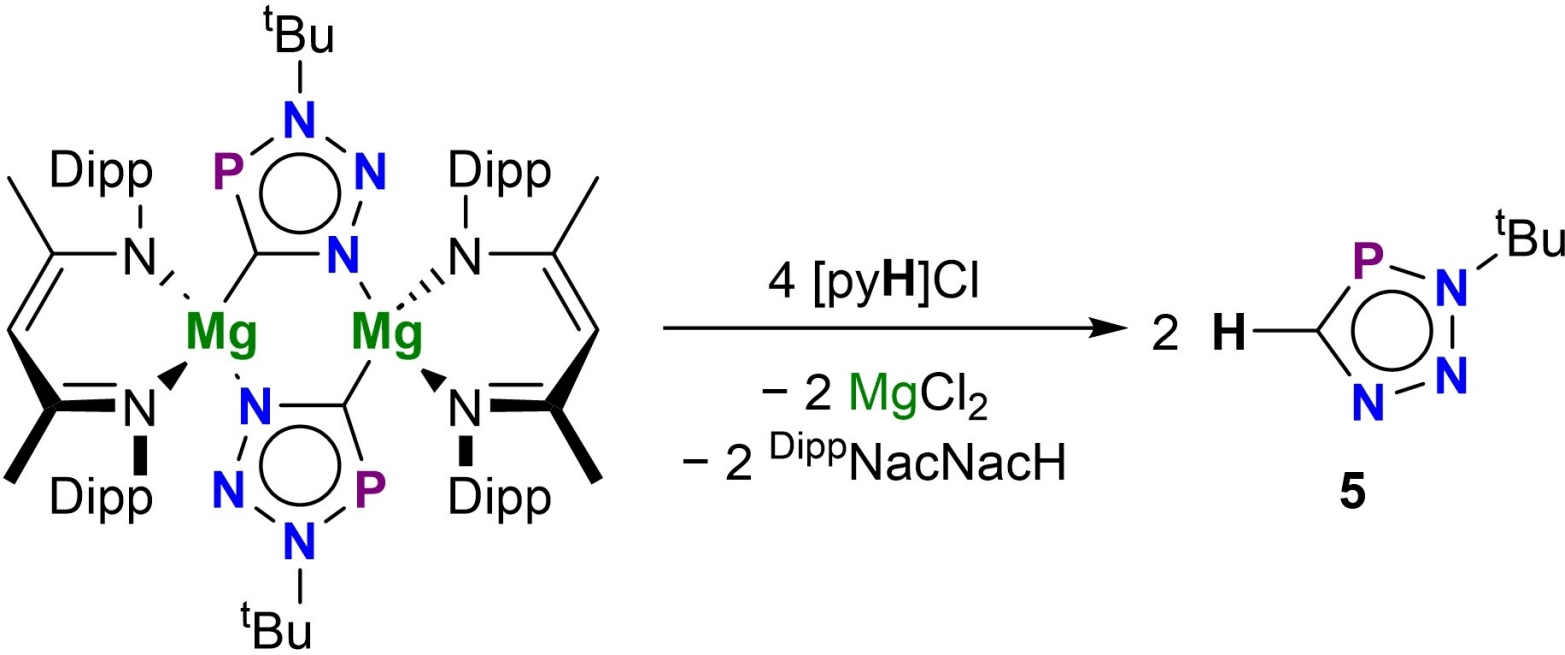
Protonation of **2a** to afford parent *tert*‐butyl triazaphosphole **5**.

The polar M−C bonds in these metallo‐triazaphospholes should allow them to act as precursors to carbon‐functionalized triazaphospholes. To date, only two carbon‐protonated triazaphospholes have been reported, synthesized by the reaction of azides with HCP gas.[^29^, ^30^] As such, the protonation of the magnesium(II) triazaphosphole **2a** was investigated. The reaction of **2a** with four equivalents of pyridinium chloride results in the concomitant formation of MgCl_2_, ^Dipp^NacNacH, and volatile *tert*‐butyl triazaphosphole HCPN_3_^*t*^Bu (**5**; Scheme 1), which could be separated from the reaction mixture by vacuum distillation. **5** has a ^31^P{^1^H} spectrum with a single resonance at 174.9 ppm, and a ^1^H NMR spectrum with a doublet at 8.83 ppm (^2^ *J*_H‐P_=54.7 Hz).

**Figure 7:**
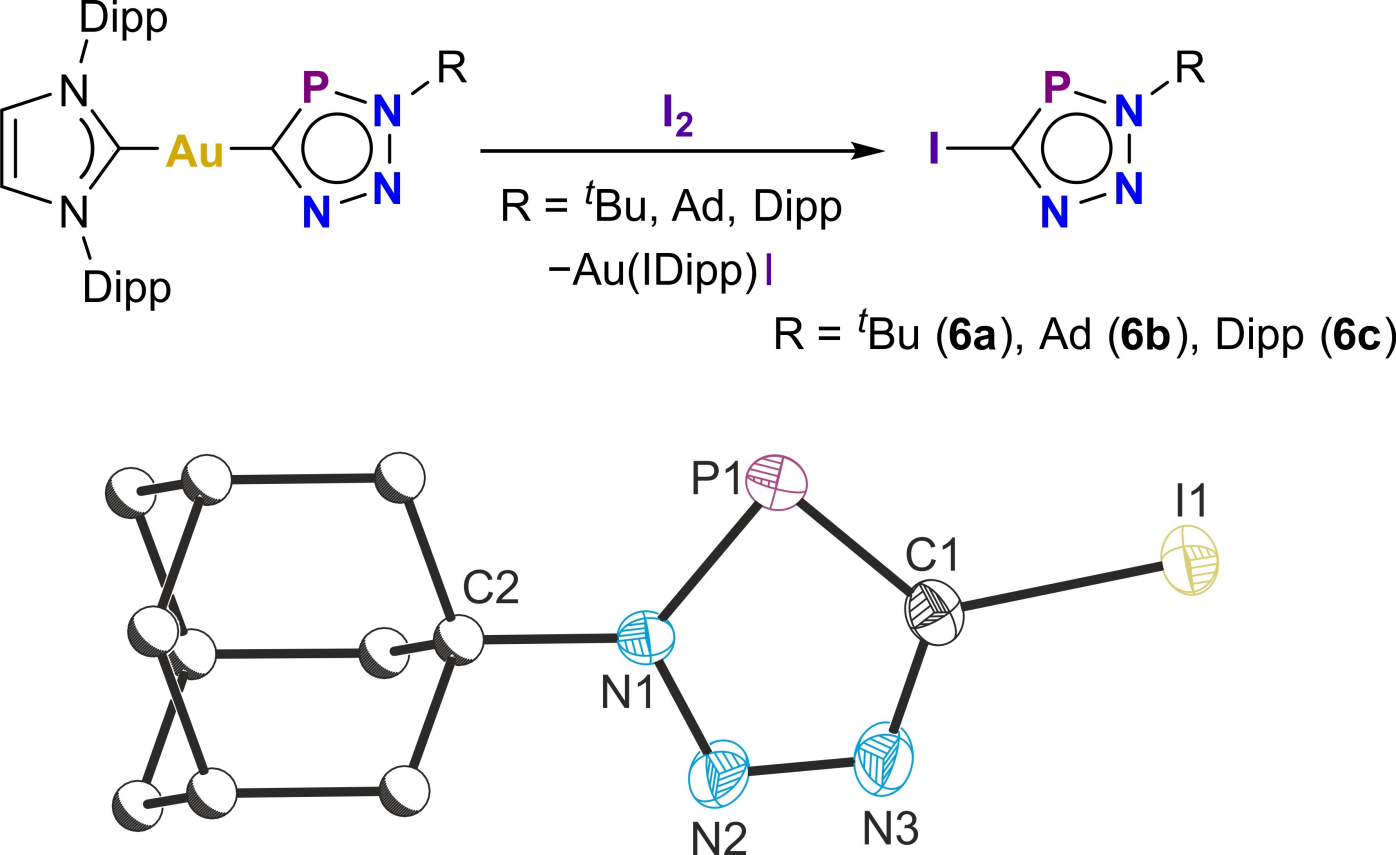
Top: Synthesis of iodotriazaphospholes **6a**–**c**. Bottom: Solid‐state structure of **6 b**. Anisotropic displacement ellipsoids depicted at 50 % probability. Hydrogen atoms omitted for clarity. Carbon atoms of Ad substituent pictured as spheres of arbitrary radius. Selected bond lengths (Å) and angles (°): I1−C1 2.080(3), C1−P1 1.716(3), P1−N1 1.693(3), N1−N2 1.340(3), N2−N3 1.316(4), N3−C1 1.337(4), N1−C2 1.495(4); I1−C1−P1 125.23(17), C1−P1−N1 85.22(13), P1−N1−N2 116.2(2), N1−N2−N3 111.4(3), N2−N3−C1 112.0(3), N3−C1−P1 115.1(2).

Halogenation of the gold(I) triazaphosphole complexes **1a**–**c** was also investigated. The reactions of **1a**–**c** with iodine result in the formation of the iodotriazaphospholes ICPN_3_R [R=^*t*^Bu (**6a**), Ad (**6b**), Dipp (**6c**); Figure 7], which are, to our knowledge, the first reported examples of halogenated triazaphospholes. These exhibit ^31^P{^1^H} spectra with resonances at 179.9, 180.1, and 194.7 ppm for **6a**–**6c**, and ^13^C{^1^H} NMR spectra with doublets at 120.80 ppm (**6a**, ^1^ *J*_C‐P_=80.6 Hz), 120.50 ppm (**6b**, ^1^ *J*_C‐P_=80.6 Hz), and 123.36 ppm (**6c**, ^1^ *J*_C‐P_=83.5 Hz).

The solid‐state structures of **6a** and **6b** could be determined by single‐crystal X‐ray diffraction and were found to exhibit bond metrics in line with all the previously discussed structures. The structure of compound 6a reveals close I⋅⋅⋅I contacts between neighboring molecules (5.63 Å), whereas the solid‐state structure of **6b** shows close contacts between neighboring triazaphospholes corresponding to π‐π interactions.

The ^31^P{^1^H} NMR signals of the *tert*‐butyl substituted triazaphospholes **5** and **6a** appear as pseudo‐triplets in solution, whereas the ^31^P{^1^H} NMR signal of the bulkier Dipp substituted triazaphosphole **6c** appears as a singlet. Based on the presence of multiple intermolecular non‐covalent interactions in the solid‐state, we propose that the appearance of the ^31^P{^1^H} NMR spectra of **5**–**6** can be explained by π‐π aggregation in solution. DFT calculations suggest aggregation is significantly thermodynamically favorable, with a π–π stacking energy of −11.1 kcal mol^−1^. This is further corroborated by the ^31^P{^1^H} NMR spectra of the adamantyl substituted triazaphosphole **6b** in solvents with differing π‐stacking abilities. In hexane, the ^31^P{^1^H} NMR resonance appears as a relatively sharp pseudo‐triplet, which collapses to broader multiplets in benzene and 1,2‐difluorobenzene. However, a convection compensated ^1^H DOSY NMR experiment of **6b** shows a hydrodynamic diameter in good agreement with the monomeric iodotriazaphosphole, though this could be due to rapid chemical exchange between aggregate units faster than the DOSY NMR timescale.^[31]^

## Conclusions

The cyaphide‐azide cycloaddition reaction shows many hallmarks of classic “click” reactions; the reactions proceed at room temperature with regiospecificity and are applicable to a broad range of metal cyaphido complexes, with examples for s, p, and d block metals. Moreover, the reactions proceed in the absence of a catalyst, which may prove their significance for applications in which the presence of extraneous metal ions is deleterious. The resulting metallo‐triazaphospholes react further with Lewis acids to afford trisubstituted triazaphospholes, and can also be cleaved from their coordinated metal centers in protonation or halogenation reactions to afford simple protio‐ and iodo‐ triazaphospholes. The cyaphide‐azide cycloaddition reaction will undoubtedly be useful for preparing functional triazaphospholes, for example in inorganic materials or mechanically interlocked molecules.

## Experimental Section

**General experimental methods**: All reactions and product manipulations were carried out using standard Schlenk‐line techniques under an inert atmosphere of argon, or in a dinitrogen filled glovebox (MBraun UNIlab glovebox maintained at <0.1 ppm H_2_O and <0.1 ppm O_2_). *Tert*‐butyl azide,^[32]^ 1,3‐diisopropylphenyl azide,^[33]^ 1,3‐diazidobenzene,^[34]^ Au(IDipp)(CP),^[19]^ and Ge(^Dipp^NacNac)Cl^[35]^ were synthesized according to previously reported synthetic procedures. Mg(^Dipp^NacNac)(diox.)(CP) was generated *in situ* according to a previously reported procedure.^[19]^ Adamantyl azide (Sigma Aldrich, 97 %), pyridinium chloride (Sigma Aldrich, 98 %) and iodine (Sigma Aldrich, 99.8 %) were purchased and used as received. Benzyl azide (Alfa Aesar, 94 %) was distilled and dried over 3 Å molecular sieves. Toluene (Sigma Aldrich, HPLC grade), hexane (Sigma Aldrich, HPLC grade), and pentane (Sigma Aldrich, HPLC grade) were purified using an MBraun SPS‐800 solvent system. THF (Sigma Aldrich, HPLC grade) was distilled over sodium/benzophenone. 1,2‐DFB (Alfa Aesar, 98 %) was distilled over CaH_2_. C_6_D_6_ (Aldrich, 99.5 %) and CD_2_Cl_2_ (Aldrich, 99.5 %) were degassed and dried over CaH_2_. d_8_‐toluene (Aldrich, 99.5 %) was degassed and dried over Na/K alloy. All dry solvents were stored under argon in gas‐tight ampoules over activated 3 Å molecular sieves.

NMR spectra were acquired on a Bruker AVIII 400 MHz NMR spectrometer (^1^H 400 MHz, ^31^P 162 MHz, ^19^F 377 MHz, ^11^B 128 MHz), Bruker AVIII 500 MHz NMR spectrometer (^1^H 500 MHz, ^13^C 126 MHz) or a Bruker Avance NEO 600 MHz NMR spectrometer with a broadband helium cryoprobe (^1^H 600 MHz, ^13^C 151 MHz). ^1^H and ^13^C NMR spectra were referenced to the most downfield solvent resonance (^1^H NMR C_6_D_6_: δ=7.16 ppm, ^13^C NMR C_6_D_6_: δ=128.06 ppm; ^1^H NMR CD_2_Cl_2_: δ=5.32 ppm, ^13^C NMR CD_2_Cl_2_: δ=53.84 ppm). ^31^P NMR spectra were externally referenced to an 85 % solution of H_3_PO_4_ in H_2_O. ^11^B NMR spectra were externally referenced to BF_3_ ⋅ Et_2_O in C_6_D_6_. ^19^F NMR spectra were externally referenced to CFCl_3_. Elemental analyses were carried out by Elemental Microanalyses Ltd. (Devon, U.K.) or by London Metropolitan University (London, U.K.). Samples (approx. 5 mg) were submitted in flame sealed glass tubes.

### General experimental methods

*Synthesis of Au(IDipp)(CPN_3_^t^Bu) (**1a**)*: Neat *tert*‐butyl azide (8 mg, 0.08 mmol) was added to a solution of Au(IDipp)(CP) (50 mg, 0.08 mmol) in toluene (1 mL) and stirred at room temperature for 2 h. The solution was concentrated by slow evaporation, yielding colorless crystals suitable for single crystal X‐ray crystallography. The mixture was then stored at −35 °C for 3 days to yield colorless crystals which were isolated by filtration. Yield: 50 mg, 0.07 mmol, 86 %. Anal. Calcd. (%) for C_32_H_45_AuN_5_P: C, 52.82; H, 6.23; N, 9.62. Found: C, 52.49; H, 6.31; N, 9.00. ^1^H NMR (600 MHz, C_6_D_6_): δ(ppm) 7.18 (t, ^3^ *J*_H‐H_=7.8 Hz, 2H, Dipp *para*‐C*H*), 7.06 (d, ^3^ *J*_H‐H_=7.8 Hz, 4H, Dipp *meta*‐C*H*), 6.35 (s, 2H, IDipp C*H*), 2.69 (sept, ^3^ *J*_H‐H_=6.9 Hz, 4H, Dipp C*H*(CH_3_)_2_), 1.53 (d, ^3^ *J*_H‐H_=6.9 Hz, 12H, Dipp CH(C*H*_3_)_2_), 1.42 (s, 9H, ^*t*^Bu C(C*H*_3_)_3_), 1.10 (d, ^3^ *J*_H‐H_=6.9 Hz, 12H, Dipp CH(C*H*_3_)_2_). ^13^C{^1^H} NMR (151 MHz, C_6_D_6_): δ(ppm) 205.19 (d, ^1^ *J*_C‐P_=82.0 Hz, *C*PN_3_^*t*^Bu), 196.51 (d, ^3^ *J*_C‐P_=11.1 Hz, IDipp {HCN(Dipp)}_2_ *C*Au), 145.90 (Dipp *ortho*‐*C*), 134.80 (Dipp *ipso*‐*C*), 130.68 (Dipp *para*‐*C*), 124.26 (Dipp *meta*‐*C*), 122.73 (IDipp *C*H), 59.69 (d, ^2^ *J*_C‐P_=5.1 Hz, ^*t*^Bu *C*(CH_3_)_3_), 32.03 (d, ^3^ *J*_C‐P_=5.0 Hz, ^*t*^Bu C(*C*H_3_)_3_), 29.14 (Dipp *C*H(CH_3_)_2_), 24.93 (Dipp CH(*C*H_3_)_2_), 23.98 (Dipp *C*H(CH_3_)_2_). ^31^P{^1^H} NMR (162 MHz, C_6_D_6_): δ(ppm) 191.2 (s, C*P*N_3_^*t*^Bu).

*Synthesis of Au(IDipp)(CPN_3_Ad) (**1b**)*: 1‐adamantyl azide (20 mg, 0.11 mmol) was added to a solution of Au(IDipp)(CP) (71 mg, 0.11 mmol) in toluene (1 mL) and stirred at room temperature overnight. The solution was concentrated under vacuum, then stored at −35 °C for 3 days to yield colorless crystals which were isolated by filtration. Yield: 82 mg, 0.10 mmol, 90 %. Anal. Calcd. (%) for C_38_H_51_AuN_5_P: C, 56.64; H, 6.38; N, 8.69. Found: C, 57.05; H, 6.17; N, 8.43. ^1^H NMR (600 MHz, C_6_D_6_): δ(ppm) 7.20 (t, ^3^ *J*_H‐H_=7.8 Hz, 2H, Dipp *para*‐C*H*), 7.07 (d, ^3^ *J*_H‐H_=7.8 Hz, 4H, Dipp *meta*‐C*H*), 6.39 (s, 2H, IDipp C*H*), 2.70 (sept, ^3^ *J*_H‐H_=6.9 Hz, 4H, Dipp C*H*(CH_3_)_2_), 2.17 (d, ^3^ *J*_H‐H_=2.8 Hz, 6H, Ad C{C*H*_2_CHCH_2_}_3_), 1.85–1.77 (m, 3H, Ad {CH_2_C*H*CH_2_}_3_), 1.54 (d, ^3^ *J*_H‐H_=6.9 Hz, 12H, Dipp CH(C*H*_3_)_2_), 1.45–1.37 (m, 6H, Ad {CH_2_CHC*H*_2_}_3_), 1.11 (d, ^3^ *J*_H‐H_=6.9 Hz, 12H, Dipp CH(C*H*_3_)_2_). ^13^C{^1^H} NMR (151 MHz, C_6_D_6_): δ(ppm) 204.52 (d, ^1^ *J*_C‐P_=81.9 Hz, *C*PN_3_Ad), 196.54 (d, ^3^ *J*_C‐P_=11.0 Hz, IDipp {HCN(Dipp)}_2_ *C*Au), 145.89 (Dipp *ortho*‐C), 134.82 (Dipp *ipso*‐C), 130.70 (Dipp *para*‐C), 124.27 (Dipp *meta*‐C), 122.78 (IDipp C*H*), 60.19 (d, ^2^ *J*_C‐P_=4.4 Hz, Ad *C*{CH_2_CHCH_2_}_3_), 45.76 (d, ^3^ *J*_C‐P_=5.1 Hz, Ad C{*C*H_2_CHCH_2_}_3_), 36.54 (Ad C{CH_2_CH*C*H_2_}_3_), 30.28 (Ad C{CH_2_ *C*HCH_2_}_3_), 29.15 (Dipp *C*H(CH_3_)_2_), 24.94 (Dipp CH(*C*H_3_)_2_), 24.01 (Dipp CH(*C*H_3_)_2_). ^31^P{^1^H} NMR (162 MHz, C_6_D_6_): δ(ppm) 189.3 ppm (s, C*P*N_3_Ad).

*Synthesis of Au(IDipp)(CPN_3_Dipp) (**1c**)*: Neat 2,6‐diisopropylphenyl azide (17 mg, 0.08 mmol) was added to a solution of Au(IDipp)(CP) (50 mg, 0.08 mmol) in toluene (1 mL) and stirred at room temperature overnight. Crystals suitable for X‐ray crystallography were obtained by concentrating the toluene solution followed by layering with hexane (5 mL). The supernatant solution was removed, and the crystals washed with pentane (2×2 mL), then dried under vacuum. Yield: 54 mg, 0.06 mmol, 81 %. Anal. Calcd. (%) for C_40_H_54_N_5_AuP ⋅ 0.5(C_7_H_8_): C, 61.03; H, 6.76; N, 7.57. Found: C, 60.90; H, 6.49; N, 7.24. ^1^H NMR (600 MHz, C_6_D_6_): δ(ppm) 7.12–7.09 (m, 2H, Dipp Ar*H*), 7.02–7.00 (m, 7H, Dipp Ar*H*), 6.32 (s, 2H, IDipp C*H*), 2.68 (sept, ^3^ *J*_H‐H_=6.9 Hz, 4H, Dipp C*H*(CH_3_)_2_, 2.15 (sept, ^3^ *J*_H‐H_=6.8 Hz, 2H, Dipp C*H*(CH_3_)_2_, 1.54 (d, ^3^ *J*_H‐H_=6.9 Hz, 12H, Dipp CH(C*H*_3_)_2_, 1.08 (d, ^3^ *J*_H‐H_=6.9 Hz, 12H, Dipp CH(C*H*_3_)_2_, 0.83 (d, ^3^ *J*_H‐H_=6.8 Hz, 6H, Dipp CH(C*H*_3_)_2_,* 0.82 (d, ^3^ *J*_H‐H_=6.8 Hz, 6H, Dipp CH(C*H*_3_)_2_.* *overlapping doublets. ^13^C{^1^H} NMR (151 MHz, C_6_D_6_): δ(ppm) 208.03 (d, ^1^ *J*_C‐P_=83.3 Hz, *C*PN_3_Dipp), 195.75 (d, ^3^ *J*_C‐P_=10.9 Hz, IDipp {HCN(Dipp)}_2_ *C*Au), 146.56 (Dipp Ar‐*C*), 145.73 (Dipp Ar‐*C*), 138.13 (d, ^2^ *J*_C‐P_=6.3 Hz, Dipp Ar*C*N), 134.66 (Dipp Ar‐*C*), 130.77 (Dipp Ar‐*C*), 128.95 (Dipp Ar‐*C*), 124.30 (Dipp Ar‐*C*), 123.45 (Dipp Ar‐*C*), 122.80 (IDipp *C*H), 29.14 (Dipp *C*H(CH_3_)_2_), 28.27 (Dipp *C*H(CH_3_)_2_), 24.99 (Dipp CH(*C*H_3_)_2_), 24.39 (Dipp CH(*C*H_3_)_2_), 24.18 (Dipp CH(*C*H_3_)_2_), 23.96 (Dipp CH(*C*H_3_)_2_). ^31^P{^1^H} NMR (162 MHz, C_6_D_6_): δ(ppm) 209.3 (s, C*P*N_3_Dipp).

*Synthesis of {Au(IDipp)}_2_{μ‐C_6_H_4_(CPN_3_)_2_} (**1d**)*: 1,3‐diazidobenzene (3.82 mg, 0.024 mmol) was added to a solution of Au(IDipp)(CP) (30 mg, 0.048 mmol) in toluene (1 mL) and stirred at room temperature overnight. The resulting precipitate was isolated by filtration. The filtrate was concentrated under vacuum, then stored at −35 °C for 3 days to yield orange crystals which were isolated by filtration. Yield: 22 mg, 0.016 mmol, 63 %. Anal. Calcd. (%) for C_62_H_78_Au_2_N_10_P_2_: C, 52.47; H, 5.54; N, 9.87. Found: 52.74; H, 5.40; N, 9.16. ^1^H NMR (600 MHz, CD_2_Cl_2_): δ(ppm)=8.03 (s, 1H; Ar*H*), 7.64 (dd, ^3^ *J*_H‐H_=8.1 Hz, ^4^ *J*_H‐H_=2.1 Hz, 2H; Ar*H*), 7.55 (t, ^3^ *J*_H‐H_=7.8 Hz, 4H; Dipp *para*‐C*H*), 7.39 (t, ^3^ *J*_H‐H_=8.1 Hz, 1H; Ar*H*), 7.36 (d, ^3^ *J*_H‐H_=7.8 Hz 8H; Dipp *meta*‐C*H*), 7.25 (s, 4H; IDipp C*H*), 2.69 (sept, ^3^ *J*_H‐H_=6.9 Hz, 8H; Dipp C*H*(CH_3_)_2_), 1.41 (d, ^3^ *J*_H‐H_=6.9 Hz, 24H; Dipp CH(C*H*_3_)), 1.26 (d, ^3^ *J*_H‐H_=6.9 Hz, 24H; Dipp CH(C*H*_3_)_2_). ^13^C{^1^H} NMR (151 MHz, CD_2_Cl_2_): δ(ppm)=208.21 (d, ^1^ *J*_C‐P_=84.1 Hz; *C*P), 194.23 (d, ^3^ *J*_C‐P_=10.0 Hz; {HCN(Dipp)}_2_ *C*Au), 146.32 (Dipp *ortho*‐*C*), 143.16 (d, *J*_C‐P_=7.6 Hz; Ar*C*) 134.77 (Dipp *ipso*‐*C*), 130.85 (Dipp *para*‐*C*), 130.36 (Ar*C*), 124.52 (Dipp *meta*‐*C*), 123.80 (IDipp C*H*), 120.73 (d, ^4^ *J*_C‐P_=5.4 Hz; Ar*C*), 115.84 (t, ^4^ *J*_C‐P_=6.5 Hz; Ar*C*), 29.28 (Dipp *C*H(CH_3_)_2_), 24.73 (Dipp CH(*C*H_3_)_2_), 24.16 (Dipp CH(*C*H_3_)_2_). ^31^P{^1^H} NMR (162 MHz, CD_2_Cl_2_): δ(ppm) 200.8 (C*P*N_3_).

*Synthesis of {Mg(^Dipp^NacNac)(CPN_3_^t^Bu)}_2_ (**2a**)*: Mg(^Dipp^NacNac)(diox)(CP) (approx. 130 mg, 0.23 mmol) was generated in situ in toluene (1 mL). Neat tert‐butyl azide (23 mg, 0.23 mmol) was added, and the solution swirled to ensure complete mixing. The reaction solution was allowed to stand overnight, resulting in the formation of colorless crystals suitable for single crystal X‐ray diffraction. The supernatant was decanted, and the crystals were washed with hexane (3×1 mL), then dried under vacuum. Yield: 120 mg, 0.10 mmol, 91 %. Anal. Calcd. (%) for C_68_H_100_Mg_2_N_10_P_2_ ⋅ C_6_H_14_: C, 70.86; H, 9.16; N, 11.17. Found: C, 70.68; H, 8.56; N, 10.84. ^1^H NMR (400 MHz, C_6_D_6_): δ(ppm) 7.12‐6.95 (m, 8H, Dipp *meta*‐CH), 6.94–6.87 (m, 4H, Dipp *para*‐CH), 4.97 (s, 2H, NacNac C*H*{C(CH_3_)(NDipp)}_2_), 3.88 (sept, ^3^ *J*_H‐H_=6.8 Hz, 4H, Dipp C*H*(CH_3_)_2_), 2.57 (sept, ^3^ *J*_H‐H_=6.8 Hz, 4H, Dipp C*H*(CH_3_)_2_), 1.67 (d, ^3^ *J*_H‐H_=6.8 Hz, 12H, Dipp CH(C*H*_3_)_2_), 1.66 (s, 12H), 1.54 (s, 18H), 1.30 (d, ^3^ *J*_H‐H_=6.8 Hz, 12H, Dipp CH(C*H*_3_)_2_), 0.99 (d, 3JH−H=6.8 Hz, 12H, Dipp CH(C*H*_3_)_2_), −0.64 (d, ^3^ *J*_H‐H_=6.8 Hz, 12H, Dipp CH(C*H*_3_)_2_). ^13^C{^1^H} NMR (126 MHz, C_6_D_6_): δ(ppm) 207.15 (d, ^1^ *J*_C‐P_=93.3 Hz, *C*PN_3_^*t*^Bu), 168.23 (NacNac CH{*C*(CH_3_)(NDipp)}_2_), 146.62 (Dipp *ipso*‐*C*), 143.17 (Dipp *ortho*‐*C*), 141.73 (Dipp *ortho*‐*C*), 124.66 (Dipp *meta*‐*C*), 124.21 (Dipp *para*‐*C*), 122.92 (Dipp *meta*‐*C*), 95.03 (NacNac *C*H{C(CH_3_)(NDipp)}_2_), 61.08 (d, ^2^ *J*_C‐P_=2.9 Hz, ^*t*^Bu *C*(CH_3_)_3_), 31.77 (d, ^3^ *J*_C‐P_=4.6 Hz, ^*t*^Bu C(*C*H_3_)_3_), 30.04 (Dipp *C*H(CH_3_)_2_), 27.60 (Dipp *C*H(CH_3_)_2_), 25.09 (Dipp CH(*C*H_3_)_2_), 24.66 (Dipp CH(*C*H_3_)_2_), 24.44 (Dipp CH(*C*H_3_)_2_), 24.23 (NacNac CH{C(*C*H_3_)(NDipp)}_2_), 24.05 (Dipp CH(*C*H_3_)_2_). ^31^P{^1^H} NMR (162 MHz, C_6_D_6_): δ(ppm) 224.3 (s, C*P*N_3_^*t*^Bu).

*Synthesis of {Mg(^Dipp^NacNac)(CPN_3_Bn)}_2_ (**2b**)*: Mg(^Dipp^NacNac)(diox.)(CP) (approx. 65 mg, 0.11 mmol) was generated in situ in toluene (1 mL). Neat benzyl azide (14 mg, 0.11 mmol) was added, and the solution swirled to ensure complete mixing. The reaction solution was allowed to stand for 3 h, resulting in the formation of colorless crystals. The supernatant was decanted, and the crystals were washed with hexane (3×1 mL), then dried under vacuum. Yield: 30 mg, 0.05 mmol, 43 %. Anal. Calcd. (%) for C_80_H_112_Mg_2_N_10_P_2_: C, 72.55; H, 8.52; N, 10.58. Found: C, 72.55; H, 8.52; N, 10.58. The low solubility of {Mg(^Dipp^NacNac)(CPN_3_Bn)}_2_ in polar organic solvents for example THF, DMSO, and dichloromethane, precluded characterization by NMR spectroscopy.

*Synthesis of Ge(^Dipp^NacNac)(CPN_3_^t^Bu) (**3**)*: Mg(^Dipp^NacNac)(diox.)(CP) (approx. 42 mg, 0.07 mmol) was generated in situ in toluene (1 mL). Ge(^Dipp^NacNac)Cl (38 mg, 0.07 mmol) was added, and the reaction mixture stirred for 3 h. The reaction mixture was filtered, then *tert*‐butyl azide (7 mg, 0.07 mmol) was added to the filtrate. The reaction solution was stirred overnight, then the solvent removed under vacuum. The residue was washed with hexane (3×1 mL), then recrystallized from toluene at −35 °C over 3 days. Yield: 32 mg, 0.06 mmol, 70 %. Anal. Calcd. (%) for C_30_H_41_GeN_2_P: C, 64.57; H, 7.97; N, 11.07. Found: C, 65.11; H, 8.28; N, 10.68. ^1^H NMR (500 MHz, C_6_D_6_): δ(ppm) 7.16‐7.09 (m, 4H, Dipp *meta*‐C*H*), 6.98 (dd, ^3^ *J*_H‐H_=6.3, 2.9 Hz, 2H, Dipp *para*‐C*H*), 5.22 (s, 1H, NacNac C*H*{C(CH_3_)(NDipp)}_2_), 3.66 (sept, ^3^ *J*_H‐H_=6.8 Hz, 2H, Dipp C*H*(CH_3_)_2_), 2.92 (sept, ^3^ *J*_H‐H_=6.8 Hz, 2H, Dipp C*H*(CH_3_)_2_), 1.63 (s, 9H, ^*t*^Bu C(C*H*_3_)_3_), 1.60 (s, 6H, NacNac CH{C(C*H*_3_)(NDipp)}_2_), 1.38 (d, ^3^ *J*_H‐H_=6.8 Hz, 6H, Dipp CH(C*H*_3_)_2_), 1.18 (d, ^3^ *J*_H‐H_=6.8 Hz, 6H, Dipp CH(C*H*_3_)_2_), 0.98 (d, ^3^ *J*_H‐H_=6.8 Hz, 6H, Dipp CH(C*H*_3_)_2_), 0.62 (d, ^3^ *J*_H‐H_=6.8 Hz, 6H, Dipp CH(C*H*_3_)_2_). ^13^C{^1^H} NMR (126 MHz, C_6_D_6_): δ(ppm) 199.39 (d, ^1^ *J*_C‐P_=105.1 Hz, *C*PN_3_^*t*^Bu), 166.67 (NacNac CH{*C*(CH_3_)(NDipp)}_2_), 146.34 (Dipp *ipso*‐*C*), 143.64 (Dipp *ortho*‐*C*), 141.58 (Dipp *ortho*‐*C*), 127.47 (Dipp *meta*‐*C*), 124.90 (Dipp *para*‐*C*), 124.41 (Dipp *meta*‐*C*), 100.13 (d, ^5^ *J*_C‐P_=6.7 Hz, NacNac *C*H{C(CH_3_)(NDipp)}_2_), 60.54 (d, ^2^ *J*_C‐P_=4.5 Hz, ^*t*^Bu *C*(CH_3_)_3_), 32.39 (d, ^2^ *J*_C‐P_=5.7 Hz, ^*t*^Bu C(*C*H_3_)_3_), 29.41 (Dipp *C*H(CH_3_)_2_), 28.70 (d, ^6^ *J*_C‐P_=3.9 Hz, Dipp *C*H(CH_3_)_2_), 26.01 (Dipp CH(*C*H_3_)_2_), 24.92 (Dipp CH(*C*H_3_)_2_), 24.73 (Dipp CH(*C*H_3_)_2_), 23.65 (NacNac CH{C(*C*H_3_)(NDipp)}_2_ and Dipp CH(*C*H_3_)_2_), 23.64 (NacNac CH{C(*C*H_3_)(NDipp)}_2_ and Dipp CH(*C*H_3_)_2_). ^31^P{^1^H} NMR (162 MHz, C_6_D_6_): δ(ppm) 196.9 (s, CPN_3_^*t*^Bu).

*Synthesis of Au(IDipp)(CPN_3_^t^Bu)(B(C_6_F_6_)_3_) (**4a**)*: Au(IDipp)(CPN_3_^*t*^Bu) (20 mg, 0.03 mmol) and B(C_6_F_5_)_3_ (14 mg, 0.03 mmol) were dissolved in toluene (0.5 mL). The reaction mixture was stirred for 1 h, then concentrated by slow evaporation. Storage at −35 °C over 7 days yielded colorless crystals which were isolated by filtration, washed with pentane (3×0.5 mL), then dried under vacuum. Yield: 19 mg, 0.02 mmol, 56 %. Anal. Calcd. (%) for C_50_H_45_AuBF_15_N_5_P ⋅ 2(C_7_H_8_): C, 54.02; H, 4.25; N, 4.92. Found: C, 53.95 H, 3.82; N, 5.25. ^1^H NMR (600 MHz, C_6_D_6_): δ(ppm) 7.34 (t, ^3^ *J*_H‐H_=7.8 Hz, 2H, Dipp *para*‐C*H*), 7.06 (d, ^3^ *J*_H‐H_=7.8 Hz, 4H, Dipp *meta*‐C*H*), 6.28 (s, 2H, IDipp C*H*), 2.55 (b, 4H, Dipp C*H*(CH_3_)_2_), 1.35 (b, 12H, Dipp CH(C*H*_3_)_2_), 1.00 (s, 9H, ^*t*^Bu C(C*H*_3_)_3_), 0.95 (d, ^3^ *J*_H‐H_=6.9 Hz, 12H, Dipp CH(C*H*_3_)_2_). ^13^C{^1^H} NMR (151 MHz, C_6_D_6_): δ(ppm) 207.25 (d, ^1^ *J*_C‐P_=77.0 Hz, *C*PN_3_^*t*^Bu), 190.92 (d, ^3^ *J*_C‐P_=3.5 Hz, IDipp {HCN(Dipp)}_2_ *C*Au), 149.88 (b, B(*C*_6_F_5_)_3_), 148.30 (b, B(*C*_6_F_5_)_3_), 145.66 (Dipp *ortho*‐*C*H), 140.69 (b, B(*C*_6_F_5_)_3_), 139.11 (b, B(*C*_6_F_5_)_3_), 137.90 (b, B(*C*_6_F_5_)_3_), 136.33 (b, B(*C*_6_F_5_)_3_), 134.54 (Dipp *ipso*‐*C*H), 130.92 (Dipp *para*‐*C*H), 124.31 (Dipp *meta*‐*C*H), 123.92 (IDipp *C*H), 63.73 (^*t*^Bu *C*(CH_3_)_3_), 30.33 (d, ^3^ *J*_C‐P_=5.1 Hz, ^*t*^Bu C(*C*H_3_)_3_), 29.01 (Dipp *C*H(CH_3_)_2_), 24.82 (Dipp CH(*C*H_3_)_2_), 23.68 (Dipp CH(*C*H_3_)_2_). ^31^P{^1^H} NMR (162 MHz, C_6_D_6_): δ(ppm) 203.5 ppm (s, C*P*N_3_^*t*^Bu). ^19^F{^1^H} NMR (377 MHz, C_6_D_6_): δ(ppm) −124.46 to −135.30 (m, B(C_6_F_5_)_3_ *ortho*‐C*F*), −156.82 to −161.17 (m, B(C_6_F_5_)_3_ *para*‐C*F*), −161.18 to −168.19 (m, B(C_6_F_5_)_3_ *meta*‐C*F*). ^11^B{^1^H} NMR (128 MHz, C_6_D_6_): δ(ppm) −5.52 ppm (b, *B*(C_6_F_5_)_3_).

*Synthesis of Au(IDipp)(CPN_3_Dipp)(B(C_6_F_6_)_3_) (**4c**)*: Au(IDipp)(CPN_3_^*t*^Bu) (20 mg, 0.03 mmol) and B(C_6_F_5_)_3_ (14 mg, 0.03 mmol) were dissolved in toluene (0.5 mL) and stirred for 1 h. The solvent was removed in vacuo and the residue extracted into CH_2_Cl_2_ (1 mL) and filtered. Storage at −35 °C over 7 days yielded colorless crystals which were isolated by filtration, washed with pentane (3×0.5 mL), then dried under vacuum. Yield: 13 mg, 0.01 mmol, 41 %. Anal. Calcd. (%) for C_58_H_54_AuBF_15_N_5_P: C, 51.80; H, 4.05; N, 5.21. Found: C, 50.88; H, 3.91; N, 5.90. ^1^H NMR (600 MHz, C_6_D_6_): δ(ppm) 7.27 (t, ^3^ *J*_H‐H_=7.8 Hz, 2H, Dipp Ar*H*), 7.04 (m, ^3^ *J*_H‐H_=7.8 Hz, 5H, Dipp Ar*H*), 6.92 (d, ^3^ *J*_H‐H_=7.8 Hz, 2H, Dipp Ar*H*), 6.27 (s, 2H, IDipp C*H*), 2.51 (b, 4H, Dipp C*H*(CH_3_)_2_), 2.27 (b, 2H, Dipp C*H*(CH_3_)_2_), 1.30 (b, 12H, Dipp CH(C*H*_3_)_2_), 1.10 (b, 6H, Dipp CH(C*H*_3_)_2_), 0.95 (d, ^3^ *J*_H‐H_=6.9 Hz, 12H, Dipp CH(C*H*_3_)_2_), 0.91 (b, 6H, Dipp CH(C*H*_3_)_2_). ^13^C{^1^H} NMR (151 MHz, C_6_D_6_): δ(ppm) 210.38 (d, ^1^ *J*_C‐P_=80.3 Hz, *C*PN_3_Dipp), 190.40 (d, ^3^ *J*_C‐P_=2.2 Hz, IDipp {HCN(Dipp)}_2_ *C*Au), 149.64 (b, B(*C*_6_F_5_)_3_), 148.04 (b, B(*C*_6_F_5_)_3_), 145.88 (Dipp Ar‐*C*), 145.58 (Dipp Ar‐*C*), 140.75 (b, B(*C*_6_F_5_)_3_), 139.11 (b, B(*C*_6_F_5_)_3_), 138.03 (b, B(*C*_6_F_5_)_3_), 136.40 (b, B(*C*_6_F_5_)_3_), 134.94 (d, ^3^ *J*_C‐P_=3.8 Hz, Dipp Ar*C*N) 134.43 (Dipp Ar‐*C*), 131.02 (Dipp Ar‐*C*), 124.30 (Dipp Ar‐*C*), 124.21 (Dipp Ar‐*C*), 123.85 (IDipp *C*H), 29.00 (Dipp *C*H(CH_3_)_2_), 28.43 (Dipp *C*H(CH_3_)_2_), 24.69 (Dipp CH(*C*H_3_)_2_), 24.55 (Dipp CH(*C*H_3_)_2_), 23.79 (Dipp CH(*C*H_3_)_2_), 23.69 (Dipp CH(*C*H_3_)_2_). One of the Dipp Ar‐*C* peaks could not be located. ^31^P{^1^H} NMR (162 MHz, C_6_D_6_): δ(ppm) 220.4 ppm (s, CPN_3_Dipp). ^19^F{^1^H} NMR (377 MHz, C_6_D_6_): δ(ppm) −124.41 to −167.21 (m, B(C_6_ *F*_5_)_3_). ^11^B{^1^H} NMR (128 MHz, C_6_D_6_): δ(ppm) −5.02 ppm (b, *B*(C_6_F_5_)_3_).

*Synthesis of HCPN_3_^t^Bu (**5**)*: {Mg(^Dipp^NacNac)(CPN_3_^*t*^Bu)}_2_ (48 mg, 0.04 mmol) and pyridinium chloride (19 mg, 0.16 mmol) were suspended in C_6_D_6_ (0.5 mL). The reaction mixture was sonicated for 1 h, after which the volatiles were distilled by vacuum transfer to an NMR tube. ^1^H NMR (600 MHz, C_6_D_6_): δ(ppm) 8.89 (d, ^2^ *J*_H‐P_=54.6 Hz, 1H, *H*CPN_3_^*t*^Bu), 1.35 (d, ^4^ *J*_H‐P_=0.6 Hz, 9H, ^*t*^Bu C(C*H*_3_)_3_). ^13^C{^1^H} NMR (151 MHz, C_6_D_6_): δ(ppm) 164.10 (d, ^1^ *J*_C‐P_=51.7 Hz, *C*PN_3_^*t*^Bu), 61.43 (d, ^2^ *J*_C‐P_=7.2 Hz, ^*t*^Bu *C*(CH_3_)_3_), 61.43 (d, ^3^ *J*_C‐P_=6.4 Hz, ^*t*^Bu C(*C*H_3_)_3_). ^31^P NMR (162 MHz, C_6_D_6_): δ(ppm) 174.9 (m, ^2^ *J*_P‐H_=55.0 Hz, HC*P*N_3_^*t*^Bu).

*Synthesis of ICPN_3_^t^Bu (**6a**)*: A solution of iodine in hexane (4 mL, 0.016 M, 0.06 mmol) was added dropwise to a stirred solution of Au(IDipp)(CPN_3_^*t*^Bu) (47 mg, 0.06 mmol) in toluene (5 mL). After stirring for 4 h, all volatiles were removed under vacuum. The residue was extracted with hexane (2×3 mL), which was then evaporated to dryness under vacuum to afford the product as an off‐white powder. Crystals suitable for single crystal X‐ray diffraction were obtained from hexane at −35 °C. Yield: 10 mg, 0.04 mmol, 58 %. Anal. Calcd. (%) for C_5_H_9_N_3_IP: C, 22.32; H, 3.37; N, 15.62. Found: C, 20.97; H, 2.67; N, 14.89. ^1^H NMR (600 MHz, C_6_D_6_): δ(ppm) 1.18 (d, ^4^ *J*_H‐P_=0.9 Hz, 9H, ^*t*^Bu C(C*H*_3_)_3_). ^13^C{^1^H} NMR (151 MHz, C_6_D_6_): δ(ppm) 120.80 (d, ^1^ *J*_C‐P_=80.6 Hz, I*C*PN_3_^*t*^Bu), 62.26 (d, ^2^ *J*_C‐P_=6.0 Hz, ^*t*^Bu *C*(CH_3_)_3_), 31.27 (d, ^3^ *J*_C‐P_=6.4 Hz, ^*t*^Bu C(*C*H_3_)_3_). ^31^P{^1^H} NMR (162 MHz, C_6_D_6_): δ(ppm) 179.9 (m, ICPN_3_^*t*^Bu).

*Synthesis of ICPN_3_Ad (**6b**)*: A solution of iodine in hexane (0.8 mL, 0.1 M, 0.08 mmol) was added to a solution of Au(IDipp)(CPN_3_Ad) (64 mg, 0.08 mmol) in toluene (1 mL), which was then stirred overnight. All volatiles were removed under vacuum, and the resulting residue was extracted with hexane (2×3 mL). The hexane solution was evaporated to dryness, yielding the product as a colorless solid. Yield: 16 mg, 0.05 mmol, 58 %. Anal. Calcd. (%) for C_11_H_15_N_3_IP ⋅ 0.5(C_6_H_6_): C, 43.53; H, 4.70; N, 10.88. Found: C, 42.96; H, 4.73; N, 10.77. ^1^H NMR (400 MHz, C_6_D_6_): δ(ppm) 1.96–1.91 (m, 6H, Ad C{C*H*_2_CHCH_2_}_3_), 1.81‐1.75 (m, 3H, Ad C{CH_2_C*H*CH_2_}_3_), 1.41–1.31 (m, 6H, Ad C{CH_2_CHC*H*_2_}_3_). ^13^C{^1^H} NMR (151 MHz, C_6_D_6_): δ(ppm) 120.50 (d, ^1^ *J*_C‐P_=80.8 Hz, I*C*PN_3_Ad), 62.90 (d, ^2^ *J*_C‐P_=5.3 Hz, Ad *C*{CH_2_CHCH_2_}_3_), 45.16 (d, ^3^ *J*_C‐P_=6.5 Hz, Ad C{*C*H_2_CHCH_2_}_3_), 35.93 (Ad C{CH_2_CH*C*H_2_}_3_), 30.06 (d, ^4^ *J*_C‐P_=0.7 Hz, Ad C{CH_2_ *C*HCH_2_}_3_). ^31^P{^1^H} NMR (162 MHz, C_6_D_6_): δ(ppm) 180.1 (m, ICPN_3_Ad).

*Synthesis of ICPN_3_Dipp (**6c**)*. A solution of iodine in hexane (0.8 mL, 0.1 M, 0.08 mmol) was added via syringe to a solution of Au(IDipp)(CPN_3_Dipp) (67 mg, 0.08 mmol) in toluene (5 mL). The solution was stirred overnight at room temperature. All volatiles were removed under vacuum, and the resulting residue was extracted with hexane (2×3 mL). The hexane solution was evaporated to dryness, yielding the product as a dark red oil. Yield: 22 mg, mmol, 76 %. Anal. Calcd. (%) for C_13_H_17_N_3_IP: C, 41.84; H, 4.59; N, 11.26. Found: C, 46.36; H, 5.25; N, 10.56. ^1^H NMR (400 MHz, C_6_D_6_): δ(ppm) 6.98 (d, 2H, ^3^ *J*_H‐H_=7.8 Hz, Dipp *meta*‐C*H*), 2.10 (sept, ^3^ *J*_H‐H_=6.8 Hz, 2H, Dipp C*H*(CH_3_)_2_), 0.91 (d, ^3^ *J*_H‐H_=6.8 Hz, 6H, Dipp CH(C*H*_3_)_2_), 0.86 (d, ^3^ *J*_H‐H_=6.9 Hz, 6H, Dipp CH(C*H*_3_)_2_). ^13^C{^1^H} NMR (151 MHz, C_6_D_6_): δ(ppm) 146.26 (d, ^3^ *J*_C‐P_=1.6 Hz, *ortho*‐Dipp *C*), 134.78 (d, ^2^ *J*_C‐P_=6.3 Hz, Ar*C*N), 130.87 (Dipp *C*), 128.35 (Ar*C*), 124.08 (Ar*C*), 123.36 (d, ^1^ *J*_C‐P_=83.5 Hz, *C*PN_3_Dipp), 28.69 (Dipp *C*H(CH_3_)_2_), 24.30 (Dipp CH(*C*H_3_)_2_, 24.07 (Dipp CH(*C*H_3_)_2_). ^31^P{^1^H} NMR (162 MHz, C_6_D_6_): δ(ppm) 194.7 (IC*P*N_3_Dipp).

Deposition Numbers 2263738 (for **1 a** ⋅ C6H6), 2263730 (for **1 b** ⋅ tol), 2263731 (for **1 c** ⋅ C6H6), 2263732 (for **1 d** ⋅ tol), 2263733 (for **2 a**), 2263734 (for **2 b** ⋅ 2tol), 2263735 (for **3**), 2263736 (for **4**), 2263737 (for **4 c** ⋅ 2CH2Cl2), 2263739 (for **6 a**), and 2263740 (for **6 b**) contain the supplementary crystallographic data for this paper. These data are provided free of charge by the joint Cambridge Crystallographic Data Centre and Fachinformationszentrum Karlsruhe Access Structures service.

## Supporting Information

Additional references cited in the Supporting Information.[^36^, ^37^, ^38^, ^39^, ^40^, ^41^, ^42^, ^43^, ^44^, ^45^, ^46^, ^47^]

## Conflict of interest

The authors declare no conflict of interest.

1

## Supporting information

As a service to our authors and readers, this journal provides supporting information supplied by the authors. Such materials are peer reviewed and may be re‐organized for online delivery, but are not copy‐edited or typeset. Technical support issues arising from supporting information (other than missing files) should be addressed to the authors.

Supporting Information

Supporting Information

## Data Availability

The data that support the findings of this study are available in the supplementary material of this article.

## References

[1] H. C. Kolb , K. B. Sharpless , Drug Discovery Today 2003, 8, 1128–1137. 14678739 10.1016/s1359-6446(03)02933-7

[2] J. F. Lutz , Angew. Chem. Int. Ed. 2007, 46, 1018–1025. 10.1002/anie.200604050 17211903

[3] M. Meldal , C. W. Tomøe , Chem. Rev. 2008, 108, 2952–3015. 18698735 10.1021/cr0783479

[4] E. M. Sletten , C. R. Bertozzi , C. R. Bertozzi , E. M. Sletten , Angew. Chem. Int. Ed. 2009, 48, 6974–6998. 10.1002/anie.200900942 PMC2864149 19714693

[5] K. Li , D. Fong , E. Meichsner , A. Adronov , Chem. Eur. J. 2021, 27, 5057–5073. 33017499 10.1002/chem.202003386

[6] V. V. Rostovtsev , L. G. Green , V. V. Fokin , K. B. Sharpless , Angew. Chem. Int. Ed. 2002, 41, 2596–2599. 10.1002/1521-3773(20020715)41:14<2596::AID-ANIE2596>3.0.CO;2-4 12203546

[7] C. W. Tornøe , C. Christensen , M. Meldal , J. Org. Chem. 2002, 67, 3057–3064. 11975567 10.1021/jo011148j

[8] J. D. Crowley , S. M. Goldup , A. L. Lee , D. A. Leigh , R. T. Mc Burney , Chem. Soc. Rev. 2009, 38, 1530–1541. 19587949 10.1039/b804243h

[9] M. Denis , S. M. Goldup , Nat. Chem. Rev. 2017, 1, 1–17.

[10] N. J. Agard , J. A. Prescher , C. R. Bertozzi , J. Am. Chem. Soc. 2004, 126, 15046–15047. 15547999 10.1021/ja044996f

[11] E. M. Sletten , C. R. Bertozzi , Acc. Chem. Res. 2011, 44, 666–676. 21838330 10.1021/ar200148z PMC3184615

[12] L. Casarrubios , M. C. De La Torre , M. A. Sierra , Chem. Eur. J. 2013, 19, 3534–3541. 23418069 10.1002/chem.201204596

[13] W. Rösch , T. Facklam , M. Regitz , Tetrahedron 1987, 43, 3247–3256.

[14] S. L. Choong , A. Nafady , A. Stasch , A. M. Bond , C. Jones , Dalton Trans. 2013, 42, 7775–7780. 23549302 10.1039/c3dt50505g

[15] J. A. W. Sklorz , S. Hoof , N. Rades , N. Derycke , L. Könczöl , D. Szieberth , M. Weber , J. Wiecko , L. Nyulászi , M. Hissler , C. Müller , Chem. Eur. J. 2015, 21, 11096–11109. 26119814 10.1002/chem.201500988

[16] M. Regitz , Chem. Rev. 1990, 90, 191–213.

[17] J. A. W. Sklorz , C. Müller , Eur. J. Inorg. Chem. 2016, 2016, 595–606.

[18] T. Görlich , D. S. Frost , N. Boback , N. T. Coles , B. Dittrich , P. Müller , W. D. Jones , C. Müller , J. Am. Chem. Soc. 2021, 143, 19365–19373. 34757730 10.1021/jacs.1c07370

[19] D. W. N. Wilson , S. J. Urwin , E. S. Yang , J. M. Goicoechea , J. Am. Chem. Soc. 2021, 143, 10367–10373. 34190545 10.1021/jacs.1c04417 PMC8297854

[20] E. S. Yang , D. W. N. Wilson , J. M. Goicoechea , Angew. Chem. Int. Ed. 2023, 62, e202218047. 10.1002/anie.202218047 PMC10946887 36656139

[21] E. S. Yang , J. M. Goicoechea , Angew. Chem. Int. Ed. 2022, 61, e202206783. 10.1002/anie.202206783 PMC9546431 35695304

[22] X. Yan , H. Wang , S. Guo , Angew. Chem. Int. Ed. 2019, 58, 16907–16911. 10.1002/anie.201911180 31502744

[23] A. Borissov , J. Y. C. Lim , A. Brown , K. E. Christensen , A. L. Thompson , M. D. Smith , P. D. Beer , Chem. Commun. 2017, 53, 2483–2486. 10.1039/c7cc00727b 28181604

[24] J. Y. C. Lim , P. D. Beer , Chem 2018, 4, 731–783.

[25] N. Kaltsoyannis , J. Chem. Soc. Dalton Trans. 1997, 1–12.

[26] L. Davin , R. McLellan , A. Hernán-Gómez , W. Clegg , A. R. Kennedy , M. Mertens , I. A. Stepek , E. Hevia , Chem. Commun. 2017, 53, 3653–3656. 10.1039/c6cc09675a 28101539

[27] Y. Yang , N. Zhao , Y. Wu , H. Zhu , H. W. Roesky , Inorg. Chem. 2012, 51, 2425–2431. 22320161 10.1021/ic202388d

[28] M. Papke , L. Dettling , J. A. W. Sklorz , D. Szieberth , L. Nyulászi , C. Müller , Angew. Chem. Int. Ed. 2017, 56, 16484–16489. 10.1002/anie.201709802 29095559

[29] E. P. O. Fuchs , M. Hermesdorf , W. Schnurr , W. Rösch , H. Heydt , M. Regitz , P. Binger , J. Organomet. Chem. 1988, 338, 329–340.

[30] W. J. Transue , A. Velian , M. Nava , M. A. Martin-Drumel , C. C. Womack , J. Jiang , G. L. Hou , X. Bin Wang , M. C. McCarthy , R. W. Field , C. C. Cummins , J. Am. Chem. Soc. 2016, 138, 6731–6734. 27171847 10.1021/jacs.6b03910

[31] A. Chen , J. Johnson , M. Lin , M. J. Shapiro , J. Am. Chem. Soc. 1998, 120, 9094–9095.

[32] P. Margaretha , S. Solar , O. E. Polansky , Angew. Chem. Int. Ed. 1971, 10, 412–413.

[33] K. Barral , A. D. Moorhouse , J. E. Moses , Org. Lett. 2007, 9, 1809–1811. 17391043 10.1021/ol070527h

[34] H. Gallardo , A. J. Bortoluzzi , D. M. P. De Oliveira Santos , Liq. Cryst. 2008, 35, 719–725.

[35] Y. Ding , H. W. Roesky , M. Noltemeyer , H. G. Schmidt , P. P. Power , Organometallics 2001, 20, 1190–1194.

[36] R. Evans , Z. Deng , A. K. Rogerson , A. S. McLachlan , J. J. Richards , M. Nilsson , G. A. Morris , Angew. Chem. Int. Ed. 2013, 52, 3199–3202. 10.1002/anie.201207403 23365047

[37] *CrysAlisPro*, Agilent Technologies, Version 1.171.41.117a.

[38a] G. M. Sheldrick , in SHELXL97, Programs for Crystal Structure Analysis (Release 97–2), Institut für Anorganische Chemie der Universität, Tammanstrasse 4, D-3400 Göttingen, Germany, 1998;

[38b] G. M. Sheldrick , Acta Crystallogr. Sect. A 1990, 46, 467–473;

[38c] G. M. Sheldrick , Acta Crystallogr. Sect. A 2008, 64, 112–122. 18156677 10.1107/S0108767307043930

[39] F. Neese , Wiley Interdiscip. Rev.: Comput. Mol. Sci. 2012, 2, 73–78.

[40] F. Neese , Wiley Interdiscip. Rev.: Comput. Mol. Sci. 2018, 8, 1–6. 10.1002/wcms.1370 PMC6220962 30450129

[41] F. Neese , F. Wennmohs , U. Becker , C. Riplinger , J. Chem. Phys. 2020, 152, 224108. 32534543 10.1063/5.0004608

[42] S. Grimme , J. Comput. Chem. 2006, 27, 1787–1799. 16955487 10.1002/jcc.20495

[43] F. Weigend , R. Ahlrichs , Phys. Chem. Chem. Phys. 2005, 7, 3297–3305. 16240044 10.1039/b508541a

[44] Y. S. Lin , G. De Li , S. P. Mao , J. Da Chai , J. Chem. Theory Comput. 2013, 9, 263–272. 26589028 10.1021/ct300715s

[45] D. A. Pantazis , X. Y. Chen , C. R. Landis , F. Neese , J. Chem. Theory Comput. 2008, 4, 908–919. 26621232 10.1021/ct800047t

[46] M. Bühl , C. Reimann , D. A. Pantazis , T. Bredow , F. Neese , J. Chem. Theory Comput. 2008, 4, 1449–1459. 26621431 10.1021/ct800172j

[47] T. Lu , F. Chen , J. Comput. Chem. 2012, 33, 580–592. 22162017 10.1002/jcc.22885

