# Supplementary material for: Cyaphide‐Azide 1,3‐Dipolar Cycloaddition Reactions: Scope and Applicability
Source: Chemistry. 2023 Aug 10;29(52):e202301648. doi: 10.1002/chem.202301648 (PMC10946888; doi:10.1002/chem.202301648)

checkCIF/PLATON report

You have not supplied any structure factors. As a result the full set of tests cannot be run.

THIS REPORT IS FOR GUIDANCE ONLY. IF USED AS PART OF A REVIEW PROCEDURE FOR PUBLICATION, IT SHOULD NOT REPLACE THE EXPERTISE OF AN EXPERIENCED CRYSTALLOGRAPHIC REFEREE.

No syntax errors found. CIF dictionary Interpreting this report

Datablock: ESY100

```
Bond precision:   C-C = 0.0165 A           Wavelength=1.54184

Cell:              a=16.2045(1)      b=10.6534(1)      c=23.8480(2)
                   alpha=90          beta=90           gamma=90
Temperature:       200 K
```

	Calculated	Reported
Volume	4116.95(6)	4116.95(6)
Space group	P n m a	P n m a
Hall group	-P 2ac 2n	-P 2ac 2n
Moiety formula	C38 H51 Au N5 P	C32 H45 Au N5 P, C6 H6
Sum formula	C38 H51 Au N5 P	C38 H51 Au N5 P
Mr	805.78	805.77
Dx,g cm-3	1.300	1.300
Z	4	4
Mu (mm-1)	7.296	7.296
F000	1632.0	1632.0
F000'	1618.79	
h,k,lmax	20,13,30	20,13,29
Nref	4558	4547
Tmin,Tmax	0.621,0.747	0.415,1.000
Tmin'	0.165	

```
Correction method= # Reported T Limits: Tmin=0.415 Tmax=1.000
AbsCorr = MULTI-SCAN

Data completeness= 0.998            Theta(max)= 76.236

R(reflections)= 0.0429( 4291)                    wR2(reflections)= 0.1214( 4547)

S = 1.270                  Npar= 328
```

The following ALERTS were generated. Each ALERT has the format
test-name_ALERT_alert-type_alert-level.
Click on the hyperlinks for more details of the test.

Alert level B

```
PLAT220_ALERT_2_B NonSolvent   Resd 1  C   Ueq(max)/Ueq(min) Range        6.2 Ratio
PLAT242_ALERT_2_B Low    'MainMol' Ueq as Compared to Neighbors of        C29 Check
PLAT330_ALERT_2_B Large Aver Phenyl C-C Dist C1S      --C6S      .       1.43 Ang.
```

Alert level C

```
PLAT042_ALERT_1_C Calc. and Reported MoietyFormula Strings  Differ     Please Check
PLAT213_ALERT_2_C Atom C31             has ADP max/min Ratio .....        3.9 prolat
PLAT215_ALERT_3_C Disordered C30       has ADP max/min Ratio .....        3.4 Note
PLAT222_ALERT_3_C NonSolvent Resd 1  H   Uiso(max)/Uiso(min) Range        6.0 Ratio
PLAT241_ALERT_2_C High   'MainMol' Ueq as Compared to Neighbors of         P1 Check
PLAT241_ALERT_2_C High   'MainMol' Ueq as Compared to Neighbors of         N3 Check
PLAT241_ALERT_2_C High   'MainMol' Ueq as Compared to Neighbors of         N5 Check
PLAT242_ALERT_2_C Low    'MainMol' Ueq as Compared to Neighbors of         N1 Check
PLAT242_ALERT_2_C Low    'MainMol' Ueq as Compared to Neighbors of         C1 Check
PLAT242_ALERT_2_C Low    'MainMol' Ueq as Compared to Neighbors of         C2 Check
PLAT260_ALERT_2_C Large Average Ueq of Residue Including       Au1      0.107 Check
PLAT342_ALERT_3_C Low Bond Precision on  C-C Bonds ...............     0.0165 Ang.
```

Alert level G

```
PLAT002_ALERT_2_G Number of Distance or Angle Restraints on AtSite          2 Note
PLAT003_ALERT_2_G Number of Uiso or Uij Restrained non-H Atoms ...          6 Report
PLAT083_ALERT_2_G SHELXL Second Parameter in WGHT  Unusually Large      18.57 Why ?
PLAT142_ALERT_4_G s.u. on b - Axis Small or Missing .............     0.00010 Ang.
PLAT143_ALERT_4_G s.u. on c - Axis Small or Missing .............     0.00020 Ang.
PLAT172_ALERT_4_G The CIF-Embedded .res File Contains DFIX Records          1 Report
PLAT177_ALERT_4_G The CIF-Embedded .res File Contains DELU Records          1 Report
PLAT178_ALERT_4_G The CIF-Embedded .res File Contains SIMU Records          1 Report
PLAT186_ALERT_4_G The CIF-Embedded .res File Contains ISOR Records          1 Report
PLAT300_ALERT_4_G Atom Site Occupancy of C1S        Constrained at        0.5 Check
PLAT300_ALERT_4_G Atom Site Occupancy of C2S        Constrained at        0.5 Check
PLAT300_ALERT_4_G Atom Site Occupancy of C3S        Constrained at        0.5 Check
PLAT300_ALERT_4_G Atom Site Occupancy of C4S        Constrained at        0.5 Check
PLAT300_ALERT_4_G Atom Site Occupancy of C5         Constrained at        0.5 Check
PLAT300_ALERT_4_G Atom Site Occupancy of C5S        Constrained at        0.5 Check
PLAT300_ALERT_4_G Atom Site Occupancy of C6         Constrained at        0.5 Check
PLAT300_ALERT_4_G Atom Site Occupancy of C6S        Constrained at        0.5 Check
PLAT300_ALERT_4_G Atom Site Occupancy of C7         Constrained at        0.5 Check
PLAT300_ALERT_4_G Atom Site Occupancy of C8         Constrained at        0.5 Check
PLAT300_ALERT_4_G Atom Site Occupancy of C9         Constrained at        0.5 Check
PLAT300_ALERT_4_G Atom Site Occupancy of C10        Constrained at        0.5 Check
PLAT300_ALERT_4_G Atom Site Occupancy of C11        Constrained at        0.5 Check
PLAT300_ALERT_4_G Atom Site Occupancy of C12        Constrained at        0.5 Check
PLAT300_ALERT_4_G Atom Site Occupancy of C13        Constrained at        0.5 Check
PLAT300_ALERT_4_G Atom Site Occupancy of C14        Constrained at        0.5 Check
PLAT300_ALERT_4_G Atom Site Occupancy of C15        Constrained at        0.5 Check
PLAT300_ALERT_4_G Atom Site Occupancy of C16        Constrained at        0.5 Check
PLAT300_ALERT_4_G Atom Site Occupancy of C17        Constrained at        0.5 Check
```

```
PLAT300_ALERT_4_G Atom Site Occupancy of C18        Constrained at        0.5 Check
PLAT300_ALERT_4_G Atom Site Occupancy of C19        Constrained at        0.5 Check
PLAT300_ALERT_4_G Atom Site Occupancy of C20        Constrained at        0.5 Check
PLAT300_ALERT_4_G Atom Site Occupancy of C21        Constrained at        0.5 Check
PLAT300_ALERT_4_G Atom Site Occupancy of C22        Constrained at        0.5 Check
PLAT300_ALERT_4_G Atom Site Occupancy of C23        Constrained at        0.5 Check
PLAT300_ALERT_4_G Atom Site Occupancy of C24        Constrained at        0.5 Check
PLAT300_ALERT_4_G Atom Site Occupancy of C25        Constrained at        0.5 Check
PLAT300_ALERT_4_G Atom Site Occupancy of C26        Constrained at        0.5 Check
PLAT300_ALERT_4_G Atom Site Occupancy of C27        Constrained at        0.5 Check
PLAT300_ALERT_4_G Atom Site Occupancy of C28        Constrained at        0.5 Check
PLAT300_ALERT_4_G Atom Site Occupancy of C30        Constrained at        0.5 Check
PLAT300_ALERT_4_G Atom Site Occupancy of H1S        Constrained at        0.5 Check
PLAT300_ALERT_4_G Atom Site Occupancy of H2S        Constrained at        0.5 Check
PLAT300_ALERT_4_G Atom Site Occupancy of H3S        Constrained at        0.5 Check
PLAT300_ALERT_4_G Atom Site Occupancy of H4S        Constrained at        0.5 Check
PLAT300_ALERT_4_G Atom Site Occupancy of H5S        Constrained at        0.5 Check
PLAT300_ALERT_4_G Atom Site Occupancy of H6S        Constrained at        0.5 Check
PLAT300_ALERT_4_G Atom Site Occupancy of H7         Constrained at        0.5 Check
PLAT300_ALERT_4_G Atom Site Occupancy of H8         Constrained at        0.5 Check
PLAT300_ALERT_4_G Atom Site Occupancy of H9         Constrained at        0.5 Check
PLAT300_ALERT_4_G Atom Site Occupancy of H11        Constrained at        0.5 Check
PLAT300_ALERT_4_G Atom Site Occupancy of H12A       Constrained at        0.5 Check
PLAT300_ALERT_4_G Atom Site Occupancy of H12B       Constrained at        0.5 Check
PLAT300_ALERT_4_G Atom Site Occupancy of H12C       Constrained at        0.5 Check
PLAT300_ALERT_4_G Atom Site Occupancy of H13A       Constrained at        0.5 Check
PLAT300_ALERT_4_G Atom Site Occupancy of H13B       Constrained at        0.5 Check
PLAT300_ALERT_4_G Atom Site Occupancy of H13C       Constrained at        0.5 Check
PLAT300_ALERT_4_G Atom Site Occupancy of H14        Constrained at        0.5 Check
PLAT300_ALERT_4_G Atom Site Occupancy of H15A       Constrained at        0.5 Check
PLAT300_ALERT_4_G Atom Site Occupancy of H15B       Constrained at        0.5 Check
PLAT300_ALERT_4_G Atom Site Occupancy of H15C       Constrained at        0.5 Check
PLAT300_ALERT_4_G Atom Site Occupancy of H16A       Constrained at        0.5 Check
PLAT300_ALERT_4_G Atom Site Occupancy of H16B       Constrained at        0.5 Check
PLAT300_ALERT_4_G Atom Site Occupancy of H16C       Constrained at        0.5 Check
PLAT300_ALERT_4_G Atom Site Occupancy of H19        Constrained at        0.5 Check
PLAT300_ALERT_4_G Atom Site Occupancy of H20        Constrained at        0.5 Check
PLAT300_ALERT_4_G Atom Site Occupancy of H21        Constrained at        0.5 Check
PLAT300_ALERT_4_G Atom Site Occupancy of H23        Constrained at        0.5 Check
PLAT300_ALERT_4_G Atom Site Occupancy of H24A       Constrained at        0.5 Check
PLAT300_ALERT_4_G Atom Site Occupancy of H24B       Constrained at        0.5 Check
PLAT300_ALERT_4_G Atom Site Occupancy of H24C       Constrained at        0.5 Check
PLAT300_ALERT_4_G Atom Site Occupancy of H25A       Constrained at        0.5 Check
PLAT300_ALERT_4_G Atom Site Occupancy of H25B       Constrained at        0.5 Check
PLAT300_ALERT_4_G Atom Site Occupancy of H25C       Constrained at        0.5 Check
PLAT300_ALERT_4_G Atom Site Occupancy of H26        Constrained at        0.5 Check
PLAT300_ALERT_4_G Atom Site Occupancy of H27A       Constrained at        0.5 Check
PLAT300_ALERT_4_G Atom Site Occupancy of H27B       Constrained at        0.5 Check
PLAT300_ALERT_4_G Atom Site Occupancy of H27C       Constrained at        0.5 Check
PLAT300_ALERT_4_G Atom Site Occupancy of H28A       Constrained at        0.5 Check
PLAT300_ALERT_4_G Atom Site Occupancy of H28B       Constrained at        0.5 Check
PLAT300_ALERT_4_G Atom Site Occupancy of H28C       Constrained at        0.5 Check
PLAT300_ALERT_4_G Atom Site Occupancy of H30A       Constrained at        0.5 Check
PLAT300_ALERT_4_G Atom Site Occupancy of H30B       Constrained at        0.5 Check
PLAT300_ALERT_4_G Atom Site Occupancy of H30C       Constrained at        0.5 Check
PLAT301_ALERT_3_G Main Residue  Disorder .............(Resd  1  )         54% Note
PLAT789_ALERT_4_G Atoms with Negative _atom_site_disorder_group  #         74 Check
```

```
PLAT811_ALERT_5_G No ADDSYM Analysis: Too Many Excluded Atoms ....          ! Info
PLAT822_ALERT_4_G CIF-embedded .res Contains Negative PART Numbers          3 Check
PLAT860_ALERT_3_G Number of Least-Squares Restraints ............          85 Note
PLAT899_ALERT_4_G SHELXL2018 is Deprecated and Succeeded by SHELXL     2019/3 Note
```

```
  0 ALERT level A = Most likely a serious problem - resolve or explain
  3 ALERT level B = A potentially serious problem, consider carefully
 12 ALERT level C = Check. Ensure it is not caused by an omission or oversight
 89 ALERT level G = General information/check it is not something unexpected

  1 ALERT type 1 CIF construction/syntax error, inconsistent or missing data
 14 ALERT type 2 Indicator that the structure model may be wrong or deficient
  5 ALERT type 3 Indicator that the structure quality may be low
 83 ALERT type 4 Improvement, methodology, query or suggestion
  1 ALERT type 5 Informative message, check
```

Datablock: ESY506

```
Bond precision:   C-C = 0.0032 A          Wavelength=1.54184

Cell:             a=20.7310(1)     b=16.1721(1)        c=26.0458(4)
                  alpha=90         beta=102.725(1)     gamma=90
Temperature:      150 K

                 Calculated                   Reported
Volume           8517.74(15)                  8517.74(15)
Space group      I 2/a                        I 2/a
Hall group       -I 2ya                       -I 2ya
Moiety formula   C38 H51 Au N5 P, C7 H8       C38 H51 Au N5 P, C7 H8
Sum formula      C45 H59 Au N5 P              C45 H59 Au N5 P
Mr               897.91                       897.90
Dx,g cm-3        1.400                        1.400
Z                8                            8
Mu (mm-1)        7.113                        7.113
F000             3664.0                       3664.0
F000'            3638.49
h,k,lmax         26,20,32                     25,20,32
Nref             8918                         8876
Tmin,Tmax        0.348,0.491                  0.642,1.000
Tmin'            0.242

Correction method= # Reported T Limits: Tmin=0.642 Tmax=1.000
AbsCorr = MULTI-SCAN
```

Data completeness= 0.995 Theta(max)= 76.151

R(reflections)= 0.0181(8207) wR2(reflections)= 0.0439(8876)

S = 1.044 Npar= 518

The following ALERTS were generated. Each ALERT has the format
test-name_ALERT_alert-type_alert-level.
Click on the hyperlinks for more details of the test.

Alert level B

```
PLAT250_ALERT_2_B Large U3/U1 Ratio for Average U(i,j) Tensor ....        4.4 Note
```

Alert level C

```
PLAT220_ALERT_2_C NonSolvent   Resd 1  C   Ueq(max)/Ueq(min) Range        3.5 Ratio
PLAT221_ALERT_2_C Solv./Anion  Resd 3  C   Ueq(max)/Ueq(min) Range        4.4 Ratio
PLAT223_ALERT_4_C Solv./Anion  Resd 3  H   Ueq(max)/Ueq(min) Range        4.3 Ratio
PLAT250_ALERT_2_C Large U3/U1 Ratio for Average U(i,j) Tensor ....        2.2 Note
PLAT331_ALERT_2_C Small Aver Phenyl C-C Dist C2R      --C7R      .       1.37 Ang.
```

Alert level G

```
PLAT002_ALERT_2_G Number of Distance or Angle Restraints on AtSite          6 Note
PLAT003_ALERT_2_G Number of Uiso or Uij Restrained non-H Atoms ...         14 Report
PLAT083_ALERT_2_G SHELXL Second Parameter in WGHT  Unusually Large       9.81 Why ?
PLAT142_ALERT_4_G s.u. on b - Axis Small or Missing .............     0.00010 Ang.
PLAT172_ALERT_4_G The CIF-Embedded .res File Contains DFIX Records          2 Report
PLAT173_ALERT_4_G The CIF-Embedded .res File Contains DANG Records          2 Report
PLAT174_ALERT_4_G The CIF-Embedded .res File Contains FLAT Records          2 Report
PLAT177_ALERT_4_G The CIF-Embedded .res File Contains DELU Records          2 Report
PLAT178_ALERT_4_G The CIF-Embedded .res File Contains SIMU Records          2 Report
PLAT186_ALERT_4_G The CIF-Embedded .res File Contains ISOR Records          1 Report
PLAT300_ALERT_4_G Atom Site Occupancy of C1R        Constrained at        0.5 Check
PLAT300_ALERT_4_G Atom Site Occupancy of C2R        Constrained at        0.5 Check
PLAT300_ALERT_4_G Atom Site Occupancy of C3R        Constrained at        0.5 Check
PLAT300_ALERT_4_G Atom Site Occupancy of C4R        Constrained at        0.5 Check
PLAT300_ALERT_4_G Atom Site Occupancy of C5R        Constrained at        0.5 Check
PLAT300_ALERT_4_G Atom Site Occupancy of C6R        Constrained at        0.5 Check
PLAT300_ALERT_4_G Atom Site Occupancy of C7R        Constrained at        0.5 Check
PLAT300_ALERT_4_G Atom Site Occupancy of H1R1       Constrained at        0.5 Check
PLAT300_ALERT_4_G Atom Site Occupancy of H1R2       Constrained at        0.5 Check
PLAT300_ALERT_4_G Atom Site Occupancy of H1R3       Constrained at        0.5 Check
PLAT300_ALERT_4_G Atom Site Occupancy of H3R        Constrained at        0.5 Check
PLAT300_ALERT_4_G Atom Site Occupancy of H4R        Constrained at        0.5 Check
PLAT300_ALERT_4_G Atom Site Occupancy of H5R        Constrained at        0.5 Check
PLAT300_ALERT_4_G Atom Site Occupancy of H6R        Constrained at        0.5 Check
PLAT300_ALERT_4_G Atom Site Occupancy of H7R        Constrained at        0.5 Check
PLAT300_ALERT_4_G Atom Site Occupancy of C1S        Constrained at        0.5 Check
PLAT300_ALERT_4_G Atom Site Occupancy of C2S        Constrained at        0.5 Check
PLAT300_ALERT_4_G Atom Site Occupancy of C3S        Constrained at        0.5 Check
PLAT300_ALERT_4_G Atom Site Occupancy of C4S        Constrained at        0.5 Check
PLAT300_ALERT_4_G Atom Site Occupancy of C5S        Constrained at        0.5 Check
```

```
PLAT300_ALERT_4_G Atom Site Occupancy of C6S       Constrained at        0.5 Check
PLAT300_ALERT_4_G Atom Site Occupancy of C7S       Constrained at        0.5 Check
PLAT300_ALERT_4_G Atom Site Occupancy of H3S       Constrained at        0.5 Check
PLAT300_ALERT_4_G Atom Site Occupancy of H1S1      Constrained at        0.5 Check
PLAT300_ALERT_4_G Atom Site Occupancy of H4S       Constrained at        0.5 Check
PLAT300_ALERT_4_G Atom Site Occupancy of H1S2      Constrained at        0.5 Check
PLAT300_ALERT_4_G Atom Site Occupancy of H5S       Constrained at        0.5 Check
PLAT300_ALERT_4_G Atom Site Occupancy of H1S3      Constrained at        0.5 Check
PLAT300_ALERT_4_G Atom Site Occupancy of H6S       Constrained at        0.5 Check
PLAT300_ALERT_4_G Atom Site Occupancy of H7S       Constrained at        0.5 Check
PLAT302_ALERT_4_G Anion/Solvent/Minor-Residue Disorder (Resd  2  )       100% Note
PLAT302_ALERT_4_G Anion/Solvent/Minor-Residue Disorder (Resd  3  )       100% Note
PLAT304_ALERT_4_G Non-Integer Number of Atoms in ..... (Resd  2  )       7.50 Check
PLAT304_ALERT_4_G Non-Integer Number of Atoms in ..... (Resd  3  )       7.50 Check
PLAT380_ALERT_4_G Incorrectly? Oriented X(sp2)-Methyl Moiety .....        C1R Check
PLAT380_ALERT_4_G Incorrectly? Oriented X(sp2)-Methyl Moiety .....        C1S Check
PLAT720_ALERT_4_G Number of Unusual/Non-Standard Labels ..........          6 Note
PLAT789_ALERT_4_G Atoms with Negative _atom_site_disorder_group  #         30 Check
PLAT822_ALERT_4_G CIF-embedded .res Contains Negative PART Numbers          1 Check
PLAT860_ALERT_3_G Number of Least-Squares Restraints .............        132 Note
PLAT899_ALERT_4_G SHELXL2018 is Deprecated and Succeeded by SHELXL     2019/3 Note
```

---

 0 **ALERT level A** = Most likely a serious problem - resolve or explain
 1 **ALERT level B** = A potentially serious problem, consider carefully
 5 **ALERT level C** = Check. Ensure it is not caused by an omission or oversight
51 **ALERT level G** = General information/check it is not something unexpected

 0 ALERT type 1 CIF construction/syntax error, inconsistent or missing data
 8 ALERT type 2 Indicator that the structure model may be wrong or deficient
 1 ALERT type 3 Indicator that the structure quality may be low
48 ALERT type 4 Improvement, methodology, query or suggestion
 0 ALERT type 5 Informative message, check

Datablock: AM010

Bond precision: C-C = 0.0072 A Wavelength=1.54184

Cell:	a=12.5798(2)	b=16.7485(2)	c=22.2537(3)
	alpha=90	beta=106.371(2)	gamma=90
Temperature:	150 K		

	Calculated	Reported
Volume	4498.60(12)	4498.60(12)
Space group	P 21/c	P 21/c
Hall group	-P 2ybc	-P 2ybc
Moiety formula	C40 H53 Au N5 P, C6 H6	C40 H53 Au N5 P, C6 H6
Sum formula	C46 H59 Au N5 P	C46 H59 Au N5 P
Mr	909.92	909.91
Dx,g cm-3	1.344	1.343
Z	4	4
Mu (mm-1)	6.742	6.742
F000	1856.0	1856.0
F000'	1843.31	
h,k,lmax	15,21,28	15,21,27
Nref	9406	9366
Tmin,Tmax	0.550,0.874	0.644,1.000
Tmin'	0.283	

Correction method= # Reported T Limits: Tmin=0.644 Tmax=1.000
AbsCorr = MULTI-SCAN

Data completeness= 0.996 Theta(max)= 76.218

R(reflections)= 0.0362(7990) wR2(reflections)= 0.0944(9366)

S = 1.062 Npar= 516

The following ALERTS were generated. Each ALERT has the format
test-name_ALERT_alert-type_alert-level.
Click on the hyperlinks for more details of the test.

Alert level B

```
PLAT220_ALERT_2_B NonSolvent   Resd 1  C   Ueq(max)/Ueq(min) Range        7.0 Ratio
PLAT242_ALERT_2_B Low    'MainMol' Ueq as Compared to Neighbors of        C38 Check
```

Alert level C

```
PLAT094_ALERT_2_C Ratio of Maximum / Minimum Residual Density ....       2.38 Report
PLAT213_ALERT_2_C Atom C40              has ADP max/min Ratio .....       3.2 prolat
PLAT222_ALERT_3_C NonSolvent Resd 1  H   Uiso(max)/Uiso(min) Range        6.6 Ratio
PLAT242_ALERT_2_C Low    'MainMol' Ueq as Compared to Neighbors of        C23 Check
PLAT250_ALERT_2_C Large U3/U1 Ratio for Average U(i,j) Tensor ....        3.1 Note
PLAT260_ALERT_2_C Large Average Ueq of Residue Including      C1T      0.121 Check
PLAT601_ALERT_2_C Unit Cell Contains Solvent Accessible VOIDS of .         34 Ang**3
```

Alert level G

```
PLAT083_ALERT_2_G SHELXL Second Parameter in WGHT  Unusually Large       9.14 Why ?
```

```
PLAT302_ALERT_4_G Anion/Solvent/Minor-Residue Disorder (Resd  2  )       100% Note
PLAT302_ALERT_4_G Anion/Solvent/Minor-Residue Disorder (Resd  3  )       100% Note
PLAT304_ALERT_4_G Non-Integer Number of Atoms in ..... (Resd  2  )       6.36 Check
PLAT304_ALERT_4_G Non-Integer Number of Atoms in ..... (Resd  3  )       5.64 Check
PLAT899_ALERT_4_G SHELXL2018 is Deprecated and Succeeded by SHELXL     2019/3 Note
```

---

0 **ALERT level A** = Most likely a serious problem - resolve or explain
2 **ALERT level B** = A potentially serious problem, consider carefully
7 **ALERT level C** = Check. Ensure it is not caused by an omission or oversight
6 **ALERT level G** = General information/check it is not something unexpected

0 ALERT type 1 CIF construction/syntax error, inconsistent or missing data
9 ALERT type 2 Indicator that the structure model may be wrong or deficient
1 ALERT type 3 Indicator that the structure quality may be low
5 ALERT type 4 Improvement, methodology, query or suggestion
0 ALERT type 5 Informative message, check

Datablock: AM013B

Bond precision: C-C = 0.0175 A   Wavelength=1.54184

Cell:	a=14.1752(4)	b=21.0565(4)	c=24.3488(6)
	alpha=94.882(2)	beta=90.052(2)	gamma=101.648(2)
Temperature:	150 K		

	Calculated	Reported
Volume	7090.9(3)	7090.9(3)
Space group	P -1	P -1
Hall group	-P 1	-P 1
Moiety formula	C62 H76 Au2 N10 P2, C7 H8 [+ solvent]	C62 H76 Au2 N10 P2, C7 H8
Sum formula	C69 H84 Au2 N10 P2 [+ solvent]	C69 H84 Au2 N10 P2
Mr	1509.35	1509.33
Dx,g cm-3	1.414	1.414
Z	4	4
Mu (mm-1)	8.436	8.436
F000	3024.0	3024.0
F000’	2997.15	
h,k,lmax	16,25,28	16,25,28
Nref	24990	24975
Tmin,Tmax	0.467,0.845	0.624,1.000
Tmin’	0.209	

Correction method= # Reported T Limits: Tmin=0.624 Tmax=1.000
AbsCorr = MULTI-SCAN

Data completeness= 0.999 Theta(max)= 66.499

R(reflections)= 0.0652(18771) wR2(reflections)= 0.1933(24975)

S = 1.106 Npar= 1526

The following ALERTS were generated. Each ALERT has the format
test-name_ALERT_alert-type_alert-level.
Click on the hyperlinks for more details of the test.

Alert level B

```
PLAT213_ALERT_2_B Atom C59              has ADP max/min Ratio .....        4.6 prolat
PLAT220_ALERT_2_B NonSolvent   Resd 1  C   Ueq(max)/Ueq(min) Range        7.1 Ratio
PLAT220_ALERT_2_B NonSolvent   Resd 2  C   Ueq(max)/Ueq(min) Range        7.6 Ratio
PLAT242_ALERT_2_B Low    'MainMol' Ueq as Compared to Neighbors of        C30 Check
PLAT242_ALERT_2_B Low    'MainMol' Ueq as Compared to Neighbors of        C48 Check
PLAT242_ALERT_2_B Low    'MainMol' Ueq as Compared to Neighbors of        C57 Check
PLAT242_ALERT_2_B Low    'MainMol' Ueq as Compared to Neighbors of       C148 Check
PLAT250_ALERT_2_B Large U3/U1 Ratio for Average U(i,j) Tensor ....        5.7 Note
```

Alert level C

```
PLAT213_ALERT_2_C Atom C32              has ADP max/min Ratio .....        3.3 prolat
PLAT213_ALERT_2_C Atom C149             has ADP max/min Ratio .....        3.6 prolat
PLAT213_ALERT_2_C Atom C150             has ADP max/min Ratio .....        4.0 prolat
PLAT222_ALERT_3_C NonSolvent Resd 1  H   Uiso(max)/Uiso(min) Range        8.0 Ratio
PLAT222_ALERT_3_C NonSolvent Resd 2  H   Uiso(max)/Uiso(min) Range        9.4 Ratio
PLAT234_ALERT_4_C Large Hirshfeld Difference C25      --C26      .       0.17 Ang.
PLAT234_ALERT_4_C Large Hirshfeld Difference C27      --C28      .       0.24 Ang.
PLAT234_ALERT_4_C Large Hirshfeld Difference C153     --C154     .       0.16 Ang.
PLAT242_ALERT_2_C Low    'MainMol' Ueq as Compared to Neighbors of        C21 Check
PLAT242_ALERT_2_C Low    'MainMol' Ueq as Compared to Neighbors of        C33 Check
PLAT242_ALERT_2_C Low    'MainMol' Ueq as Compared to Neighbors of        C45 Check
PLAT242_ALERT_2_C Low    'MainMol' Ueq as Compared to Neighbors of       C121 Check
PLAT242_ALERT_2_C Low    'MainMol' Ueq as Compared to Neighbors of       C130 Check
PLAT242_ALERT_2_C Low    'MainMol' Ueq as Compared to Neighbors of       C133 Check
PLAT242_ALERT_2_C Low    'MainMol' Ueq as Compared to Neighbors of       C145 Check
PLAT243_ALERT_4_C High   'Solvent' Ueq as Compared to Neighbors of        C4T Check
PLAT244_ALERT_4_C Low    'Solvent' Ueq as Compared to Neighbors of        C2T Check
PLAT244_ALERT_4_C Low    'Solvent' Ueq as Compared to Neighbors of        C3T Check
PLAT244_ALERT_4_C Low    'Solvent' Ueq as Compared to Neighbors of        C5T Check
PLAT250_ALERT_2_C Large U3/U1 Ratio for Average U(i,j) Tensor ....        2.7 Note
PLAT260_ALERT_2_C Large Average Ueq of Residue Including       C1R      0.117 Check
PLAT260_ALERT_2_C Large Average Ueq of Residue Including       C1T      0.289 Check
PLAT260_ALERT_2_C Large Average Ueq of Residue Including       C1S      0.145 Check
PLAT342_ALERT_3_C Low Bond Precision on  C-C Bonds ...............    0.01746 Ang.
PLAT360_ALERT_2_C Short  C(sp3)-C(sp3) Bond  C30      - C31      .       1.43 Ang.
PLAT360_ALERT_2_C Short  C(sp3)-C(sp3) Bond  C30      - C32      .       1.39 Ang.
PLAT360_ALERT_2_C Short  C(sp3)-C(sp3) Bond  C48      - C50      .       1.42 Ang.
```

Alert level G

```
PLAT002_ALERT_2_G Number of Distance or Angle Restraints on AtSite         21 Note
PLAT003_ALERT_2_G Number of Uiso or Uij Restrained non-H Atoms ...         25 Report
PLAT083_ALERT_2_G SHELXL Second Parameter in WGHT  Unusually Large      15.88 Why ?
PLAT154_ALERT_1_G The s.u.'s on the Cell Angles are Equal ..(Note)      0.002 Degree
PLAT172_ALERT_4_G The CIF-Embedded .res File Contains DFIX Records         11 Report
PLAT175_ALERT_4_G The CIF-Embedded .res File Contains SAME Records          2 Report
PLAT177_ALERT_4_G The CIF-Embedded .res File Contains DELU Records          4 Report
PLAT178_ALERT_4_G The CIF-Embedded .res File Contains SIMU Records          4 Report
PLAT186_ALERT_4_G The CIF-Embedded .res File Contains ISOR Records          2 Report
PLAT232_ALERT_2_G Hirshfeld Test Diff (M-X)  Au2      --C2       .        6.6 s.u.
PLAT302_ALERT_4_G Anion/Solvent/Minor-Residue Disorder (Resd  3  )       100% Note
PLAT302_ALERT_4_G Anion/Solvent/Minor-Residue Disorder (Resd  5  )       100% Note
PLAT304_ALERT_4_G Non-Integer Number of Atoms in ..... (Resd  3  )       8.25 Check
PLAT304_ALERT_4_G Non-Integer Number of Atoms in ..... (Resd  5  )       6.75 Check
PLAT380_ALERT_4_G Incorrectly? Oriented X(sp2)-Methyl Moiety .....        C1R Check
PLAT380_ALERT_4_G Incorrectly? Oriented X(sp2)-Methyl Moiety .....        C1T Check
PLAT606_ALERT_4_G Solvent Accessible VOID(S) in Structure ........          ! Info
PLAT720_ALERT_4_G Number of Unusual/Non-Standard Labels ..........          9 Note
PLAT860_ALERT_3_G Number of Least-Squares Restraints .............        277 Note
PLAT869_ALERT_4_G ALERTS Related to the Use of SQUEEZE  Suppressed          ! Info
PLAT899_ALERT_4_G SHELXL2018 is Deprecated and Succeeded by SHELXL     2019/3 Note
PLAT941_ALERT_3_G Average HKL Measurement Multiplicity ...........        2.9 Low
PLAT967_ALERT_5_G Note: Two-Theta Cutoff Value in Embedded .res ..      133.0 Degree
```

```
 0 ALERT level A = Most likely a serious problem - resolve or explain
 8 ALERT level B = A potentially serious problem, consider carefully
27 ALERT level C = Check. Ensure it is not caused by an omission or oversight
23 ALERT level G = General information/check it is not something unexpected

 1 ALERT type 1 CIF construction/syntax error, inconsistent or missing data
29 ALERT type 2 Indicator that the structure model may be wrong or deficient
 5 ALERT type 3 Indicator that the structure quality may be low
22 ALERT type 4 Improvement, methodology, query or suggestion
 1 ALERT type 5 Informative message, check
```

Datablock: ESY026

```
Bond precision:   C-C = 0.0022 A          Wavelength=1.54184

Cell:             a=18.1532(2)     b=19.1427(2)        c=12.4393(1)
                  alpha=90         beta=122.232(1)     gamma=90
Temperature:      150 K
```

	Calculated	Reported
Volume	3656.53(7)	3656.53(7)
Space group	C 2/m	C 2/m
Hall group	-C 2y	-C 2y
Moiety formula	C68 H100 Mg2 N10 P2 [+ solvent]	C68 H100 Mg2 N10 P2
Sum formula	C68 H100 Mg2 N10 P2 [+ solvent]	C68 H100 Mg2 N10 P2
Mr	1168.14	1168.13
Dx,g cm-3	1.061	1.061
Z	2	2
Mu (mm-1)	1.033	1.033
F000	1264.0	1264.0
F000'	1268.68	
h,k,lmax	22,24,15	22,24,15
Nref	3975	3960
Tmin,Tmax	0.928,0.960	0.957,1.000
Tmin'	0.830	

Correction method= # Reported T Limits: Tmin=0.957 Tmax=1.000
AbsCorr = MULTI-SCAN

Data completeness= 0.996 Theta(max)= 76.518

R(reflections)= 0.0346(3659) wR2(reflections)= 0.1047(3960)

S = 1.051 Npar= 199

The following ALERTS were generated. Each ALERT has the format
test-name_ALERT_alert-type_alert-level.
Click on the hyperlinks for more details of the test.

Alert level C

PLAT329_ALERT_4_C Carbon Atom Hybridisation Unclear for .... C4 Check

---

Alert level G

PLAT128_ALERT_4_G Alternate Setting for Input Space Group C2/m I2/m Note
PLAT143_ALERT_4_G s.u. on c - Axis Small or Missing .... 0.00010 Ang.
PLAT232_ALERT_2_G Hirshfeld Test Diff (M-X) Mg1 --C1 . 6.1 s.u.
PLAT300_ALERT_4_G Atom Site Occupancy of H4B Constrained at 0.5 Check
PLAT300_ALERT_4_G Atom Site Occupancy of H4C Constrained at 0.5 Check
PLAT343_ALERT_2_G Unusual sp? Angle Range in Main Residue for C2 Check
PLAT343_ALERT_2_G Unusual sp? Angle Range in Main Residue for C5 Check
PLAT367_ALERT_2_G Long? C(sp?)-C(sp?) Bond C2 - C3 . 1.52 Ang.
PLAT367_ALERT_2_G Long? C(sp?)-C(sp?) Bond C2 - C4 . 1.53 Ang.
PLAT380_ALERT_4_G Incorrectly? Oriented X(sp2)-Methyl Moiety ..... C7 Check

```
PLAT605_ALERT_4_G Largest Solvent Accessible VOID in the Structure        214 A**3
PLAT764_ALERT_4_G Overcomplete CIF Bond List Detected (Rep/Expd) .       1.12 Ratio
PLAT869_ALERT_4_G ALERTS Related to the Use of SQUEEZE  Suppressed          ! Info
PLAT899_ALERT_4_G SHELXL2018 is Deprecated and Succeeded by SHELXL     2019/3 Note
PLAT941_ALERT_3_G Average HKL Measurement Multiplicity ..........         4.7 Low
```

```
   0 ALERT level A = Most likely a serious problem - resolve or explain
   0 ALERT level B = A potentially serious problem, consider carefully
   1 ALERT level C = Check. Ensure it is not caused by an omission or oversight
  15 ALERT level G = General information/check it is not something unexpected

   0 ALERT type 1 CIF construction/syntax error, inconsistent or missing data
   5 ALERT type 2 Indicator that the structure model may be wrong or deficient
   1 ALERT type 3 Indicator that the structure quality may be low
  10 ALERT type 4 Improvement, methodology, query or suggestion
   0 ALERT type 5 Informative message, check
```

Datablock: ESY064

Bond precision: C-C = 0.0053 A Wavelength=1.54184

Cell:	a=13.0652(5)	b=13.1025(6)	c=14.2674(7)
	alpha=114.624(4)	beta=113.693(4)	gamma=95.063(4)
Temperature:	150 K		

	Calculated	Reported
Volume	1934.9(2)	1934.86(17)
Space group	P -1	P -1
Hall group	-P 1	-P 1
Moiety formula	C74 H96 Mg2 N10 P2, C7 H8	C67 H88 Mg2 N10 P2, 2(C7 H8)
Sum formula	C81 H104 Mg2 N10 P2	C81 H104 Mg2 N10 P2
Mr	1328.30	1328.30
Dx,g cm-3	1.140	1.140
Z	1	1
Mu (mm-1)	1.037	1.037
F000	714.0	714.0
F000’	716.56	
h,k,lmax	16,16,17	16,16,17
Nref	8093	7813
Tmin,Tmax	0.883,0.940	0.979,1.000
Tmin’	0.847	

Correction method= # Reported T Limits: Tmin=0.979 Tmax=1.000
AbsCorr = MULTI-SCAN

```
Data completeness= 0.965            Theta(max)= 76.095

                                                     wR2(reflections)=
R(reflections)= 0.0611( 7332)                        0.1621( 7813)

S = 1.126                  Npar= 459
```

The following ALERTS were generated. Each ALERT has the format
test-name_ALERT_alert-type_alert-level.
Click on the hyperlinks for more details of the test.

Alert level C

```
PLAT042_ALERT_1_C Calc. and Reported MoietyFormula Strings  Differ     Please Check
PLAT220_ALERT_2_C NonSolvent   Resd 1   C    Ueq(max)/Ueq(min) Range        3.1 Ratio
PLAT250_ALERT_2_C Large U3/U1 Ratio for Average U(i,j) Tensor ....        3.1 Note
PLAT260_ALERT_2_C Large Average Ueq of Residue Including       C1S      0.116 Check
PLAT340_ALERT_3_C Low Bond Precision on  C-C Bonds ...............    0.00531 Ang.
```

Alert level G

```
PLAT002_ALERT_2_G Number of Distance or Angle Restraints on AtSite          7 Note
PLAT003_ALERT_2_G Number of Uiso or Uij Restrained non-H Atoms ...          7 Report
PLAT152_ALERT_1_G The Supplied and Calc. Volume s.u. Differ by ...          3 Units
PLAT154_ALERT_1_G The s.u.'s on the Cell Angles are Equal ..(Note)      0.004 Degree
PLAT172_ALERT_4_G The CIF-Embedded .res File Contains DFIX Records          9 Report
PLAT177_ALERT_4_G The CIF-Embedded .res File Contains DELU Records          1 Report
PLAT178_ALERT_4_G The CIF-Embedded .res File Contains SIMU Records          1 Report
PLAT300_ALERT_4_G Atom Site Occupancy of C1S        Constrained at        0.5 Check
PLAT300_ALERT_4_G Atom Site Occupancy of C2S        Constrained at        0.5 Check
PLAT300_ALERT_4_G Atom Site Occupancy of C3S        Constrained at        0.5 Check
PLAT300_ALERT_4_G Atom Site Occupancy of C4S        Constrained at        0.5 Check
PLAT300_ALERT_4_G Atom Site Occupancy of C5S        Constrained at        0.5 Check
PLAT300_ALERT_4_G Atom Site Occupancy of C6S        Constrained at        0.5 Check
PLAT300_ALERT_4_G Atom Site Occupancy of C7S        Constrained at        0.5 Check
PLAT300_ALERT_4_G Atom Site Occupancy of H1S1       Constrained at        0.5 Check
PLAT300_ALERT_4_G Atom Site Occupancy of H1S2       Constrained at        0.5 Check
PLAT300_ALERT_4_G Atom Site Occupancy of H1S3       Constrained at        0.5 Check
PLAT300_ALERT_4_G Atom Site Occupancy of H3S        Constrained at        0.5 Check
PLAT300_ALERT_4_G Atom Site Occupancy of H4S        Constrained at        0.5 Check
PLAT300_ALERT_4_G Atom Site Occupancy of H5S        Constrained at        0.5 Check
PLAT300_ALERT_4_G Atom Site Occupancy of H6S        Constrained at        0.5 Check
PLAT300_ALERT_4_G Atom Site Occupancy of H7S        Constrained at        0.5 Check
PLAT302_ALERT_4_G Anion/Solvent/Minor-Residue Disorder (Resd  2  )       100% Note
PLAT304_ALERT_4_G Non-Integer Number of Atoms in ..... (Resd  2  )       7.50 Check
PLAT343_ALERT_2_G Unusual sp?      Angle Range in Main Residue for         C1 Check
PLAT380_ALERT_4_G Incorrectly? Oriented X(sp2)-Methyl Moiety .....        C1S Check
PLAT720_ALERT_4_G Number of Unusual/Non-Standard Labels ..........          3 Note
PLAT789_ALERT_4_G Atoms with Negative _atom_site_disorder_group  #         15 Check
PLAT822_ALERT_4_G CIF-embedded .res Contains Negative PART Numbers          1 Check
PLAT860_ALERT_3_G Number of Least-Squares Restraints .............         66 Note
PLAT899_ALERT_4_G SHELXL2018 is Deprecated and Succeeded by SHELXL     2019/3 Note
PLAT941_ALERT_3_G Average HKL Measurement Multiplicity ...........        2.4 Low
```

---

0 **ALERT level A** = Most likely a serious problem - resolve or explain
0 **ALERT level B** = A potentially serious problem, consider carefully
5 **ALERT level C** = Check. Ensure it is not caused by an omission or oversight
32 **ALERT level G** = General information/check it is not something unexpected

3 ALERT type 1 CIF construction/syntax error, inconsistent or missing data
6 ALERT type 2 Indicator that the structure model may be wrong or deficient
3 ALERT type 3 Indicator that the structure quality may be low
25 ALERT type 4 Improvement, methodology, query or suggestion
0 ALERT type 5 Informative message, check

Datablock: ESY153

Bond precision: C-C = 0.0022 A Wavelength=1.54184

Cell:	a=18.5981(1)	b=18.5981(1)	c=20.7899(1)
	alpha=90	beta=90	gamma=90
Temperature:	150 K		

	Calculated	Reported
Volume	7191.01(8)	7191.00(8)
Space group	P 42/n	P 42/n
Hall group	-P 4bc	-P 4bc
Moiety formula	C34 H50 Ge N5 P	C34 H50 Ge N5 P
Sum formula	C34 H50 Ge N5 P	C34 H50 Ge N5 P
Mr	632.37	632.35
Dx,g cm-3	1.168	1.168
Z	8	8
Mu (mm-1)	1.783	1.783
F000	2688.0	2688.0
F000'	2687.22	
h,k,lmax	23,23,26	23,23,26
Nref	7573	7522
Tmin,Tmax	0.684,0.700	0.858,1.000
Tmin'	0.621	

Correction method= # Reported T Limits: Tmin=0.858 Tmax=1.000
AbsCorr = MULTI-SCAN

Data completeness= 0.993 Theta(max)= 76.453

R(reflections)= 0.0301(6575) wR2(reflections)= 0.0869(7522)

S = 1.038 Npar= 408

The following ALERTS were generated. Each ALERT has the format
test-name_ALERT_alert-type_alert-level.
Click on the hyperlinks for more details of the test.

Alert level B

```
PLAT110_ALERT_2_B ADDSYM Detects Potential Lattice Translation ...          ? Check
PLAT112_ALERT_2_B ADDSYM Detects New (Pseudo) Symm. Elem         I        100 %Fit
PLAT113_ALERT_2_B ADDSYM Suggests Possible Pseudo/New  Space Group       I4/m Check
              Check Model Parameter Symmmetry for Reflection Data Support
PLAT601_ALERT_2_B Unit Cell Contains Solvent Accessible VOIDS of .        105 Ang**3
```

Alert level C

```
PLAT213_ALERT_2_C Atom C4A             has ADP max/min Ratio .....        3.1 prolat
PLAT220_ALERT_2_C NonSolvent   Resd 1  C   Ueq(max)/Ueq(min) Range        3.6 Ratio
PLAT222_ALERT_3_C NonSolvent Resd 1  H   Uiso(max)/Uiso(min) Range        4.3 Ratio
PLAT242_ALERT_2_C Low    'MainMol' Ueq as Compared to Neighbors of         C2 Check
```

Alert level G

```
PLAT116_ALERT_2_G ADDSYM Included (Pseudo) Lattice Translation ...     Please Check
PLAT143_ALERT_4_G s.u. on c - Axis Small or Missing .............    0.00010 Ang.
PLAT301_ALERT_3_G Main Residue  Disorder .............(Resd  1  )         7% Note
PLAT720_ALERT_4_G Number of Unusual/Non-Standard Labels ..........          9 Note
PLAT899_ALERT_4_G SHELXL2018 is Deprecated and Succeeded by SHELXL     2019/3 Note
```

0 **ALERT level A** = Most likely a serious problem - resolve or explain
4 **ALERT level B** = A potentially serious problem, consider carefully
4 **ALERT level C** = Check. Ensure it is not caused by an omission or oversight
5 **ALERT level G** = General information/check it is not something unexpected

0 ALERT type 1 CIF construction/syntax error, inconsistent or missing data
8 ALERT type 2 Indicator that the structure model may be wrong or deficient
2 ALERT type 3 Indicator that the structure quality may be low
3 ALERT type 4 Improvement, methodology, query or suggestion
0 ALERT type 5 Informative message, check

Datablock: ESY338

Bond precision: C-C = 0.0083 A Wavelength=1.54184

Cell:	a=14.6085(2)	b=18.1164(2)	c=19.2324(3)
	alpha=90	beta=90	gamma=90
Temperature:	150 K		

	Calculated	Reported
Volume	5089.92(12)	5089.92(12)
Space group	P 21 21 21	P 21 21 21
Hall group	P 2ac 2ab	P 2ac 2ab
Moiety formula	C50 H45 Au B F15 N5 P	C50 H45 Au B F15 N5 P
Sum formula	C50 H45 Au B F15 N5 P	C50 H45 Au B F15 N5 P
Mr	1239.66	1239.65
Dx,g cm-3	1.618	1.618
Z	4	4
Mu (mm-1)	6.576	6.576
F000	2456.0	2456.0
F000'	2447.91	
h,k,lmax	18,22,24	18,22,24
Nref	10686[5886]	9631
Tmin,Tmax	0.183,0.306	0.526,1.000
Tmin'	0.081	

Correction method= # Reported T Limits: Tmin=0.526 Tmax=1.000
AbsCorr = MULTI-SCAN

Data completeness= 1.64/0.90 Theta(max)= 76.384

R(reflections)= 0.0230(9327) wR2(reflections)= 0.0584(9631)

S = 1.051 Npar= 669

The following ALERTS were generated. Each ALERT has the format
test-name_ALERT_alert-type_alert-level.
Click on the hyperlinks for more details of the test.

Alert level B

PLAT220_ALERT_2_B NonSolvent Resd 1 C Ueq(max)/Ueq(min) Range 7.5 Ratio
PLAT242_ALERT_2_B Low 'MainMol' Ueq as Compared to Neighbors of C11 Check

---

Alert level C

PLAT094_ALERT_2_C Ratio of Maximum / Minimum Residual Density .... 2.26 Report
PLAT222_ALERT_3_C NonSolvent Resd 1 H Uiso(max)/Uiso(min) Range 6.1 Ratio
PLAT241_ALERT_2_C High 'MainMol' Ueq as Compared to Neighbors of C8 Check
PLAT242_ALERT_2_C Low 'MainMol' Ueq as Compared to Neighbors of C23 Check
PLAT342_ALERT_3_C Low Bond Precision on C-C Bonds .... 0.00833 Ang.

Alert level G

PLAT899_ALERT_4_G SHELXL2018 is Deprecated and Succeeded by SHELXL 2019/3 Note

---

0 **ALERT level A** = Most likely a serious problem - resolve or explain

2 **ALERT level B** = A potentially serious problem, consider carefully
5 **ALERT level C** = Check. Ensure it is not caused by an omission or oversight
1 **ALERT level G** = General information/check it is not something unexpected

0 ALERT type 1 CIF construction/syntax error, inconsistent or missing data
5 ALERT type 2 Indicator that the structure model may be wrong or deficient
2 ALERT type 3 Indicator that the structure quality may be low
1 ALERT type 4 Improvement, methodology, query or suggestion
0 ALERT type 5 Informative message, check

Datablock: AM026

Bond precision: C-C = 0.0062 A Wavelength=1.54184

Cell:	a=12.4556(2)	b=23.8012(3)	c=21.2193(2)
	alpha=90	beta=93.566(1)	gamma=90
Temperature:	150 K		

	Calculated	Reported
Volume	6278.46(14)	6278.46(14)
Space group	P 21/n	P 21/n
Hall group	-P 2yn	-P 2yn
Moiety formula	C58 H53 Au B F15 N5 P, 2(C H2 Cl2)	C58 H53 Au B F15 N5 P, 2(C H2 Cl2)
Sum formula	C60 H57 Au B Cl4 F15 N5 P	C60 H57 Au B Cl4 F15 N5 P
Mr	1513.66	1513.65
Dx,g cm-3	1.601	1.601
Z	4	4
Mu (mm-1)	6.979	6.979
F000	3016.0	3016.0
F000'	3014.55	
h,k,lmax	15,29,26	15,29,26
Nref	13156	13093
Tmin,Tmax	0.751,0.756	0.704,1.000
Tmin'	0.205	

Correction method= # Reported T Limits: Tmin=0.704 Tmax=1.000
AbsCorr = MULTI-SCAN

Data completeness= 0.995 Theta(max)= 76.307

R(reflections)= 0.0354(10875) wR2(reflections)= 0.0885(13093)

S = 1.076 Npar= 784

The following ALERTS were generated. Each ALERT has the format
test-name_ALERT_alert-type_alert-level.
Click on the hyperlinks for more details of the test.

Alert level C

```
PLAT213_ALERT_2_C Atom F13              has ADP max/min Ratio .....         3.2 prolat
PLAT220_ALERT_2_C NonSolvent   Resd 1   C   Ueq(max)/Ueq(min) Range         4.0 Ratio
PLAT242_ALERT_2_C Low    'MainMol' Ueq as Compared to Neighbors of         C14 Check
PLAT244_ALERT_4_C Low    'Solvent' Ueq as Compared to Neighbors of         C1R Check
PLAT260_ALERT_2_C Large Average Ueq of Residue Including      Cl1S      0.116 Check
```

Alert level G

```
PLAT083_ALERT_2_G SHELXL Second Parameter in WGHT  Unusually Large      11.40 Why ?
PLAT143_ALERT_4_G s.u. on c - Axis Small or Missing .............    0.00020 Ang.
PLAT432_ALERT_2_G Short Inter X...Y Contact  F10       ..C3       .       2.97 Ang.
                                     1/2-x,1/2+y,1/2-z  =      2_555 Check
PLAT434_ALERT_2_G Short Inter HL..HL Contact F7       ..F13      .       2.67 Ang.
                                    -1/2+x,1/2-y,1/2+z  =      4_566 Check
PLAT720_ALERT_4_G Number of Unusual/Non-Standard Labels .........          4 Note
PLAT899_ALERT_4_G SHELXL2018 is Deprecated and Succeeded by SHELXL     2019/3 Note
```

0 **ALERT level A** = Most likely a serious problem - resolve or explain
0 **ALERT level B** = A potentially serious problem, consider carefully
5 **ALERT level C** = Check. Ensure it is not caused by an omission or oversight
6 **ALERT level G** = General information/check it is not something unexpected

0 ALERT type 1 CIF construction/syntax error, inconsistent or missing data
7 ALERT type 2 Indicator that the structure model may be wrong or deficient
0 ALERT type 3 Indicator that the structure quality may be low
4 ALERT type 4 Improvement, methodology, query or suggestion
0 ALERT type 5 Informative message, check

Datablock: ESY179

Bond precision:	C-C = 0.0103 A		Wavelength=1.54184
Cell:	a=8.9844(1)	b=8.9844(1)	c=22.4971(6)
	alpha=90	beta=90	gamma=90
Temperature:	100 K		

	Calculated	Reported
Volume	1815.95(6)	1815.95(6)
Space group	P 43 21 2	P 43 21 2
Hall group	P 4nw 2abw	P 4nw 2abw
Moiety formula	C5 H9 I N3 P	C5 H9 I N3 P
Sum formula	C5 H9 I N3 P	C5 H9 I N3 P
Mr	269.02	269.02
Dx,g cm-3	1.968	1.968
Z	8	8
Mu (mm-1)	28.879	28.879
F000	1024.0	1024.0
F000'	1026.76	
h,k,lmax	11,11,28	11,11,28
Nref	1872[1159]	1866
Tmin,Tmax	0.014,0.023	0.051,0.272
Tmin'	0.000	

Correction method= # Reported T Limits: Tmin=0.051 Tmax=0.272
AbsCorr = GAUSSIAN

Data completeness= 1.61/1.00 Theta(max)= 74.621

R(reflections)= 0.0523(1594) wR2(reflections)= 0.1533(1866)

S = 1.067 Npar= 95

The following ALERTS were generated. Each ALERT has the format
test-name_ALERT_alert-type_alert-level.
Click on the hyperlinks for more details of the test.

Alert level C

```
RINTA01_ALERT_3_C  The value of Rint is greater than 0.12
            Rint given   0.125
PLAT342_ALERT_3_C Low Bond Precision on  C-C Bonds ..............    0.01033 Ang.
```

Alert level G

```
PLAT020_ALERT_3_G The Value of Rint is Greater Than 0.12 .........      0.125 Report
PLAT431_ALERT_2_G Short Inter HL..A Contact  I1       ..N2       .       3.27 Ang.
                                                 y,1+x,1-z  =      7_566 Check
PLAT899_ALERT_4_G SHELXL2018 is Deprecated and Succeeded by SHELXL     2019/3 Note
```

---

0 **ALERT level A** = Most likely a serious problem - resolve or explain
0 **ALERT level B** = A potentially serious problem, consider carefully
2 **ALERT level C** = Check. Ensure it is not caused by an omission or oversight
3 **ALERT level G** = General information/check it is not something unexpected

```
   0 ALERT type 1 CIF construction/syntax error, inconsistent or missing data
   1 ALERT type 2 Indicator that the structure model may be wrong or deficient
   3 ALERT type 3 Indicator that the structure quality may be low
   1 ALERT type 4 Improvement, methodology, query or suggestion
   0 ALERT type 5 Informative message, check
```

Datablock: ESY503

Bond precision: C-C = 0.0041 A   Wavelength=1.54184

Cell:	a=13.2814(4)	b=6.9637(2)	c=13.6715(3)
	alpha=90	beta=96.888(2)	gamma=90
Temperature:	150 K		

	Calculated	Reported
Volume	1255.32(6)	1255.32(6)
Space group	P 21/c	P 21/c
Hall group	-P 2ybc	-P 2ybc
Moiety formula	C11 H15 I N3 P	C11 H15 I N3 P
Sum formula	C11 H15 I N3 P	C11 H15 I N3 P
Mr	347.13	347.13
Dx,g cm-3	1.837	1.837
Z	4	4
Mu (mm-1)	21.062	21.062
F000	680.0	680.0
F000'	681.59	
h,k,lmax	16,8,17	16,8,17
Nref	2630	2600
Tmin,Tmax	0.418,0.656	0.431,1.000
Tmin'	0.106	

Correction method= # Reported T Limits: Tmin=0.431 Tmax=1.000
AbsCorr = MULTI-SCAN

Data completeness= 0.989   Theta(max)= 76.241

R(reflections)= 0.0235(2108)   wR2(reflections)= 0.0582(2600)

S = 1.061   Npar= 145

The following ALERTS were generated. Each ALERT has the format
test-name_ALERT_alert-type_alert-level.
Click on the hyperlinks for more details of the test.

Alert level G

```
PLAT899_ALERT_4_G SHELXL2018 is Deprecated and Succeeded by SHELXL     2019/3 Note
PLAT941_ALERT_3_G Average HKL Measurement Multiplicity ..........        3.3 Low
```

```
   0 ALERT level A = Most likely a serious problem - resolve or explain
   0 ALERT level B = A potentially serious problem, consider carefully
   0 ALERT level C = Check. Ensure it is not caused by an omission or oversight
   2 ALERT level G = General information/check it is not something unexpected

   0 ALERT type 1 CIF construction/syntax error, inconsistent or missing data
   0 ALERT type 2 Indicator that the structure model may be wrong or deficient
   1 ALERT type 3 Indicator that the structure quality may be low
   1 ALERT type 4 Improvement, methodology, query or suggestion
   0 ALERT type 5 Informative message, check
```

Validation response form

Please find below a validation response form (VRF) that can be filled in and pasted into your CIF.

```
# start Validation Reply Form
_vrf_PLAT220_ESY100
;
PROBLEM: NonSolvent   Resd 1  C   Ueq(max)/Ueq(min) Range        6.2 Ratio
RESPONSE: ...
;
_vrf_PLAT242_ESY100
;
PROBLEM: Low    'MainMol' Ueq as Compared to Neighbors of        C29 Check
RESPONSE: ...
;
_vrf_PLAT330_ESY100
;
PROBLEM: Large Aver Phenyl C-C Dist C1S      --C6S      .       1.43 Ang.
RESPONSE: ...
;
_vrf_PLAT250_ESY506
;
PROBLEM: Large U3/U1 Ratio for Average U(i,j) Tensor ....        4.4 Note
RESPONSE: ...
;
_vrf_PLAT220_AM010
;
PROBLEM: NonSolvent   Resd 1  C   Ueq(max)/Ueq(min) Range        7.0 Ratio
RESPONSE: ...
;
_vrf_PLAT242_AM010
;
PROBLEM: Low    'MainMol' Ueq as Compared to Neighbors of        C38 Check
RESPONSE: ...
;
_vrf_PLAT213_AM013B
;
PROBLEM: Atom C59             has ADP max/min Ratio .....        4.6 prolat
RESPONSE: ...
```

```
;
_vrf_PLAT220_AM013B
;
PROBLEM: NonSolvent   Resd 1  C   Ueq(max)/Ueq(min) Range        7.1 Ratio
RESPONSE: ...
;
_vrf_PLAT242_AM013B
;
PROBLEM: Low    'MainMol' Ueq as Compared to Neighbors of        C30 Check
RESPONSE: ...
;
_vrf_PLAT250_AM013B
;
PROBLEM: Large U3/U1 Ratio for Average U(i,j) Tensor ....        5.7 Note
RESPONSE: ...
;
_vrf_PLAT110_ESY153
;
PROBLEM: ADDSYM Detects Potential Lattice Translation ...          ? Check
RESPONSE: ...
;
_vrf_PLAT112_ESY153
;
PROBLEM: ADDSYM Detects New (Pseudo) Symm. Elem         I        100 %Fit
RESPONSE: ...
;
_vrf_PLAT113_ESY153
;
PROBLEM: ADDSYM Suggests Possible Pseudo/New  Space Group       I4/m Check
RESPONSE: ...
;
_vrf_PLAT601_ESY153
;
PROBLEM: Unit Cell Contains Solvent Accessible VOIDS of .        105 Ang**3
RESPONSE: ...
;
_vrf_PLAT220_ESY338
;
PROBLEM: NonSolvent   Resd 1  C   Ueq(max)/Ueq(min) Range        7.5 Ratio
RESPONSE: ...
;
_vrf_PLAT242_ESY338
;
PROBLEM: Low    'MainMol' Ueq as Compared to Neighbors of        C11 Check
RESPONSE: ...
;
# end Validation Reply Form
```

It is advisable to attempt to resolve as many as possible of the alerts in all categories. Often the minor alerts point to easily fixed oversights, errors and omissions in your CIF or refinement strategy, so attention to these fine details can be worthwhile. In order to resolve some of the more serious problems it may be necessary to carry out additional measurements or structure refinements. However, the purpose of your study may justify the reported deviations and the more serious of these should normally be commented upon in the discussion or experimental section of a paper or in the "special_details" fields of the CIF. checkCIF was carefully designed to identify outliers and unusual parameters, but every test has its limitations and alerts that are not important in a particular case may appear. Conversely, the absence of alerts does not guarantee there are no aspects of the results needing attention. It is up to the individual to critically assess their own results and, if necessary, seek expert advice.

Publication of your CIF in IUCr journals

A basic structural check has been run on your CIF. These basic checks will be run on all CIFs submitted for publication in IUCr journals (*Acta Crystallographica*, *Journal of Applied Crystallography*, *Journal of Synchrotron Radiation*); however, if you intend to submit to *Acta Crystallographica Section C* or *E* or *IUCrData*, you should make sure that full publication checks are run on the final version of your CIF prior to submission.

Publication of your CIF in other journals

Please refer to the *Notes for Authors* of the relevant journal for any special instructions relating to CIF submission.

PLATON version of 10/05/2023; check.def file version of 10/05/2023

Datablock ESY100 - ellipsoid plot

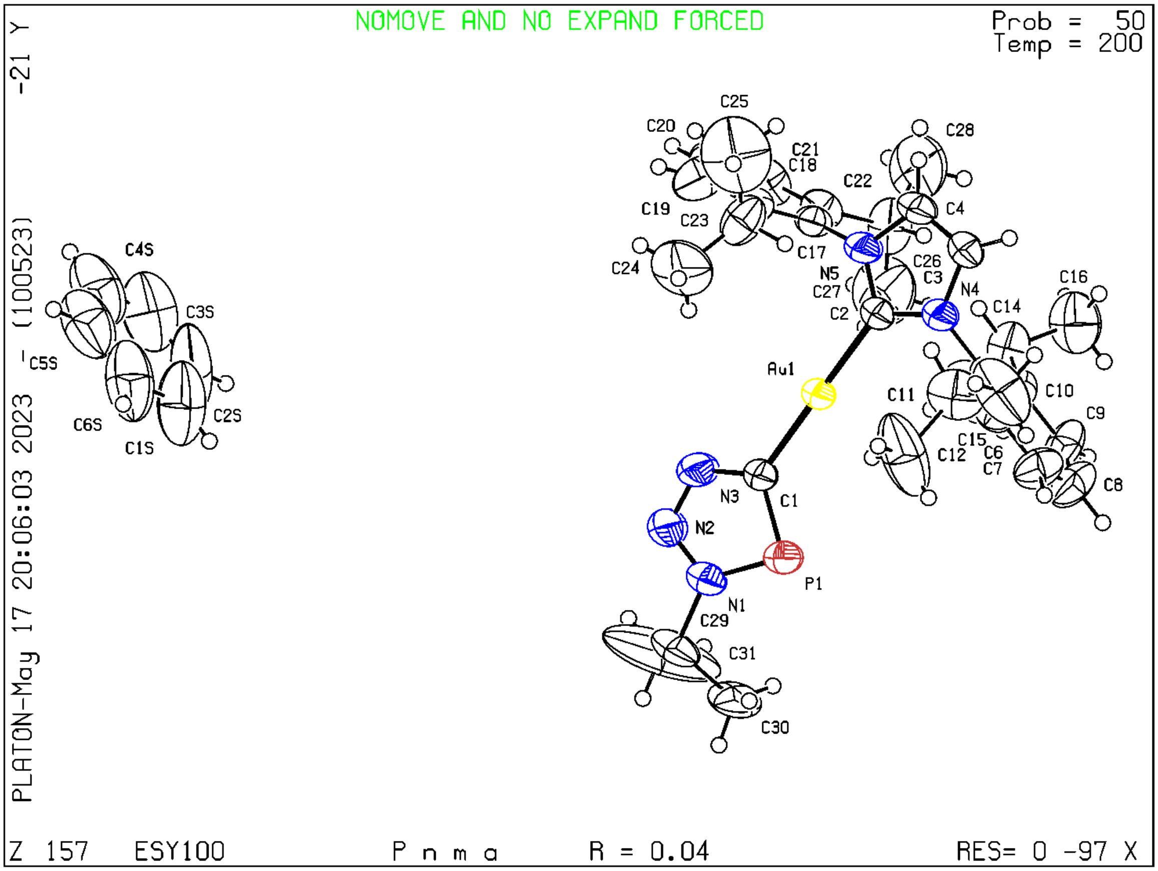

Datablock ESY506 - ellipsoid plot

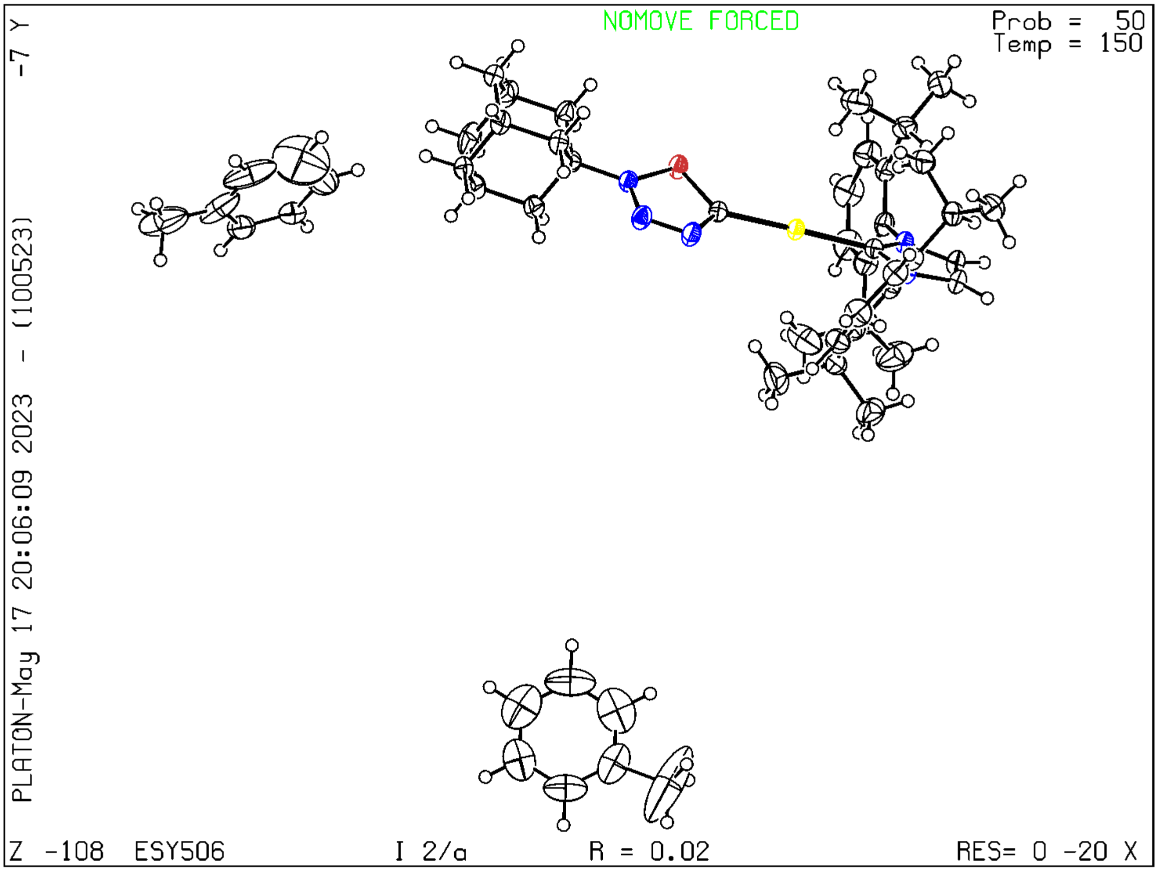

Datablock AM010 - ellipsoid plot

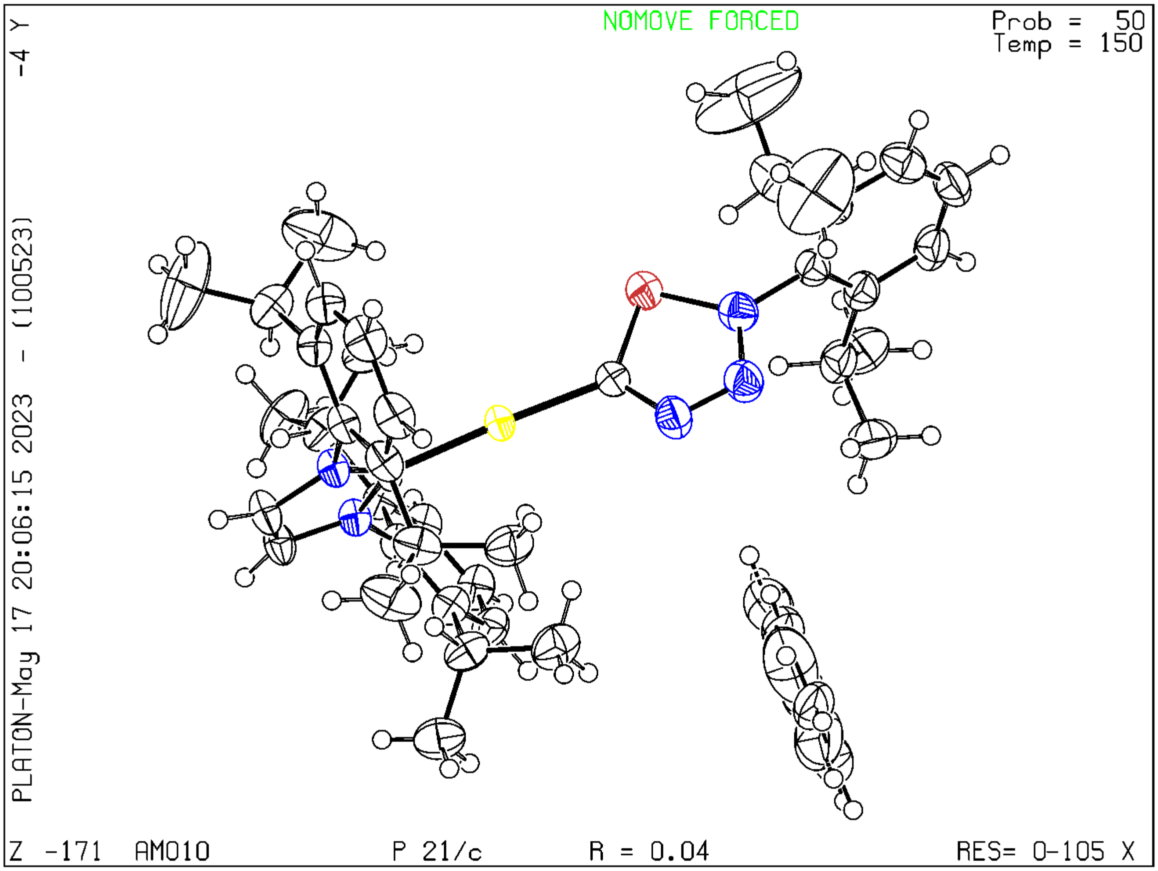

Datablock AM013B - ellipsoid plot

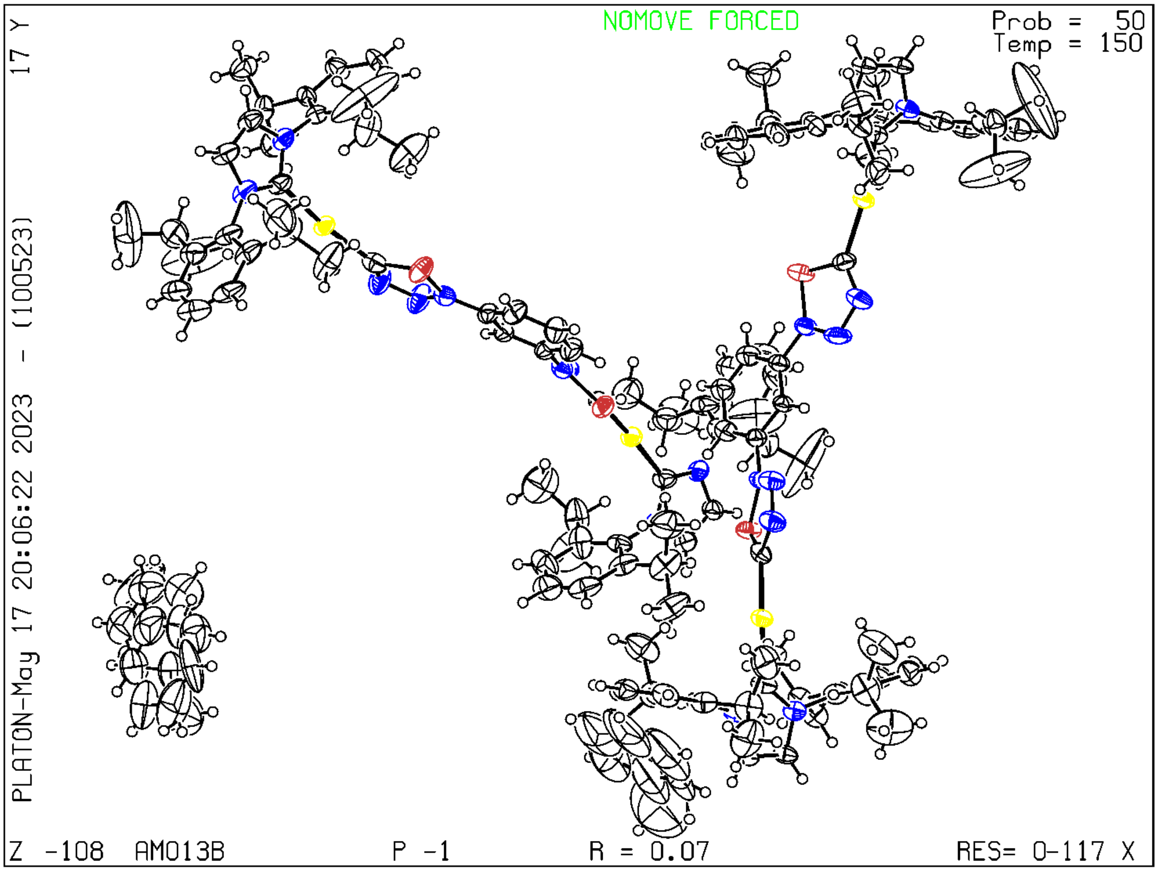

Datablock ESY026 - ellipsoid plot

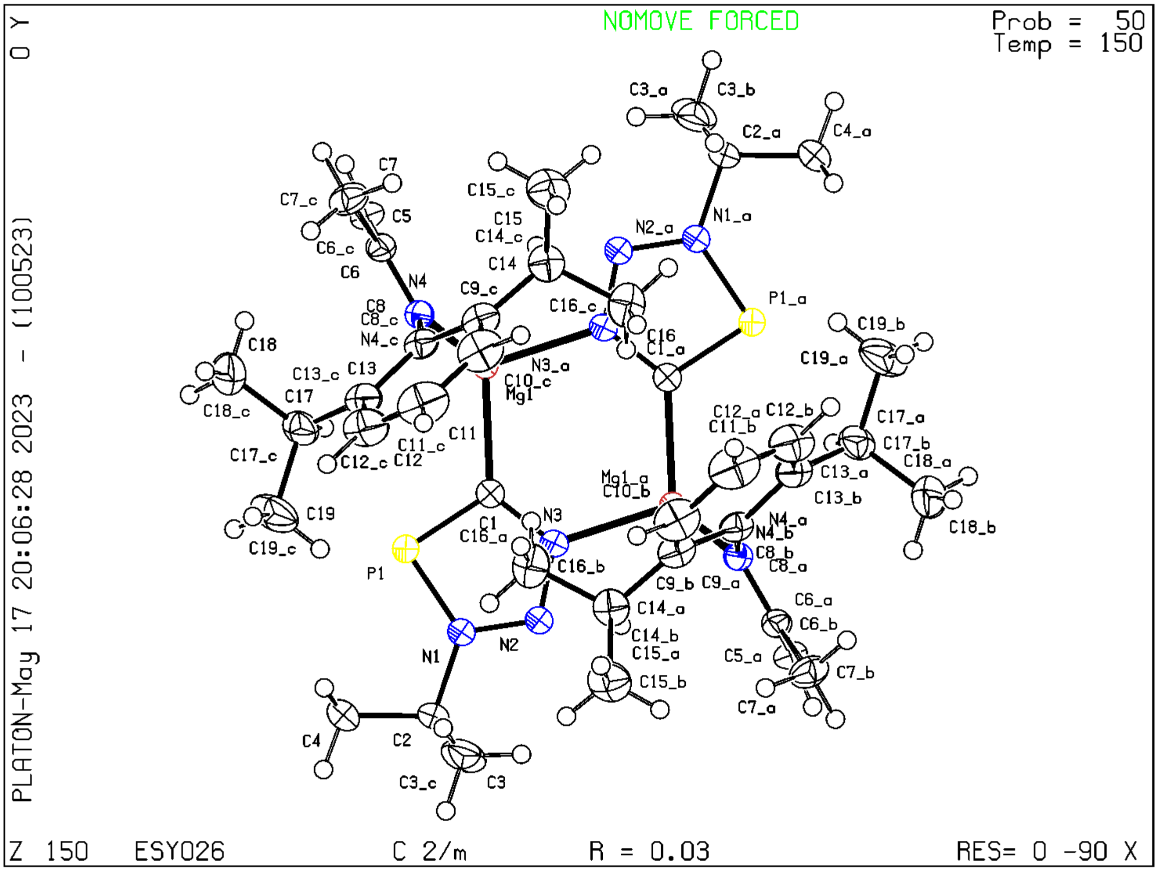

Datablock ESY064 - ellipsoid plot

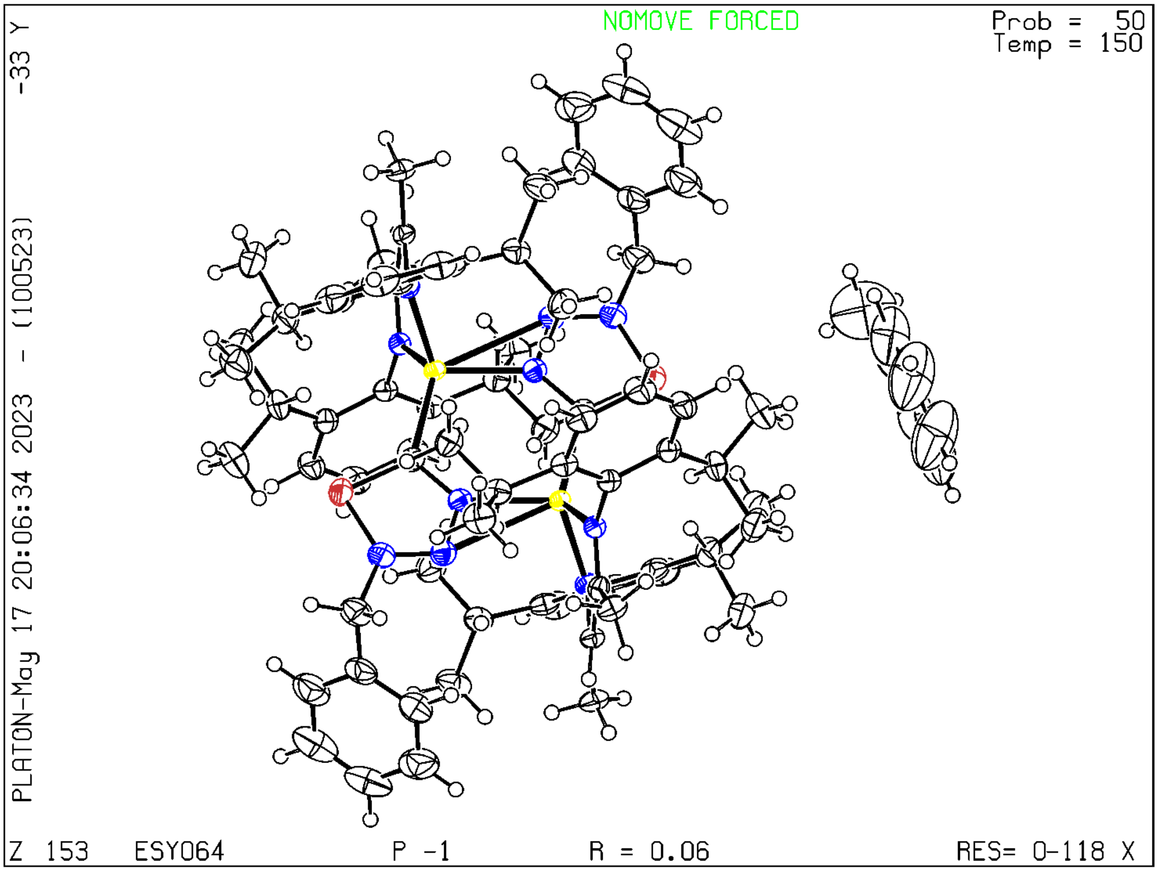

Datablock ESY153 - ellipsoid plot

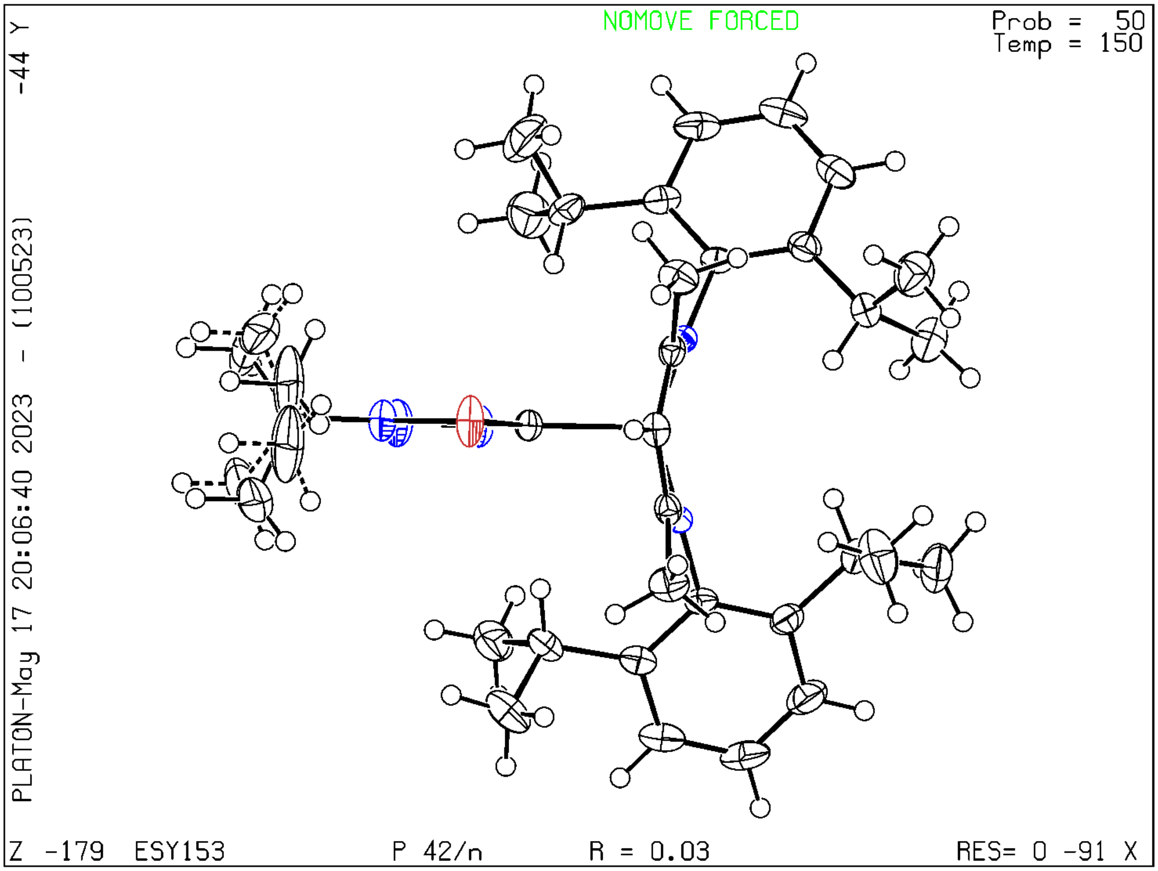

Datablock ESY338 - ellipsoid plot

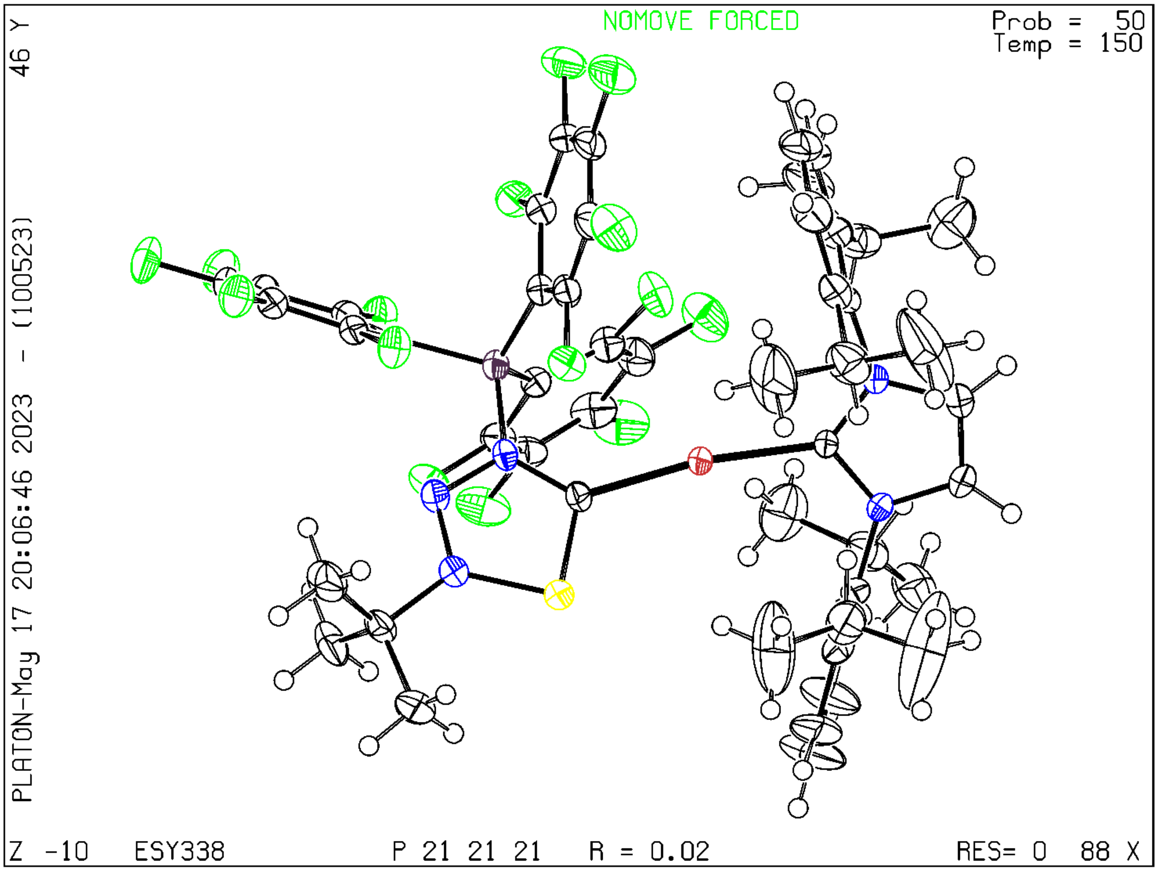

Datablock AM026 - ellipsoid plot

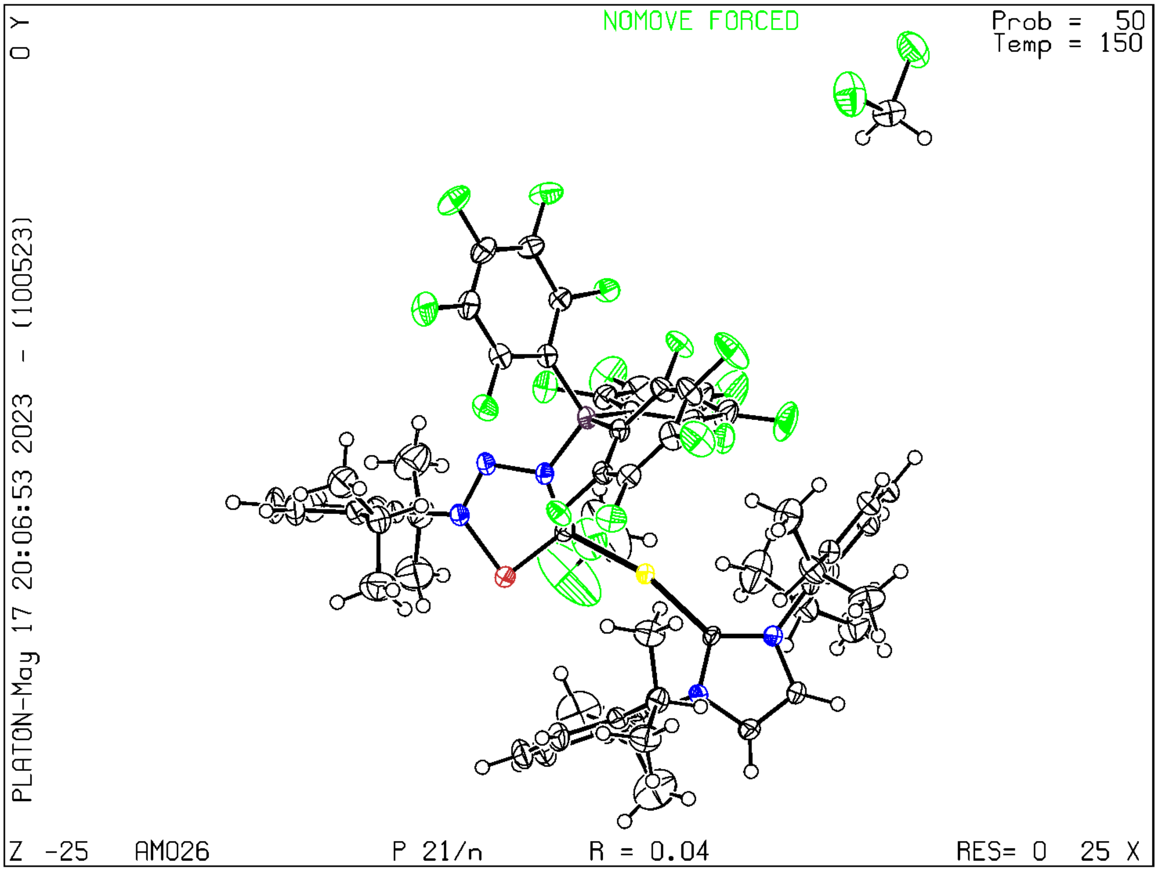

Datablock ESY179 - ellipsoid plot

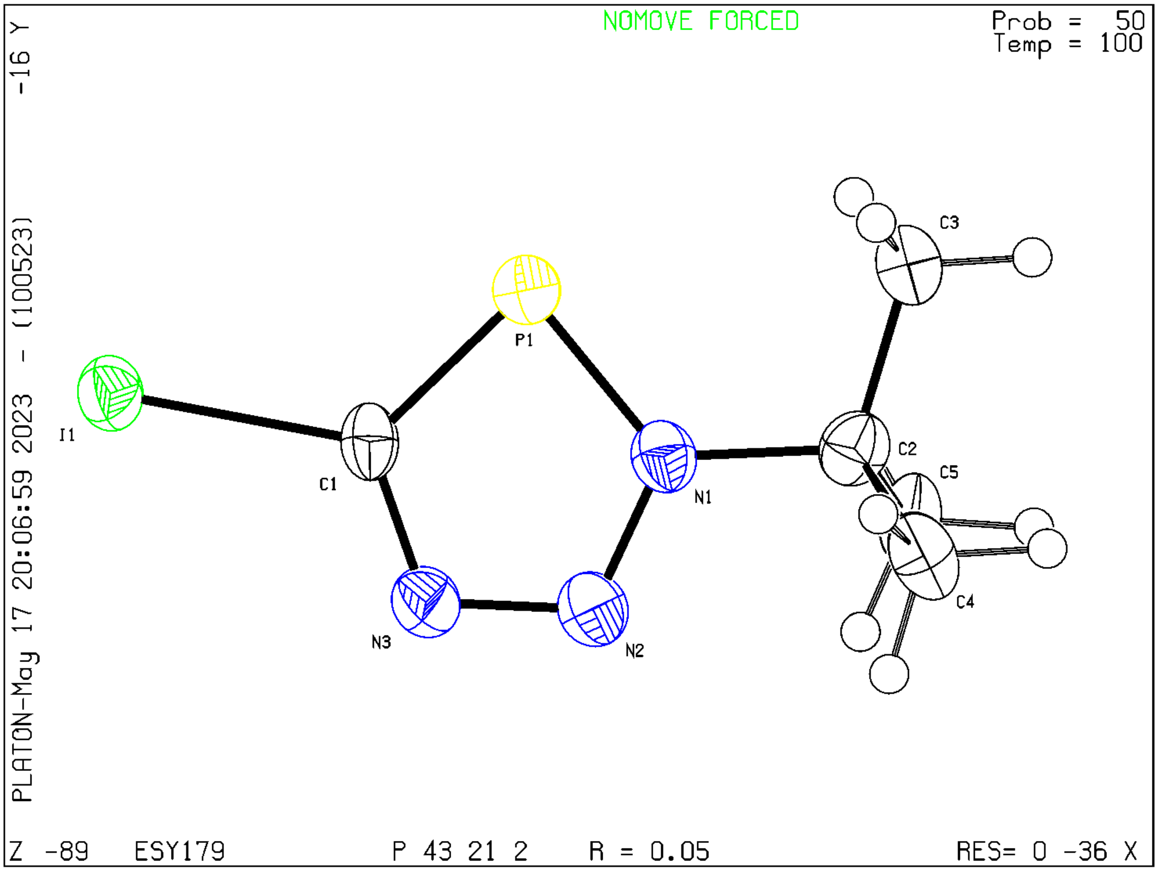

Datablock ESY503 - ellipsoid plot

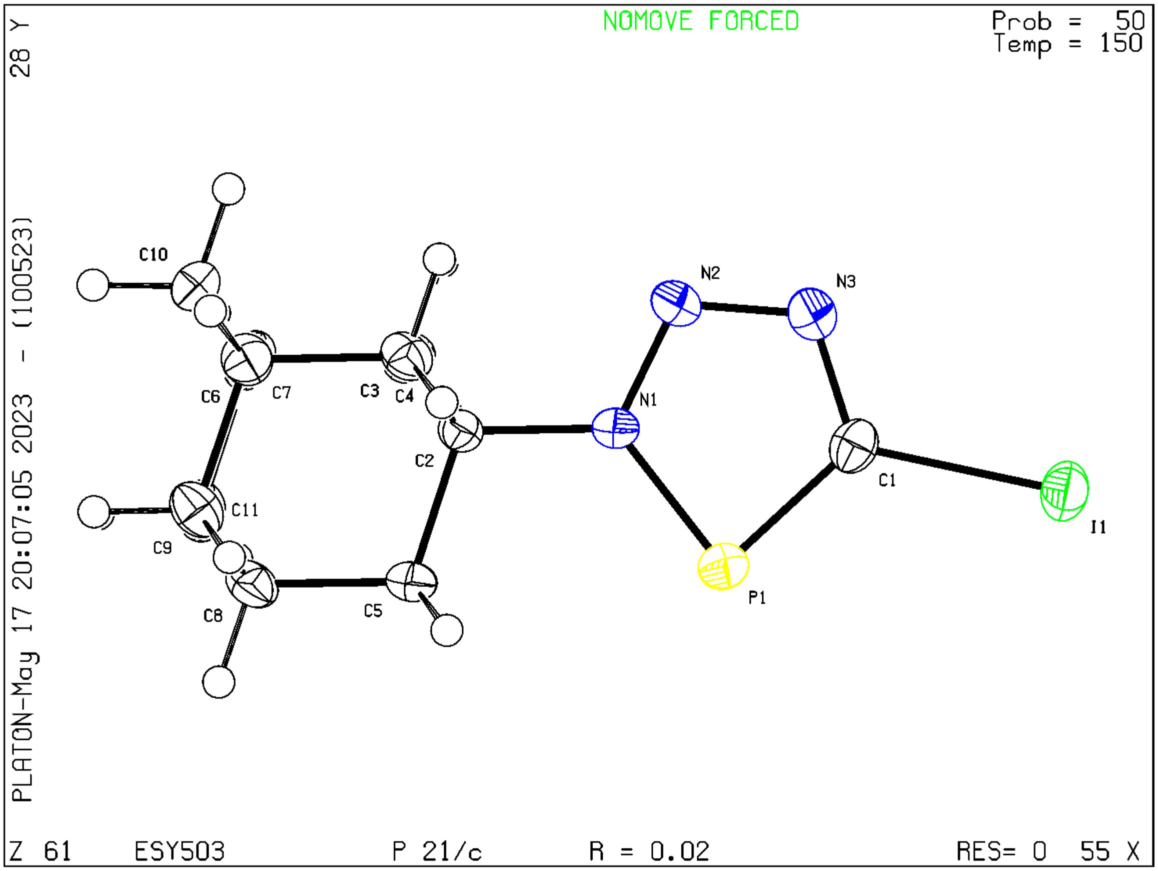

Supplement: Supplementary file 1 — Supporting Information [file CHEM-29-0-s001.pdf]
