# Supplementary material for: Cyaphide‐Azide 1,3‐Dipolar Cycloaddition Reactions: Scope and Applicability
Source: Chemistry. 2023 Aug 10;29(52):e202301648. doi: 10.1002/chem.202301648 (PMC10946888; doi:10.1002/chem.202301648)
Supplement: Supplementary file 2 — Supporting Information [file CHEM-29-0-s002.pdf]

Chemistry–A European Journal

Supporting Information

Cyaphide-Azide 1,3-Dipolar Cycloaddition Reactions: Scope and Applicability

Eric S. Yang, Alex Mapp, Andrew Taylor, Paul D. Beer,* and Jose M. Goicoechea*

Contents

1. Experimental section 2

1.1 General experimental methods 2

1.2 Synthesis of reported compounds 3

2. Single crystal X-ray diffraction data 35

3. Computational details 42

3.1. General computational methods 42

3.2. ICPN$_3$R 43

3.5. XYZ coordinates 44

4. References 45

1. Experimental section

1.1 General experimental methods

Synthetic methods. All reactions and product manipulations were carried out using standard Schlenk-line techniques under an inert atmosphere of argon, or in a dinitrogen filled glovebox (MBraun UNIlab glovebox maintained at < 0.1 ppm H_2O and < 0.1 ppm O_2). *Tert*-butyl azide,[33] 1,3-diisopropylphenyl azide,[34] 1,3-diazidobenzene,[35] Au(IDipp)(CP),[19] and Ge(^{Dipp}NacNac)Cl[36] were synthesized according to previously reported synthetic procedures. Mg(^{Dipp}NacNac)(diox.)(CP) was generated *in situ* according to a previously reported procedure.[19] Adamantyl azide (Sigma Aldrich, 97%), pyridinium chloride (Sigma Aldrich, 98%) and iodine (Sigma Aldrich, 99.8%) were purchased and used as received. Benzyl azide (Alfa Aesar, 94%) was distilled and dried over 3 Å molecular sieves. Toluene (Sigma Aldrich, HPLC grade), hexane (Sigma Aldrich, HPLC grade), and pentane (Sigma Aldrich, HPLC grade) were purified using an MBraun SPS-800 solvent system. THF (Sigma Aldrich, HPLC grade) was distilled over sodium/benzophenone. 1,2-DFB (Alfa Aesar, 98%) was distilled over CaH_2. C_6D_6 (Aldrich, 99.5%) and CD_2Cl_2 (Aldrich, 99.5%) were degassed and dried over CaH_2. d_8-toluene (Aldrich, 99.5%) was degassed and dried over Na/K alloy. All dry solvents were stored under argon in gas-tight ampoules over activated 3 Å molecular sieves.

Characterization techniques. NMR spectra were acquired on a Bruker AVIII 400 MHz NMR spectrometer (^{1}H 400 MHz, ^{31}P 162 MHz, ^{19}F 377 MHz, ^{11}B 128 MHz), Bruker AVIII 500 MHz NMR spectrometer (^{1}H 500 MHz, ^{13}C 126 MHz) or a Bruker Avance NEO 600 MHz NMR spectrometer with a broadband helium cryoprobe (^{1}H 600 MHz, ^{13}C 151 MHz). ^{1}H and ^{13}C NMR spectra were referenced to the most downfield solvent resonance (^{1}H NMR C_6D_6: δ = 7.16 ppm, ^{13}C NMR C_6D_6: δ = 128.06 ppm; ^{1}H NMR CD_2Cl_2: δ = 5.32 ppm, ^{13}C NMR CD_2Cl_2: δ = 53.84 ppm). ^{31}P NMR spectra were externally referenced to an 85% solution of H_3PO_4 in H_2O. ^{11}B NMR spectra were externally referenced to $BF_3 \cdot Et_2O$ in C_6D_6. ^{19}F NMR spectra were externally referenced to $CFCl_3$. Elemental analyses were carried out by Elemental Microanalyses Ltd. (Devon, U.K.) or by London Metropolitan University (London, U.K.). Samples (approx. 5 mg) were submitted in flame sealed glass tubes.

1.2 Synthesis of reported compounds

1.2.1 Synthesis of Au(IDipp)(CPN$_3$^{t}Bu) (1a)

Neat *tert*-butyl azide (8 mg, 0.08 mmol) was added to a solution of Au(IDipp)(CP) (50 mg, 0.08 mmol) in toluene (1 mL) and stirred at room temperature for 2 h. The solution was concentrated by slow evaporation, yielding colorless crystals suitable for single crystal X-ray crystallography. The mixture was then stored at −35 ºC for 3 days to yield colorless crystals which were isolated by filtration. Yield: 50 mg, 0.07 mmol, 86%. Anal. Calcd. (%) for $C_{32}H_{45}AuN_5P$: C, 52.82; H, 6.23; N, 9.62. Found: C, 52.49; H, 6.31; N, 9.00.

^{1}H NMR (600 MHz, C_6D_6): δ(ppm) 7.18 (t, $^3J_{H\text{–}H}$ = 7.8 Hz, 2H, Dipp *para* C**H**), 7.06 (d, $^3J_{H\text{–}H}$ = 7.8 Hz, 4H, Dipp *meta* C**H**), 6.35 (s, 2H, IDipp C**H**), 2.69 (sept, $^3J_{H\text{–}H}$ = 6.9 Hz, 4H, Dipp C**H**(CH_3)$_2$), 1.53 (d, $^3J_{H\text{–}H}$ = 6.9 Hz, 12H, Dipp CH(C**H**$_3$)$_2$), 1.42 (s, 9H, ^{t}Bu C(C**H**$_3$)$_3$), 1.10 (d, $^3J_{H\text{–}H}$ = 6.9 Hz, 12H, Dipp CH(C**H**$_3$)$_2$).

^{13}C{^{1}H} NMR (151 MHz, C_6D_6): δ(ppm) 205.19 (d, $^1J_{C\text{–}P}$ = 82.0 Hz, **C**PN$_3$^{t}Bu), 196.51 (d, $^3J_{C\text{–}P}$ = 11.1 Hz, IDipp {HCN(Dipp)}$_2$**C**Au), 145.90 (Dipp *ortho* **C**), 134.80 (Dipp *ipso* **C**), 130.68 (Dipp *para* **C**), 124.26 (Dipp *meta* **C**), 122.73 (IDipp **C**H), 59.69 (d, $^2J_{C\text{–}P}$ = 5.1 Hz, ^{t}Bu **C**(CH_3)$_3$), 32.03 (d, $^3J_{C\text{–}P}$ = 5.0 Hz, ^{t}Bu C(**C**H$_3$)$_3$), 29.14 (Dipp **C**H(CH_3)$_2$), 24.93 (Dipp CH(**C**H$_3$)$_2$), 23.98 (Dipp CH(**C**H$_3$)$_2$).

^{31}P{^{1}H} NMR (162 MHz, C_6D_6): δ(ppm) 191.2 (s, C**P**N$_3$^{t}Bu).

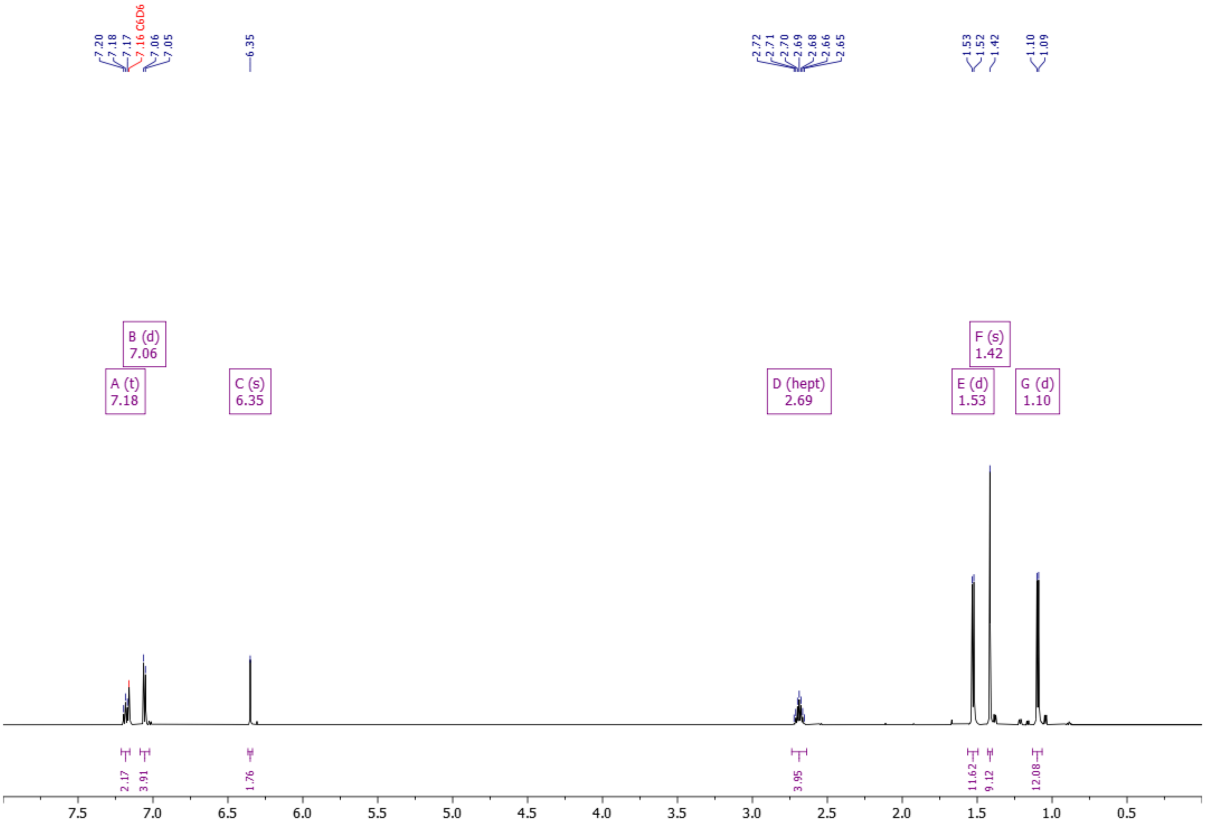

Figure S1. ^{1}H NMR (600 MHz) spectrum of **1a** in C_6D_6.

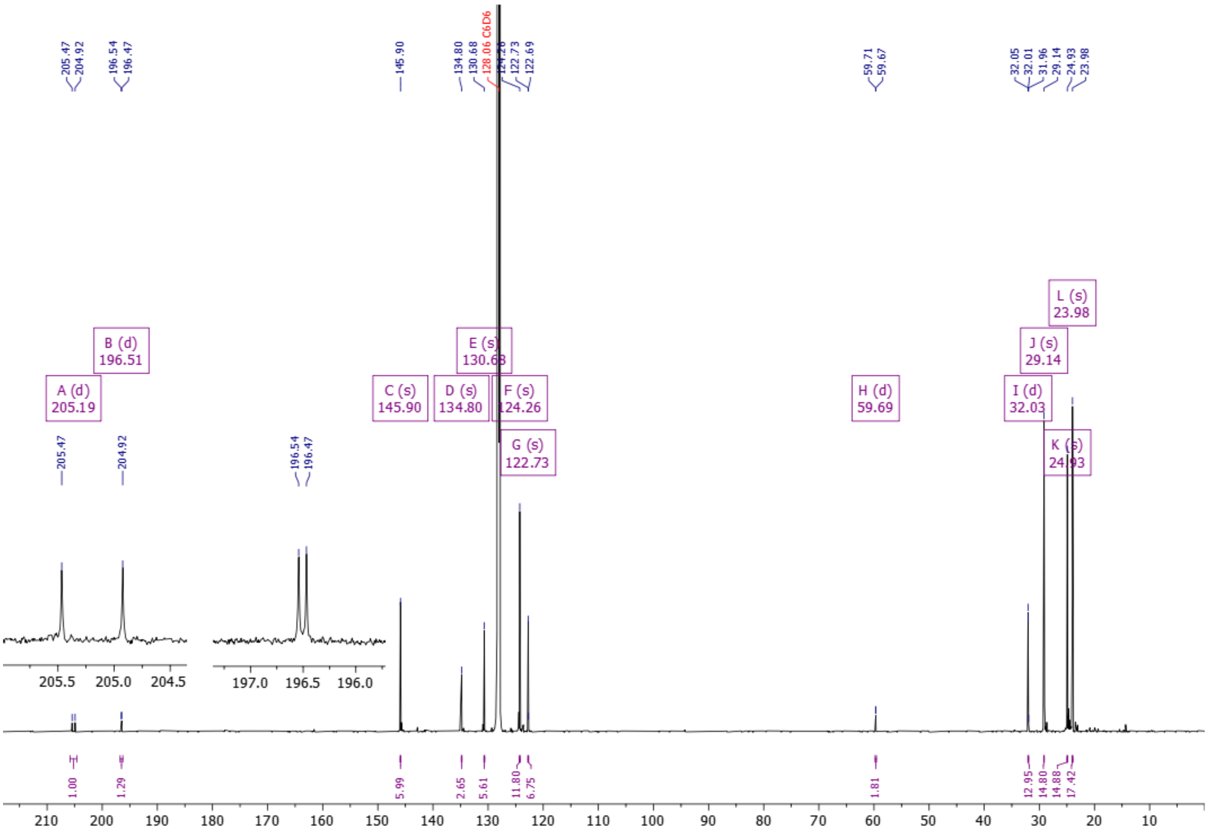

Figure S2. $^{13}C\{^{1}H\}$ NMR (151 MHz) spectrum of **1a** in C_6D_6.

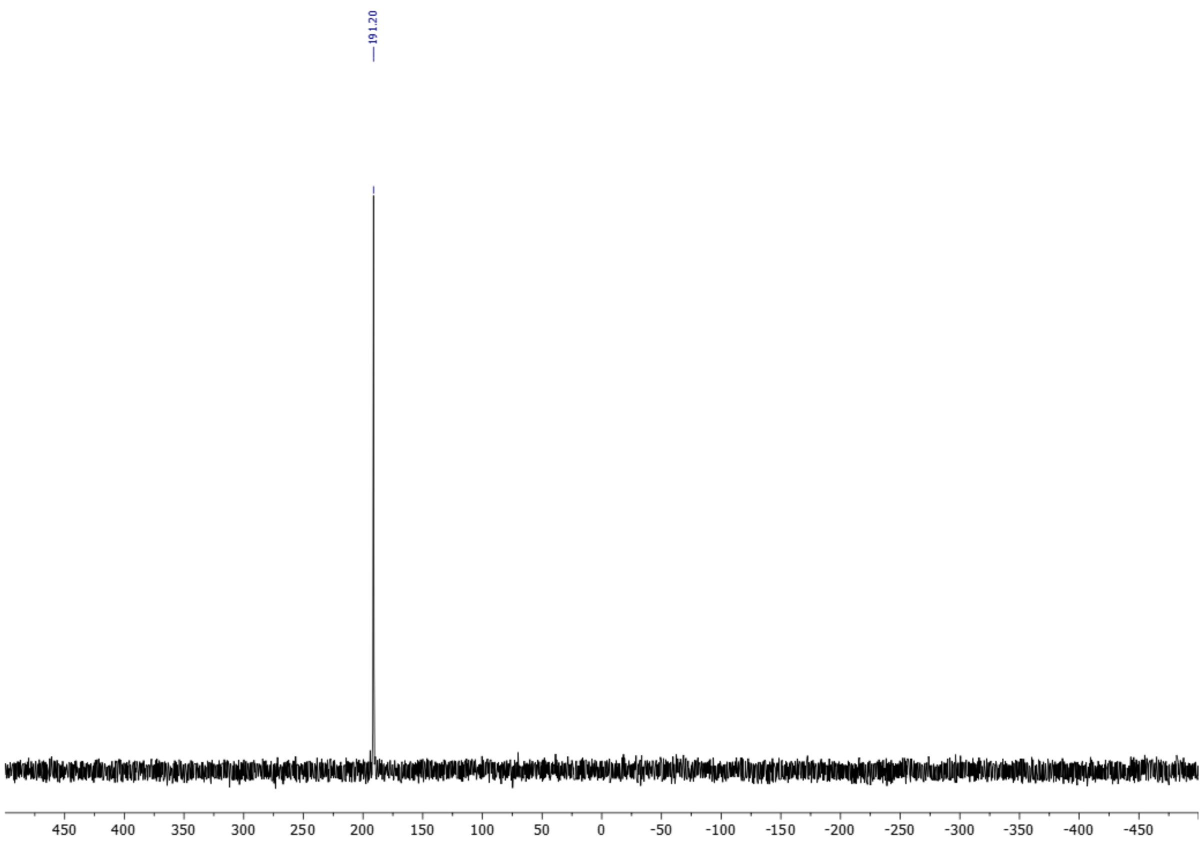

Figure S3. $^{31}P\{^{1}H\}$ NMR (162 MHz) spectrum of **1a** in C_6D_6.

1.2.2 Synthesis of Au(IDipp)(CPN$_3$Ad) (1b)

1-adamantyl azide (20 mg, 0.11 mmol) was added to a solution of Au(IDipp)(CP) (71 mg, 0.11 mmol) in toluene (1 mL) and stirred at room temperature overnight. The solution was concentrated under vacuum, then stored at −35 ºC for 3 days to yield colorless crystals which were isolated by filtration. Yield: 82 mg, 0.10 mmol, 90%. Anal. Calcd. (%) for $C_{38}H_{51}AuN_5P$: C, 56.64; H, 6.38; N, 8.69. Found: C, 57.05; H, 6.17; N, 8.43.

^{1}H NMR (600 MHz, C_6D_6): δ(ppm) 7.20 (t, $^{3}J_{H\text{–}H}$ = 7.8 Hz, 2H, Dipp *para* C**H**), 7.07 (d, $^{3}J_{H\text{–}H}$ = 7.8 Hz, 4H, Dipp *meta* C**H**), 6.39 (s, 2H, IDipp C**H**), 2.70 (sept, $^{3}J_{H\text{–}H}$ = 6.9 Hz, 4H, Dipp C**H**(CH$_3$)$_2$), 2.17 (d, $^{3}J_{H\text{–}H}$ = 2.8 Hz, 6H, Ad C{C**H**$_2$CHCH$_2$}$_3$), 1.85–1.77 (m, 3H, Ad {CH$_2$C**H**CH$_2$}$_3$), 1.54 (d, $^{3}J_{H\text{–}H}$ = 6.9 Hz, 12H, Dipp CH(C**H**$_3$)$_2$), 1.45–1.37 (m, 6H, Ad {CH$_2$CHC**H**$_2$}$_3$), 1.11 (d, $^{3}J_{H\text{–}H}$ = 6.9 Hz, 12H, Dipp CH(C**H**$_3$)$_2$).

$^{13}C\{^{1}H\}$ NMR (151 MHz, C_6D_6): δ(ppm) 204.52 (d, $^{1}J_{C\text{–}P}$ = 81.9 Hz, *C*PN$_3$Ad), 196.54 (d, $^{3}J_{C\text{–}P}$ = 11.0 Hz, IDipp {HCN(Dipp)}$_2$**C**Au), 145.89 (Dipp *ortho* **C**), 134.82 (Dipp *ipso* **C**), 130.70 (Dipp *para* **C**), 124.27 (Dipp *meta* **C**), 122.78 (IDipp **C**H), 60.19 (d, $^{2}J_{C\text{–}P}$ = 4.4 Hz, Ad **C**{CH$_2$CHCH$_2$}$_3$), 45.76 (d, $^{3}J_{C\text{–}P}$ = 5.1 Hz, Ad C{**C**H$_2$CHCH$_2$}$_3$), 36.54 (Ad C{CH$_2$CH**C**H$_2$}$_3$),

30.28 (Ad C{CH_2**C**HCH_2}$_3$), 29.15 (Dipp **C**H(CH_3)$_2$), 24.94 (Dipp CH(**C**H_3)$_2$), 24.01 (Dipp CH(**C**H_3)$_2$).

$^{31}P\{^1H\}$ NMR (162 MHz, C_6D_6): δ(ppm) 189.3 ppm (s, C**P**N_3Ad).

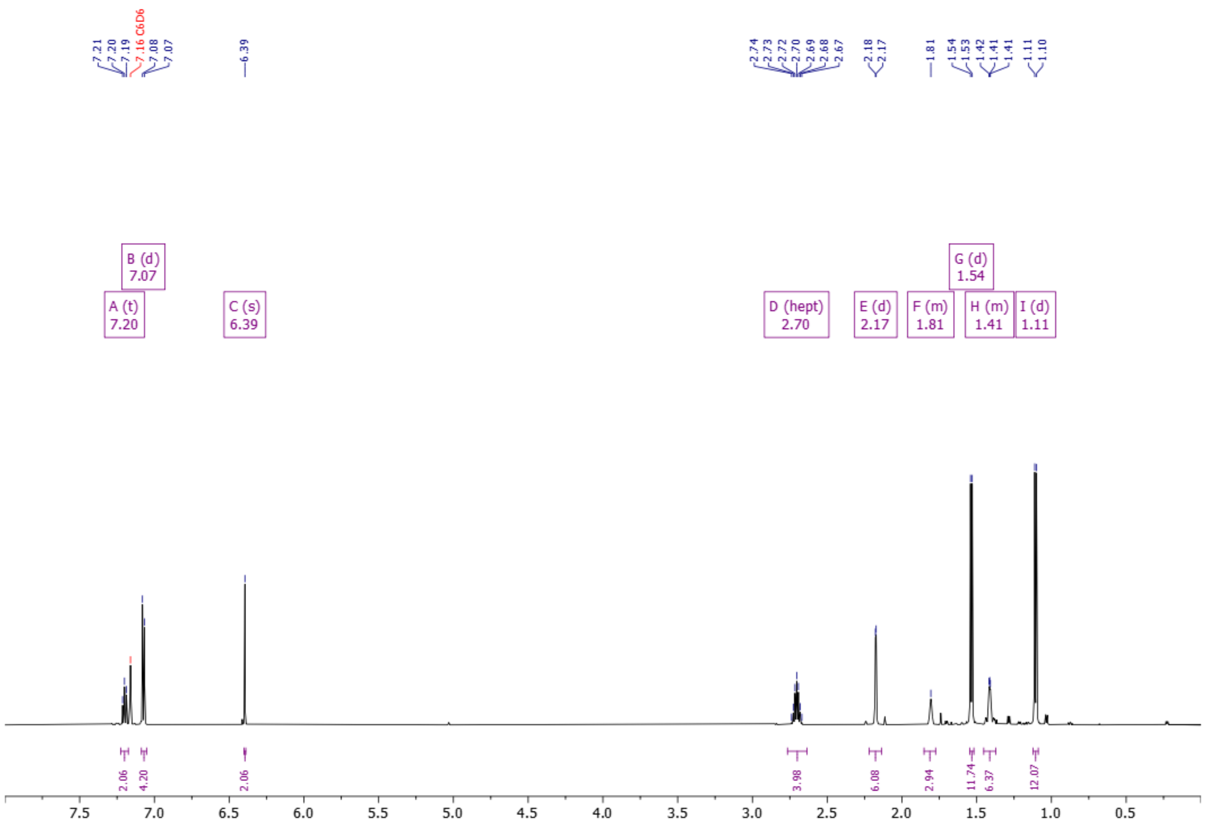

Figure S4. ^1H NMR (600 MHz) spectrum of **1b** in C_6D_6.

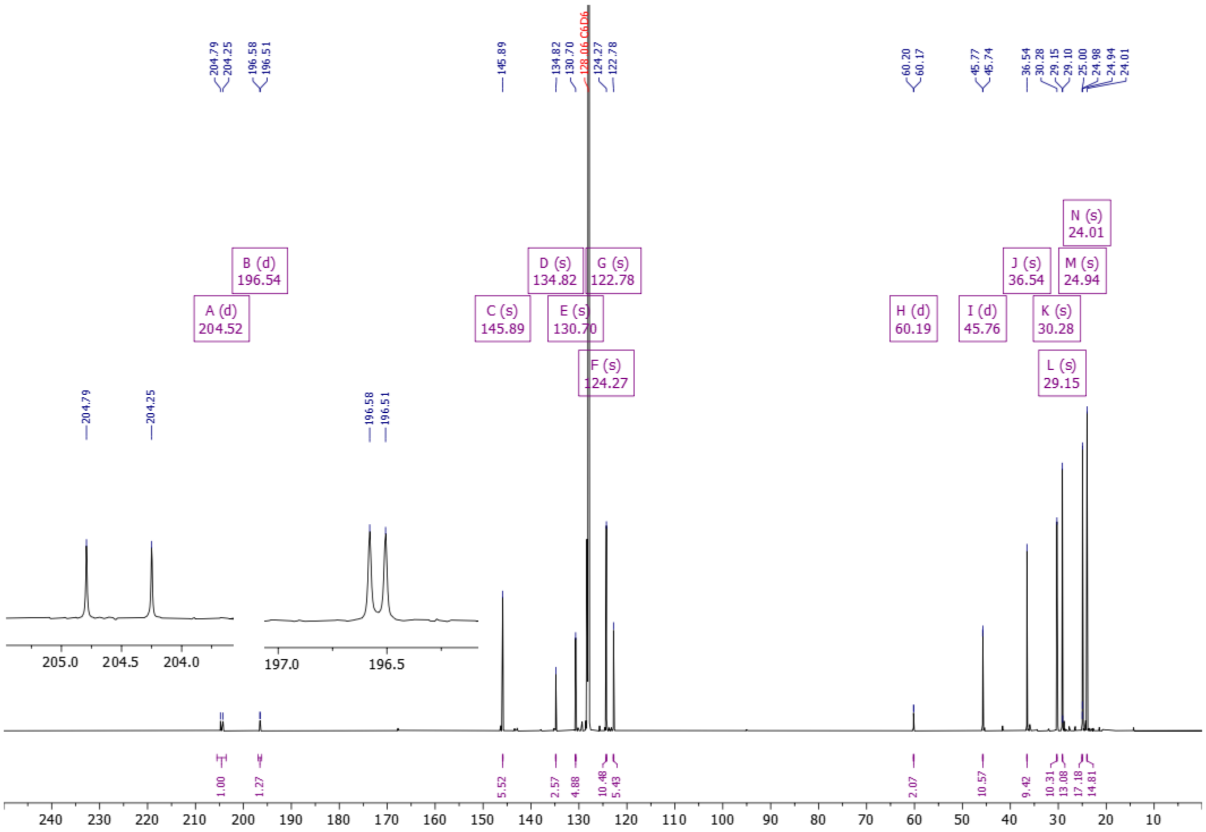

Figure S5. $^{13}C\{^{1}H\}$ NMR (151 MHz) spectrum of **1b** in C_6D_6.

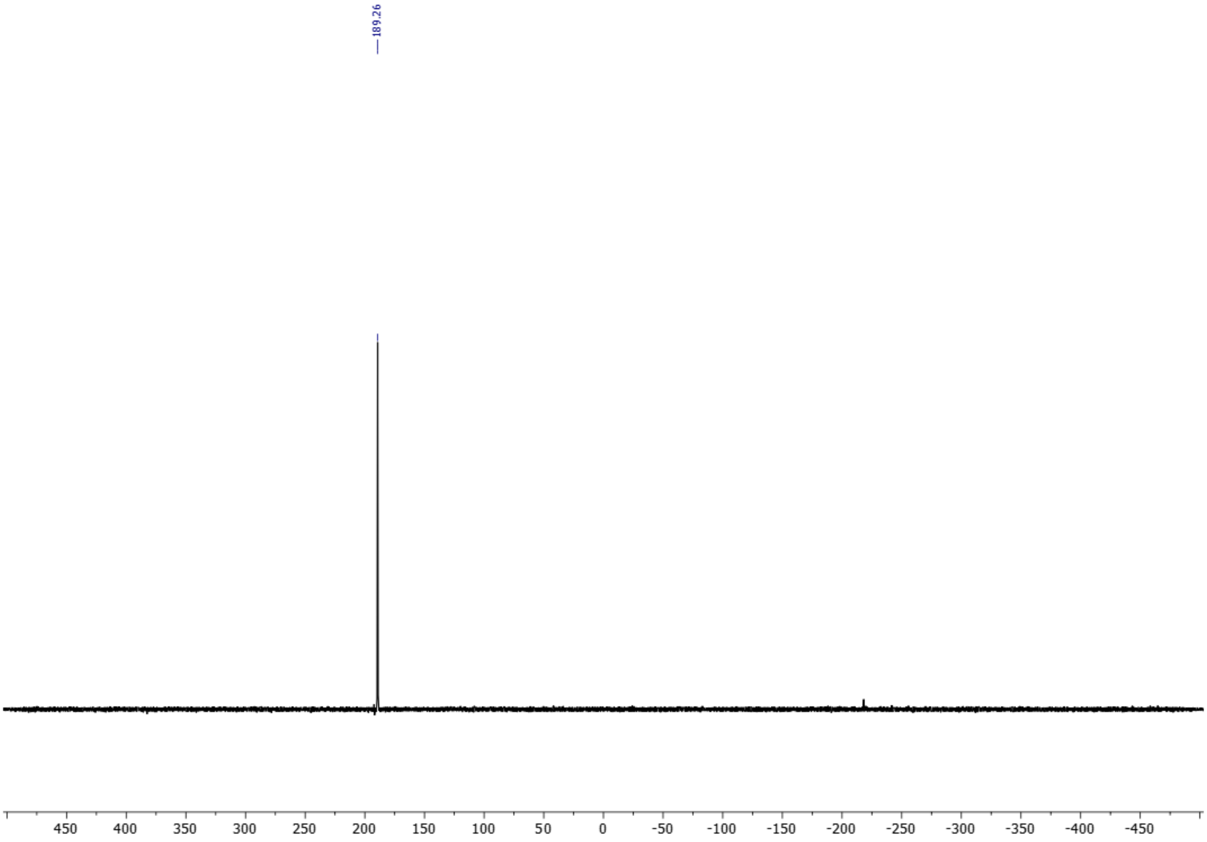

Figure S6. $^{31}P\{^{1}H\}$ NMR (162 MHz) spectrum of **1b** in C_6D_6.

1.2.3 Synthesis of Au(IDipp)(CPN$_3$Dipp) (1c)

Neat 2,6-diisopropylphenyl azide (17 mg, 0.08 mmol) was added to a solution of Au(IDipp)(CP) (50 mg, 0.08 mmol) in toluene (1 mL) and stirred at room temperature overnight. Crystals suitable for X-ray crystallography were obtained by concentrating the toluene solution followed by layering with hexane (5 mL). The supernatant solution was removed, and the crystals washed with pentane (2 × 2 mL), then dried under vacuum. Yield: 54 mg, 0.06 mmol, 81%. Anal. Calcd. (%) for $C_{40}H_{54}N_5AuP{\cdot}0.5(C_7H_8)$: C, 61.03; H, 6.76; N, 7.57. Found: C, 60.90; H, 6.49; N, 7.24.

^{1}H NMR (600 MHz, C_6D_6): δ(ppm) 7.12–7.09 (m, 2H, Dipp Ar**H**), 7.02–7.00 (m, 7H, Dipp Ar**H**), 6.32 (s, 2H, IDipp C**H**), 2.68 (sept, $^3J_{H\text{–}H}$ = 6.9 Hz, 4H, Dipp C**H**(CH$_3$)$_2$, 2.15 (sept, $^3J_{H\text{–}H}$ = 6.8 Hz, 2H, Dipp C**H**(CH$_3$)$_2$, 1.54 (d, $^3J_{H\text{–}H}$ = 6.9 Hz, 12H, Dipp CH(C**H**$_3$)$_2$, 1.08 (d, $^3J_{H\text{–}H}$ = 6.9 Hz, 12H, Dipp CH(C**H**$_3$)$_2$, 0.83 (d, $^3J_{H\text{–}H}$ = 6.8 Hz, 6H, Dipp CH(C**H**$_3$)$_2$,* 0.82 (d, $^3J_{H\text{–}H}$ = 6.8 Hz, 6H, Dipp CH(C**H**$_3$)$_2$.* *overlapping doublets.

^{13}C{^{1}H} NMR (151 MHz, C_6D_6): δ(ppm) 208.03 (d, $^1J_{C\text{–}P}$ = 83.3 Hz, **C**PN$_3$Ad), 195.75 (d, $^3J_{C\text{–}P}$ = 10.9 Hz, (IDipp {HCN(Dipp)}$_2$**C**Au), 146.56 (Dipp Ar-**C**), 145.73 (Dipp Ar-**C**), 138.13 (d, $^2J_{C\text{–}P}$ = 6.3 Hz, Dipp Ar**C**N), 134.66 (Dipp Ar-**C**), 130.77 (Dipp Ar-**C**), 128.95 (Dipp Ar-**C**), 124.30 (Dipp Ar-**C**), 123.45 (Dipp Ar-**C**), 122.80 (IDipp **C**H) , 29.14 (Dipp **C**H(CH$_3$)$_2$), 28.27 (Dipp **C**H(CH$_3$)$_2$), 24.99 (Dipp CH(**C**H$_3$)$_2$), 24.39 (Dipp CH(**C**H$_3$)$_2$), 24.18 (Dipp CH(**C**H$_3$)$_2$), 23.96 (Dipp CH(**C**H$_3$)$_2$).

^{31}P{^{1}H} NMR (162 MHz, C_6D_6): δ(ppm) 209.3 (s, C**P**N$_3$Dipp).

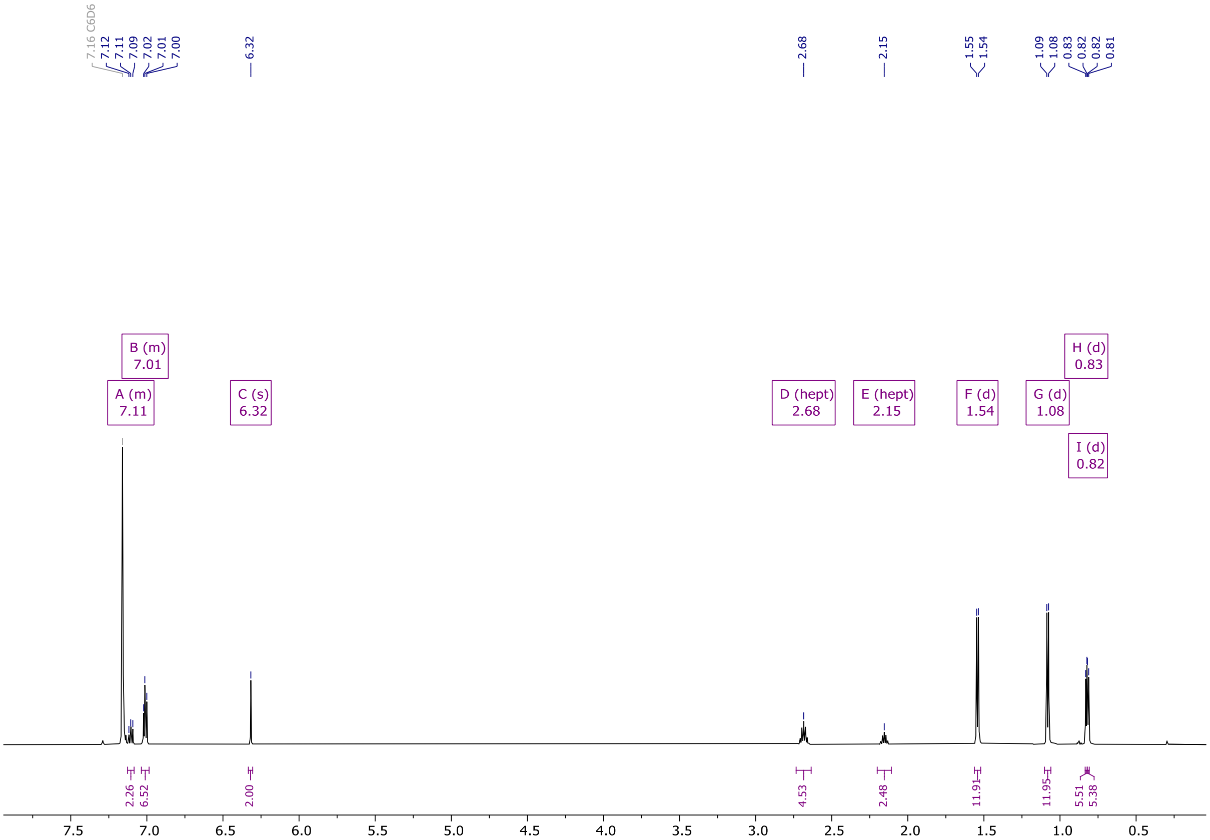

Figure S7. ^{1}H NMR (600 MHz) spectrum of **1c** in C_6D_6.

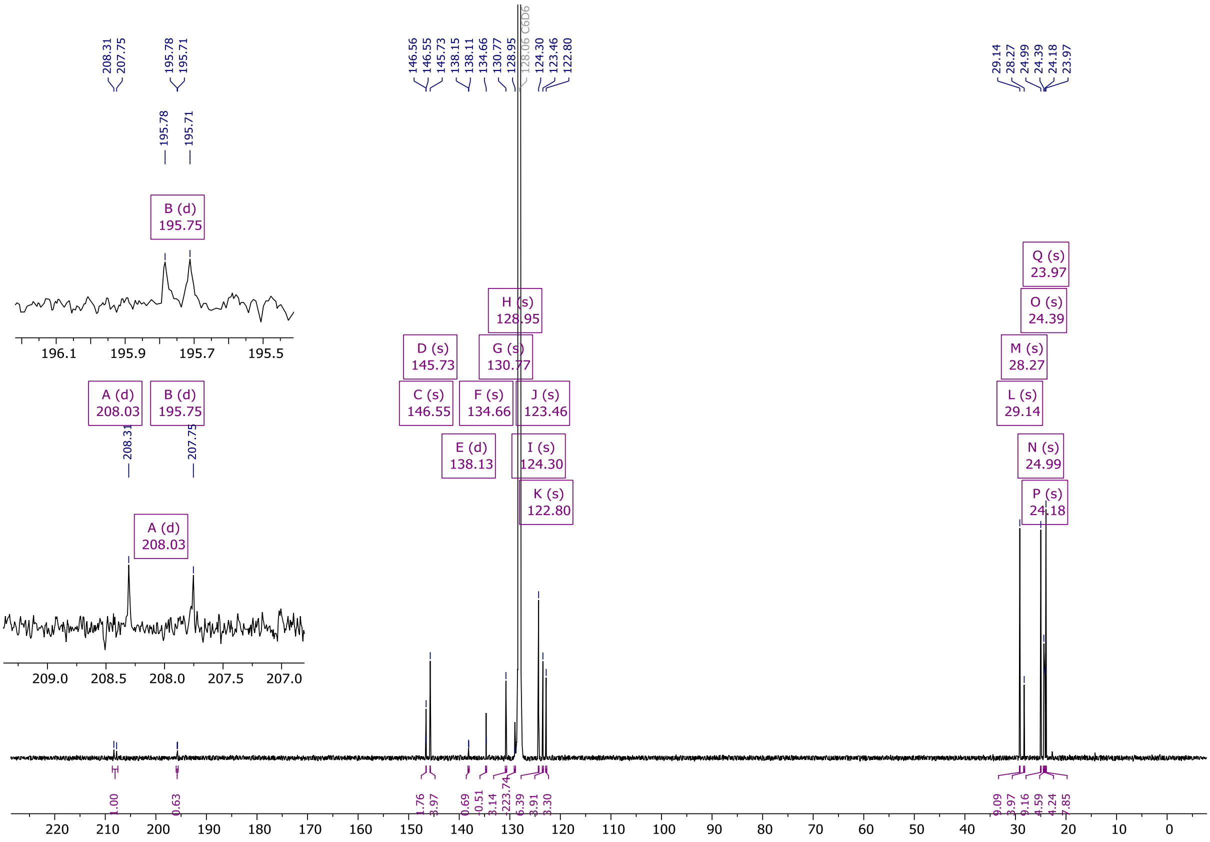

Figure S8. ^{13}C{^{1}H} NMR (151 MHz) spectrum of **1c** in C_6D_6.

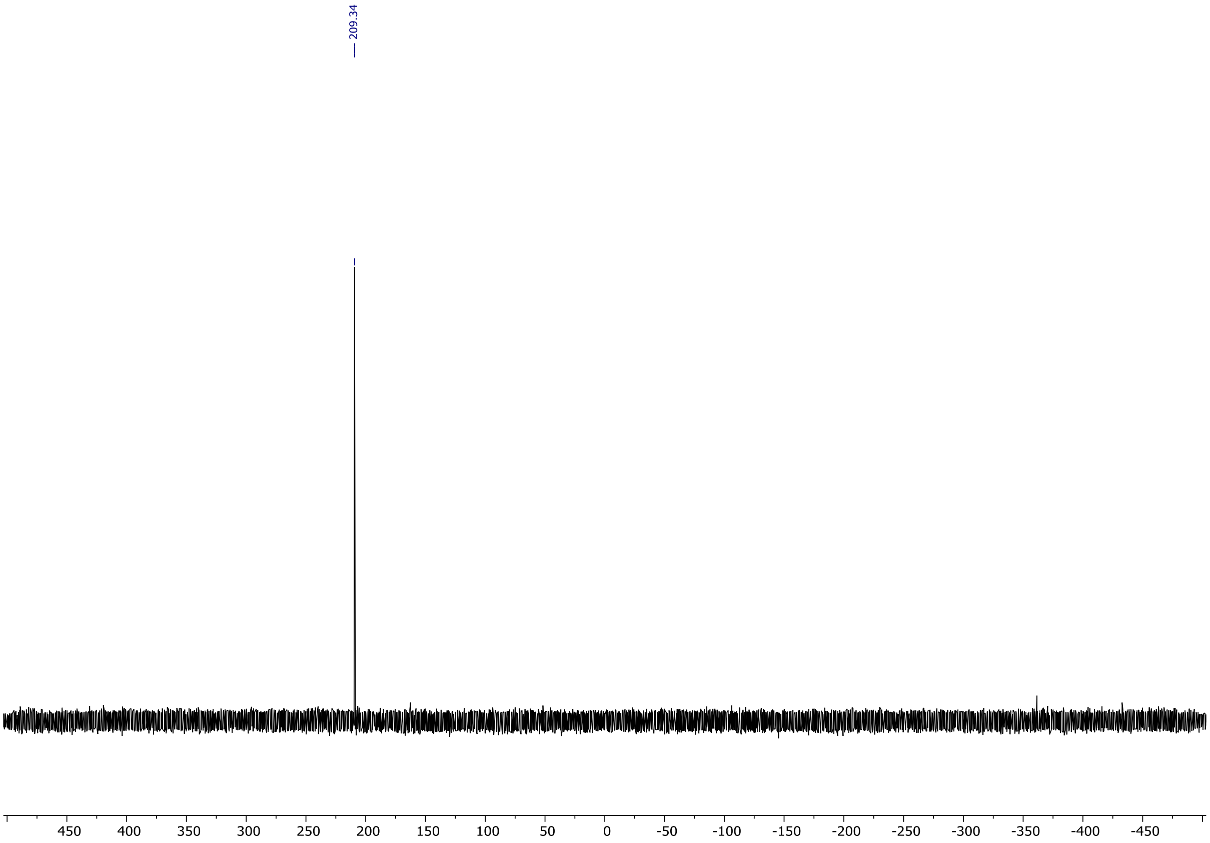

Figure S9. $^{31}P\{^1H\}$ NMR (162 MHz) spectrum of **1c** in C_6D_6.

1.2.4 Synthesis of $\{Au(IDipp)\}_2\{\mu\text{-}C_6H_4(CPN_3)_2\}$ (1d)

1,3-diazidobenzene (3.82 mg, 0.024 mmol) was added to a solution of Au(IDipp)(CP) (30 mg, 0.048 mmol) in toluene (1 mL) and stirred at room temperature overnight. The resulting precipitate was isolated by filtration. The filtrate was concentrated under vacuum, then stored at −35 ºC for 3 days to yield orange crystals which were isolated by filtration. Yield: 22 mg, 0.016 mmol, 63%. Anal. Calcd. (%) for $C_{62}H_{78}Au_2N_{10}P_2$: C, 52.47; H, 5.54; N, 9.87. Found: 52.74; H, 5.40; N, 9.16.

^{1}H NMR (600 MHz, CD_2Cl_2): δ(ppm) = 8.03 (s, 1H; Ar**H**), 7.64 (dd, $^3J_{H–H}$ = 8.1 Hz, $^4J_{H–H}$ = 2.1 Hz, 2H; Ar**H**), 7.55 (t, $^3J_{H–H}$ = 7.8 Hz, 4H; Dipp *para* C**H**), 7.39 (t, $^3J_{H–H}$ = 8.1 Hz, 1H; Ar**H**), 7.36 (d, $^3J_{H–H}$ = 7.8 Hz 8H; Dipp *meta* C**H**), 7.25 (s, 4H; IDipp C**H**), 2.69 (sept, $^3J_{H–H}$ = 6.9 Hz, 8H; Dipp C**H**(CH_3)$_2$), 1.41 (d, $^3J_{H–H}$ = 6.9 Hz, 24H; Dipp CH(C**H**$_3$)$_2$), 1.26 (d, $^3J_{H–H}$ = 6.9 Hz, 24H; Dipp CH(C**H**$_3$)$_2$).

$^{13}C\{^1H\}$ NMR (151 MHz, CD_2Cl_2): δ(ppm) = 208.21 (d, $^1J_{C–P}$ = 84.1 Hz; **C**P), 194.23 (d, $^3J_{C–P}$ = 10.0 Hz; {HCN(Dipp)}$_2$**C**Au), 146.32 (Dipp *ortho* **C**), 143.16 (d, $J_{C–P}$ = 7.6 Hz; Ar**C**) 134.77 (Dipp *ipso* **C**), 130.85 (Dipp *para* **C**), 130.36 (Ar**C**), 124.52 (Dipp *meta* **C**), 123.80 (IDipp **C**H),

120.73 (d, $^4J_{C\text{–}P}$ = 5.4 Hz; Ar**C**), 115.84 (t, $^4J_{C\text{–}P}$ = 6.5 Hz; Ar**C**), 29.28 (Dipp **C**H(CH$_3$)$_2$), 24.73 (Dipp CH(**C**H$_3$)$_2$), 24.16 (Dipp CH(**C**H$_3$)$_2$).

^{31}P{^{1}H} NMR (162 MHz, CD$_2$Cl$_2$): δ(ppm) 200.8 (C**P**N$_3$).

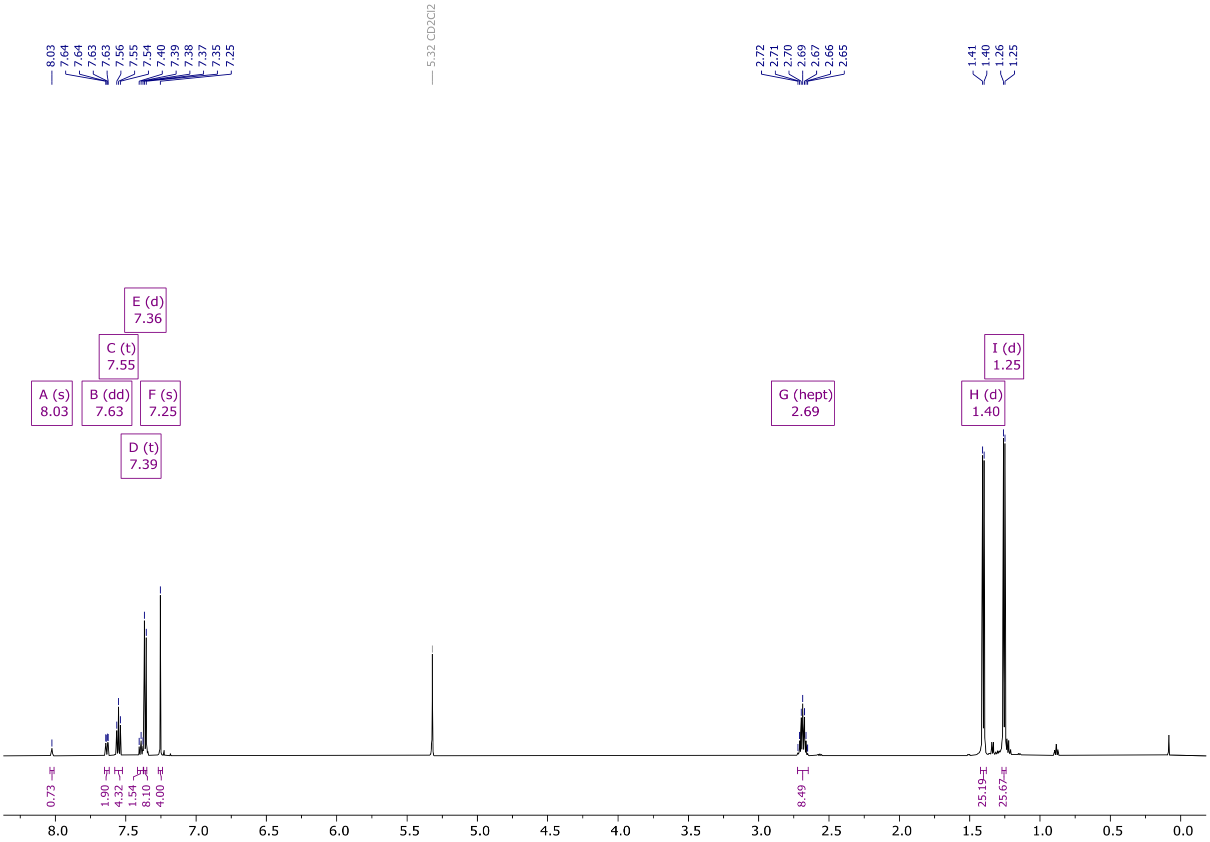

Figure S10. ^{1}H NMR (400 MHz) spectrum of **1d** in C_6D_6.

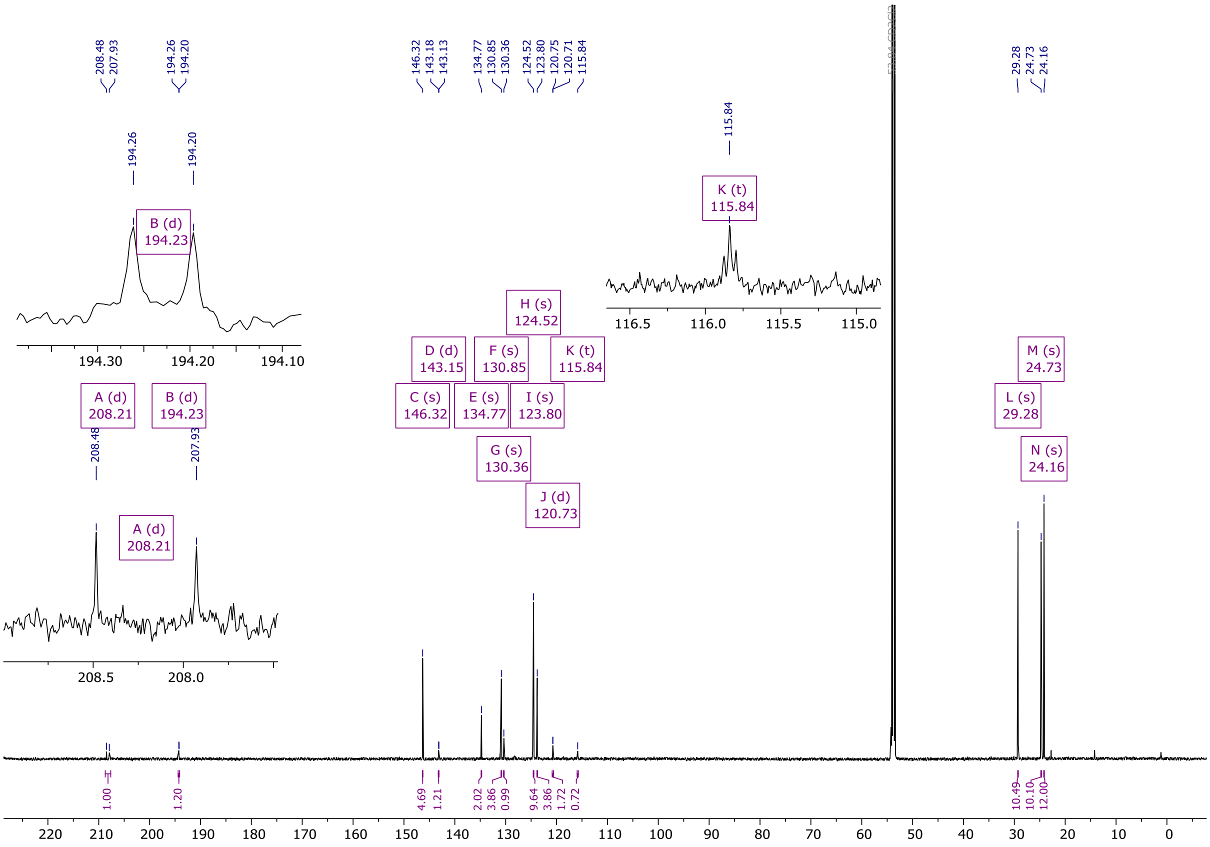

Figure S11. $^{13}C\{^{1}H\}$ NMR (151 MHz) spectrum of **1d** in C_6D_6.

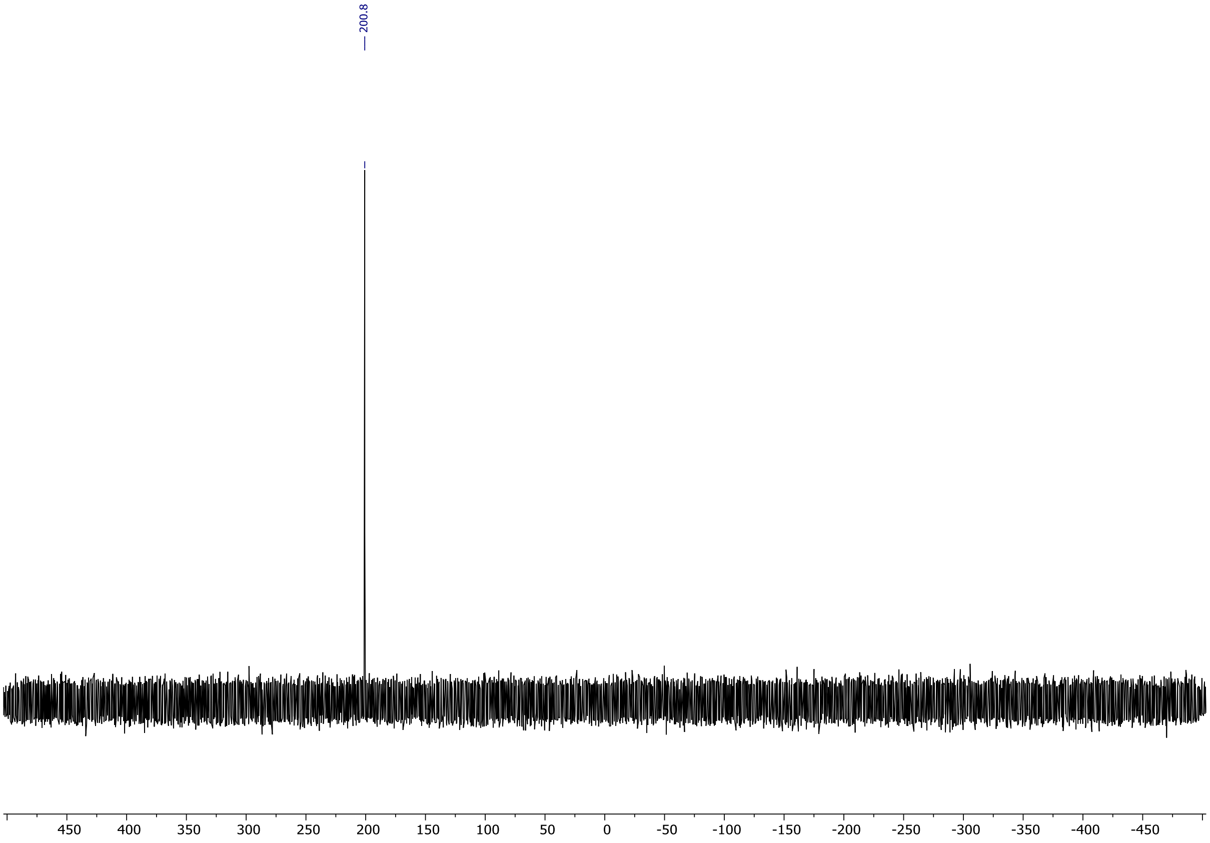

Figure S12. $^{31}P\{^{1}H\}$ NMR (162 MHz) spectrum of **1d** in CD_2Cl_2.

1.2.5 Synthesis of {Mg(^{Dipp}NacNac)(CPN$_3$^{t}Bu)}$_2$ (2a)

Mg(^{Dipp}NacNac)(dioxane)(CP) (approx. 130 mg, 0.23 mmol) was generated *in situ* in toluene (1 mL). Neat *tert*-butyl azide (23 mg, 0.23 mmol) was added, and the solution swirled to ensure complete mixing. The reaction solution was allowed to stand overnight, resulting in the formation of colorless crystals suitable for single crystal X-ray diffraction. The supernatant was decanted, and the crystals were washed with hexane (3 × 1 mL), then dried under vacuum. Yield: 120 mg, 0.10 mmol, 91%. Anal. Calcd. (%) for $C_{68}H_{100}Mg_2N_{10}P_2 \cdot C_6H_{14}$: C, 70.86; H, 9.16; N, 11.17. Found: C, 70.68; H, 8.56; N, 10.84.

^{1}H NMR (400 MHz, C$_6$D$_6$): δ(ppm) 7.12–6.95 (m, 8H, Dipp *meta* C**H**), 6.94–6.87 (m, 4H, Dipp *para* C**H**), 4.97 (s, 2H, NacNac C**H**{C(CH$_3$)(NDipp)}$_2$), 3.88 (sept, $^3J_{H-H}$ = 6.8 Hz, 4H, Dipp C**H**(CH$_3$)$_2$), 2.57 (sept, $^3J_{H-H}$ = 6.8 Hz, 4H, Dipp C**H**(CH$_3$)$_2$), 1.67 (d, $^3J_{H-H}$ = 6.8 Hz, 12H, Dipp CH(C**H**$_3$)$_2$), 1.66 (s, 12H), 1.54 (s, 18H), 1.30 (d, $^3J_{H-H}$ = 6.8 Hz, 12H, Dipp CH(C**H**$_3$)$_2$), 0.99 (d, $^3J_{H-H}$ = 6.8 Hz, 12H, Dipp CH(C**H**$_3$)$_2$), −0.64 (d, $^3J_{H-H}$ = 6.8 Hz, 12H, Dipp CH(C**H**$_3$)$_2$).

^{13}C{^{1}H} NMR (126 MHz, C$_6$D$_6$): δ(ppm) 207.15 (d, $^1J_{C-P}$ = 93.3 Hz, **C**PN$_3$^{t}Bu), 168.23 (NacNac CH{**C**(CH$_3$)(NDipp)}$_2$), 146.62 (Dipp *ipso* **C**), 143.17 (Dipp *ortho* **C**), 141.73 (Dipp *ortho* **C**), 124.66 (Dipp *meta* **C**), 124.21 (Dipp *para* **C**), 122.92 (Dipp *meta* **C**), 95.03 (NacNac **C**H{C(CH$_3$)(NDipp)}$_2$), 61.08 (d, $^2J_{C-P}$ = 2.9 Hz, ^{t}Bu **C**(CH$_3$)$_3$), 31.77 (d, $^3J_{C-P}$ = 4.6 Hz, ^{t}Bu C(**C**H$_3$)$_3$), 30.04 (Dipp **C**H(CH$_3$)$_2$), 27.60 (Dipp **C**H(CH$_3$)$_2$), 25.09 (Dipp CH(**C**H$_3$)$_2$), 24.66 (Dipp CH(**C**H$_3$)$_2$), 24.44 (Dipp CH(**C**H$_3$)$_2$), 24.23 (NacNac CH{C(**C**H$_3$)(NDipp)}$_2$), 24.05 (Dipp CH(**C**H$_3$)$_2$).

^{31}P{^{1}H} NMR (162 MHz, C$_6$D$_6$): δ(ppm) 224.3 (s, C**P**N$_3$^{t}Bu).

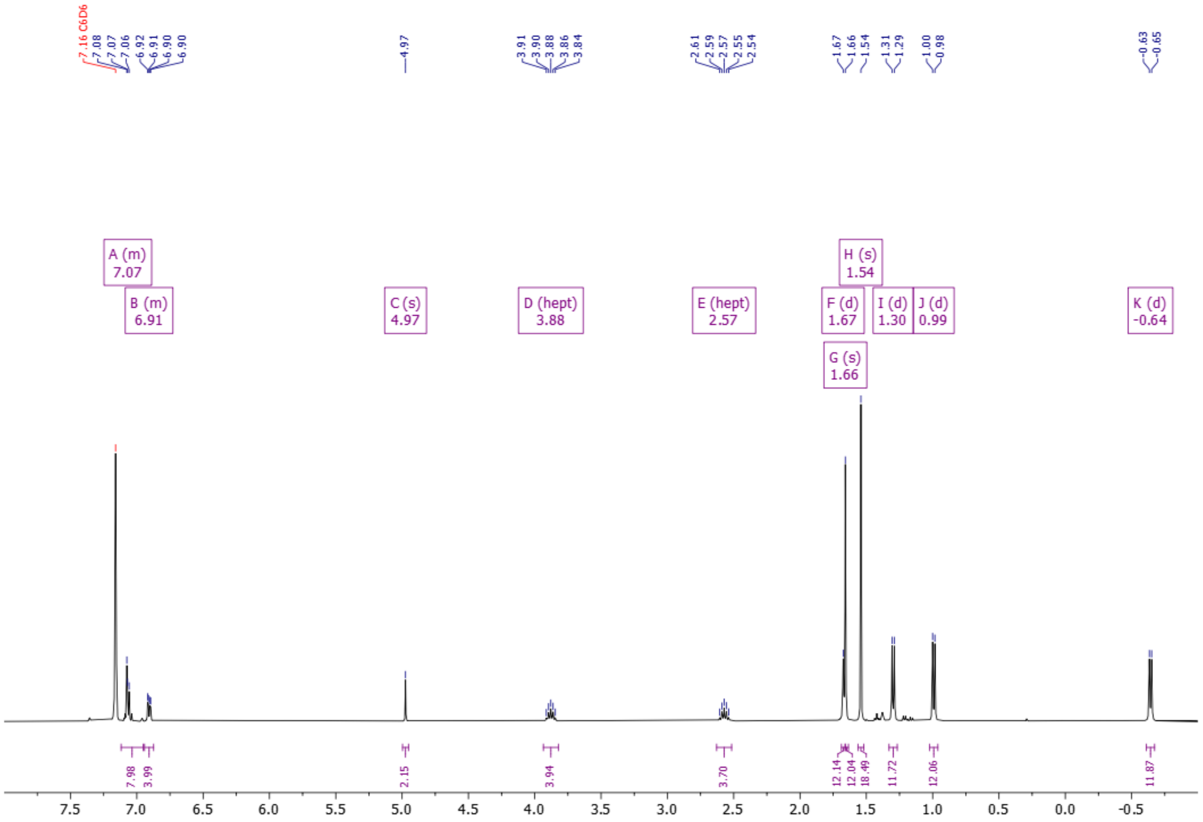

Figure S13. ^{1}H NMR (400 MHz) spectrum of **2a** in C_6D_6.

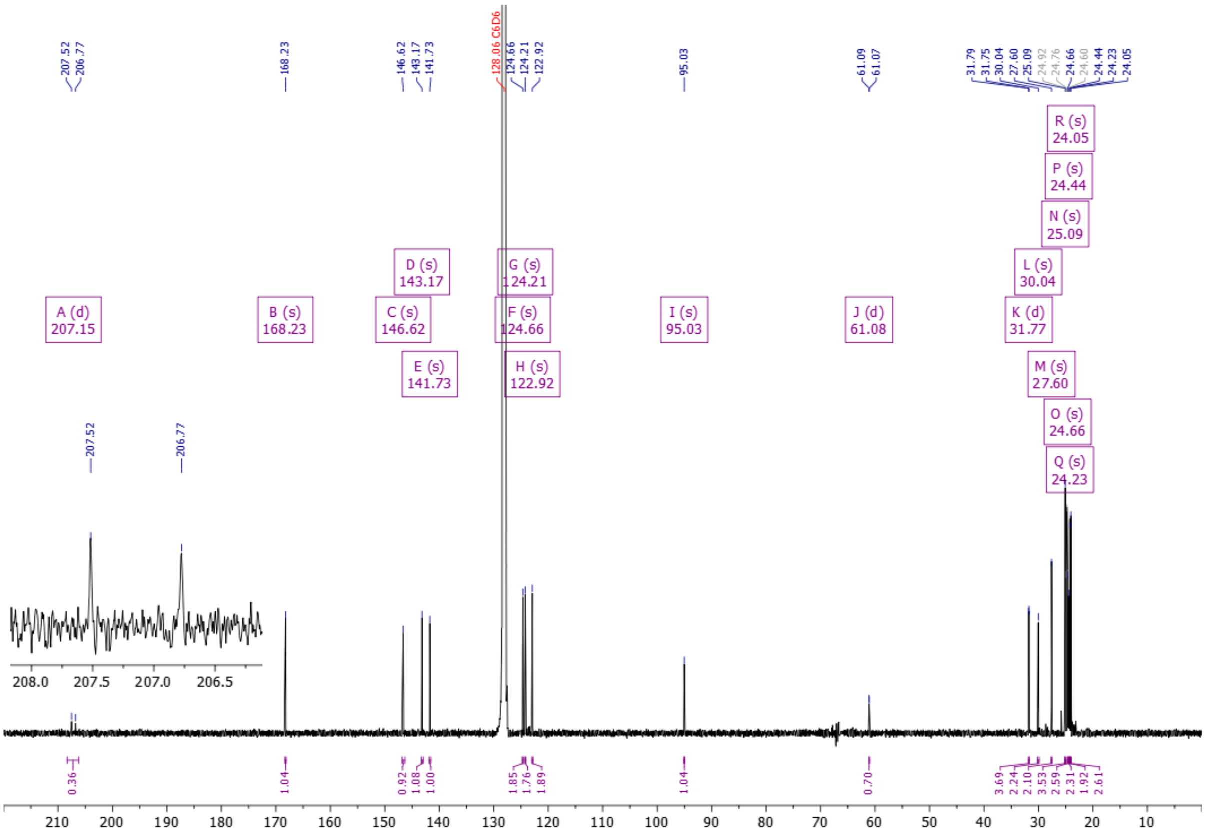

Figure S14. $^{13}C\{^1H\}$ NMR (126 MHz) spectrum of **2a** in C_6D_6.

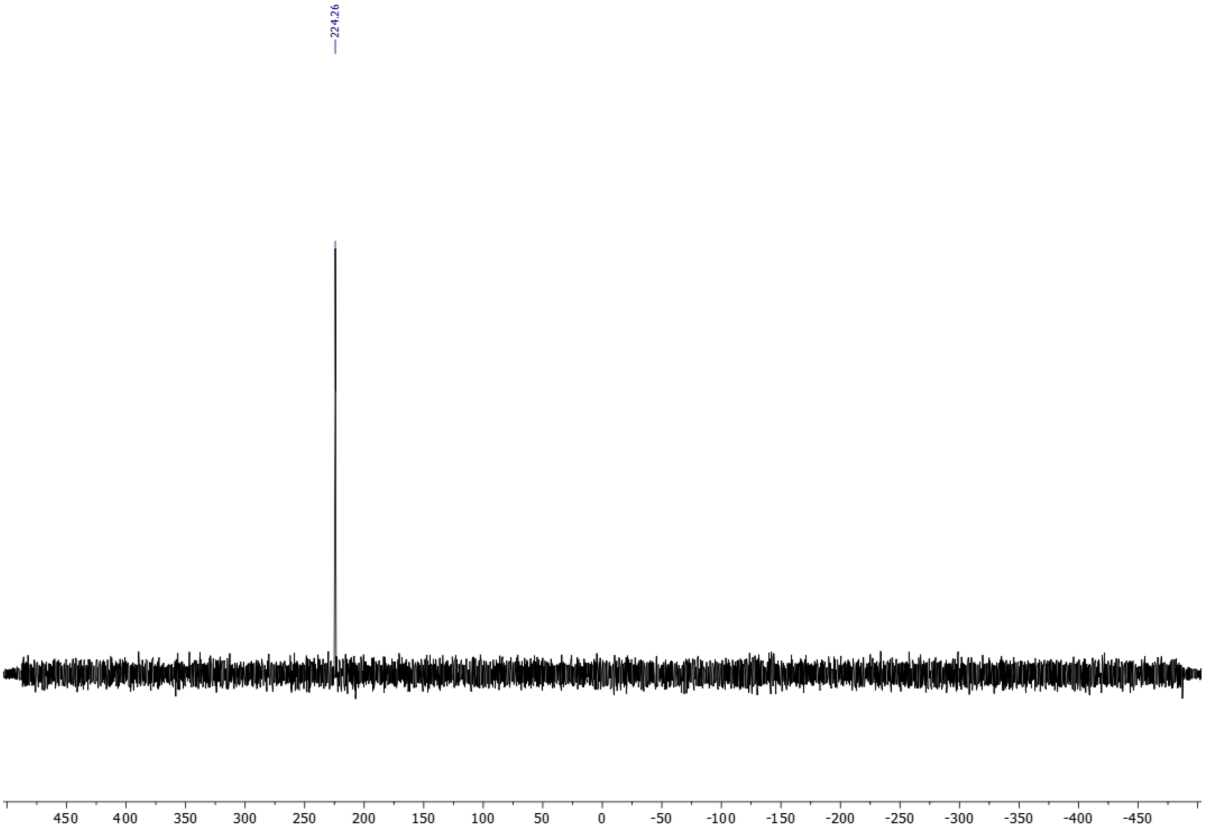

Figure S15. $^{31}P\{^{1}H\}$ NMR (162 MHz) spectrum of **2a** in C_6D_6.

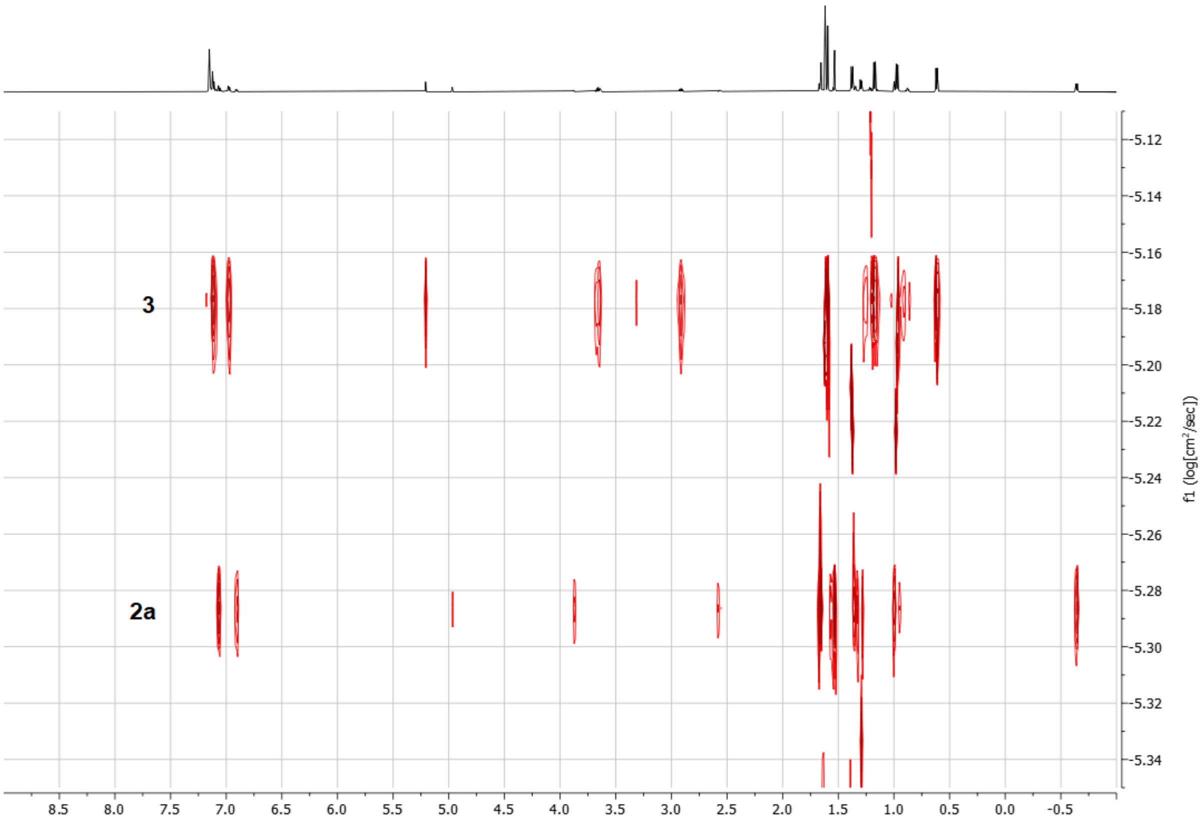

Figure S16. ^{1}H DOSY NMR (500 MHz) spectrum of a mixture of **2a** and **3** in C_6D_6.

Table S1. Diffusion coefficients and calculated hydrodynamic radii of **2a** and **3**.

	**2a**		**3**
D / m^2 sec^{-1}	5.2×10^{-10}		6.7×10^{-10}
R_h (calc) / Å	7.4		6.1
D_3/D_{2a}		**1.3**	
R_{h2a}/R_{h3}		**1.2**	
Estimated M_w[37] / g mol^{-1}	1073		612
Actual M_w / g mol^{-1}	1168		632

1.2.6 Synthesis of {Mg(^{Dipp}NacNac)(CPN$_3$Bn)}$_2$ (2b)

Mg(^{Dipp}NacNac)(dioxane)(CP) (approx. 65 mg, 0.11 mmol) was generated *in situ* in toluene (1 mL). Neat benzyl azide (14 mg, 0.11 mmol) was added, and the solution swirled to ensure complete mixing. The reaction solution was allowed to stand for 3 h, resulting in the formation of colorless crystals. The supernatant was decanted, and the crystals were washed with hexane (3 × 1 mL), then dried under vacuum. Yield: 30 mg, 0.05 mmol, 43%. Anal. Calcd. (%) for $C_{80}H_{112}Mg_2N_{10}P_2$: C, 72.55; H, 8.52; N, 10.58. Found: C, 72.55; H, 8.52; N, 10.58. The low solubility of {Mg(^{Dipp}NacNac)(CPN$_3$Bn)}$_2$ in polar organic solvents e.g. THF, DMSO, and DCM, precluded characterization by NMR spectroscopy.

1.2.7 Synthesis of Ge(^{Dipp}NacNac)(CPN$_3$tBu) (3)

Mg(^{Dipp}NacNac)(dioxane)(CP) (approx. 42 mg, 0.07 mmol) was generated *in situ* in toluene (1 mL). Ge(^{Dipp}NacNac)Cl (38 mg, 0.07 mmol) was added, and the reaction mixture stirred for 3 h. The reaction mixture was filtered, then *tert*-butyl azide (7 mg, 0.07 mmol) was added to the filtrate. The reaction solution was stirred overnight, then the solvent removed under vacuum. The residue was washed with hexane (3 × 1 mL), then recrystallized from toluene at −35 °C over 3 days. Yield: 32 mg, 0.06 mmol, 70%. Anal. Calcd. (%) for $C_{30}H_{41}GeN_2P$: C, 64.57; H, 7.97; N, 11.07. Found: C, 65.11; H, 8.28; N, 10.68.

^{1}H NMR (500 MHz, C_6D_6): δ(ppm) 7.16–7.09 (m, 4H, Dipp *meta* C**H**), 6.98 (dd, $^3J_{H\text{-}H}$ = 6.3, 2.9 Hz, 2H, Dipp *para* C**H**), 5.22 (s, 1H, NacNac C**H**{C(CH$_3$)(NDipp)}$_2$), 3.66 (sept, $^3J_{H\text{-}H}$ = 6.8 Hz, 2H, Dipp C**H**(CH$_3$)$_2$), 2.92 (sept, $^3J_{H\text{-}H}$ = 6.8 Hz, 2H, Dipp C**H**(CH$_3$)$_2$), 1.63 (s, 9H, ^{t}Bu C(C**H**$_3$)$_3$), 1.60 (s, 6H, NacNac CH{C(C**H**$_3$)(NDipp)}$_2$), 1.38 (d, $^3J_{H\text{-}H}$ = 6.8 Hz, 6H, Dipp CH(C**H**$_3$)$_2$), 1.18 (d, $^3J_{H\text{-}H}$ = 6.8 Hz, 6H, Dipp CH(C**H**$_3$)$_2$), 0.98 (d, $^3J_{H\text{-}H}$ = 6.8 Hz, 6H, Dipp CH(C**H**$_3$)$_2$), 0.62 (d, $^3J_{H\text{-}H}$ = 6.8 Hz, 6H, Dipp CH(C**H**$_3$)$_2$).

^{13}C{^{1}H} NMR (126 MHz, C_6D_6): δ(ppm) 199.39 (d, $^1J_{C\text{-}P}$ = 105.1 Hz, **C**PN$_3$^{t}Bu), 166.67 (NacNac CH{**C**(CH$_3$)(NDipp)}$_2$), 146.34 (Dipp *ipso* **C**), 143.64 (Dipp *ortho* **C**), 141.58 (Dipp

ortho **C**), 127.47 (Dipp *meta* **C**), 124.90 (Dipp *para* **C**), 124.41 (Dipp *meta* **C**), 100.13 (d, $^{5}J_{C-P}$ = 6.7 Hz, NacNac **C**H{C(CH$_3$)(NDipp)}$_2$), 60.54 (d, $^{2}J_{C-P}$ = 4.5 Hz, ^{t}Bu **C**(CH$_3$)$_3$), 32.39 (d, $^{2}J_{C-P}$ = 5.7 Hz, ^{t}Bu C(**C**H$_3$)$_3$), 29.41 (Dipp **C**H(CH$_3$)$_2$), 28.70 (d, $^{6}J_{C-P}$ = 3.9 Hz, Dipp **C**H(CH$_3$)$_2$), 26.01 (Dipp CH(**C**H$_3$)$_2$), 24.92 (Dipp CH(**C**H$_3$)$_2$), 24.73 (Dipp CH(**C**H$_3$)$_2$), 23.65 (NacNac CH{C(**C**H$_3$)(NDipp)}$_2$/Dipp CH(**C**H$_3$)$_2$), 23.64 (NacNac CH{C(**C**H$_3$)(NDipp)}$_2$/Dipp CH(**C**H$_3$)$_2$).

^{31}P{^{1}H} NMR (162 MHz, C_6D_6): δ(ppm) 196.9 (s, C**P**N$_3$^{t}Bu).

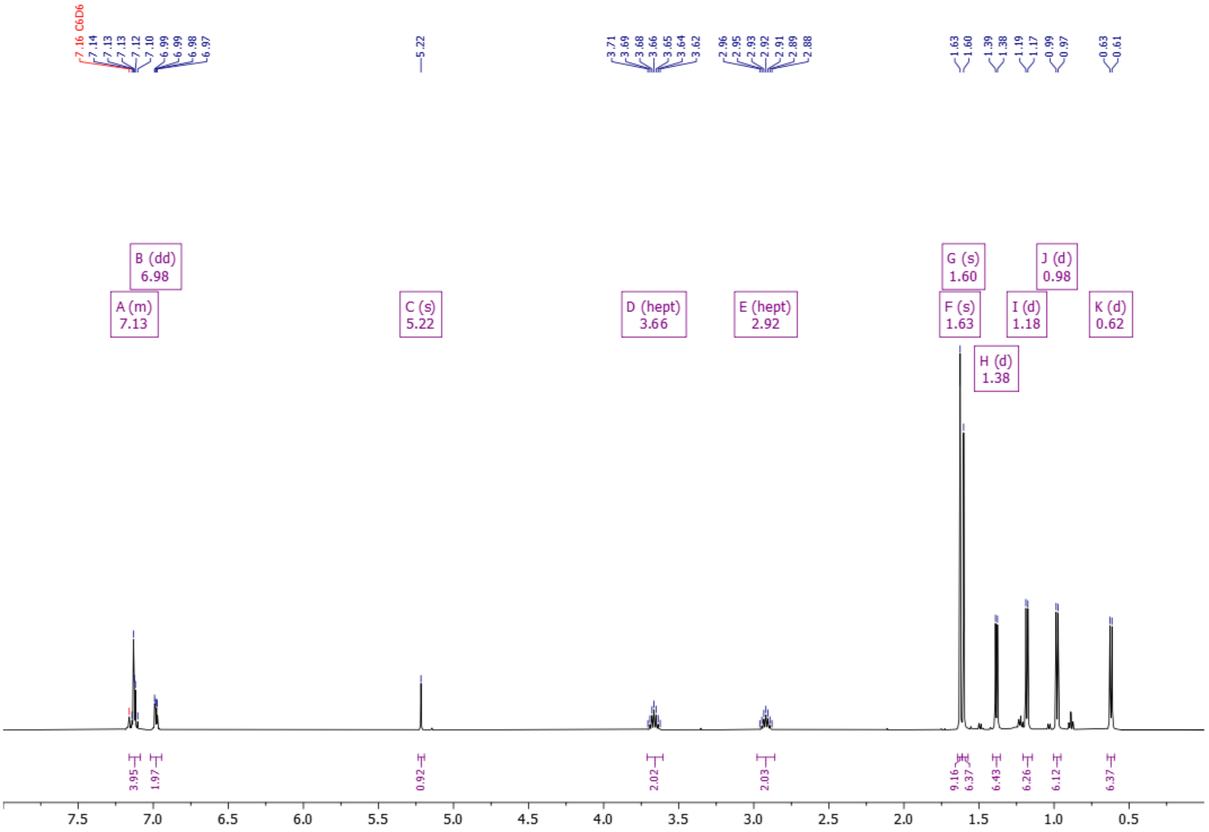

Figure S17. ^{1}H NMR (400 MHz) spectrum of **3** in C_6D_6.

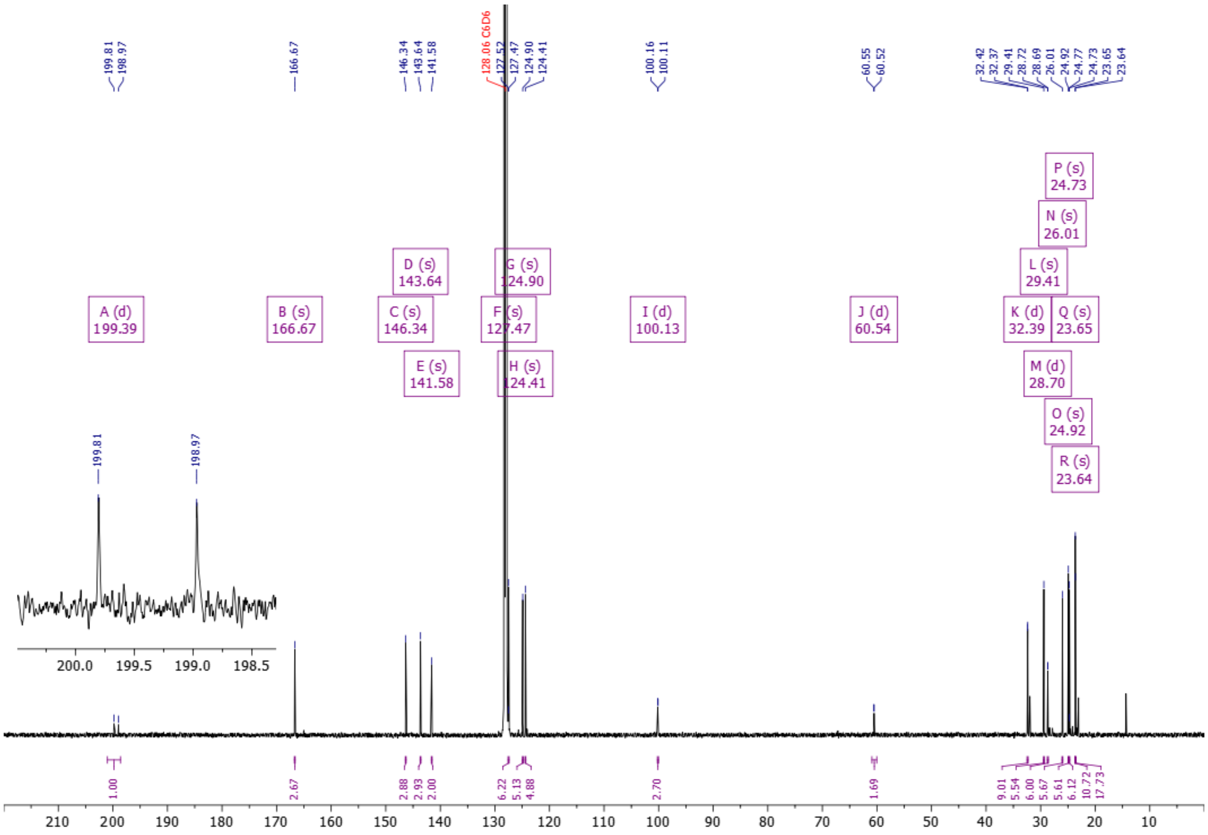

Figure S18. $^{13}C\{^1H\}$ NMR (126 MHz) spectrum of **3** in C_6D_6.

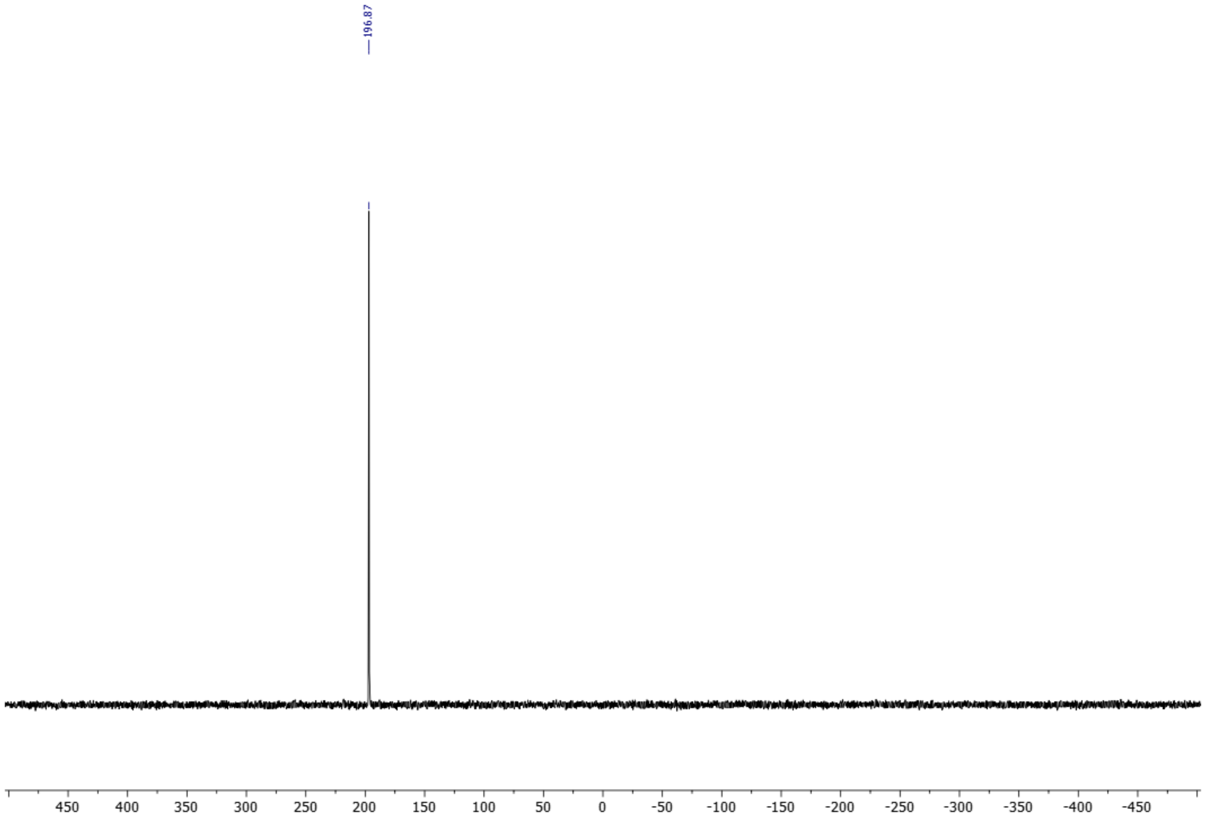

Figure S19. $^{31}P\{^1H\}$ NMR (162 MHz) spectrum of **3** in C_6D_6.

1.2.8 Synthesis of Au(IDipp)(CPN$_3$^{t}Bu)(B(C$_6$F$_6$)$_3$) (4a)

Au(IDipp)(CPN$_3$^{t}Bu) (20 mg, 0.03 mmol) and B(C$_6$F$_5$)$_3$ (14 mg, 0.03 mmol) were dissolved in toluene (0.5 mL). The reaction mixture was stirred for 1 h, then concentrated by slow evaporation. Storage at −35 ºC over 7 days yielded colorless crystals which were isolated by filtration, washed with pentane (3 × 0.5 mL), then dried under vacuum. Yield: 19 mg, 0.02 mmol, 56%. Anal. Calcd. (%) for $C_{50}H_{45}AuBF_{15}N_5P\cdot2(C_7H_8)$: C, 54.02; H, 4.25; N, 4.92. Found: C, 53.95 H, 3.82; N, 5.25.

^{1}H NMR (600 MHz, C$_6$D$_6$): δ(ppm) 7.34 (t, $^3J_{H-H}$ = 7.8 Hz, 2H, Dipp *para* C**H**), 7.06 (d, $^3J_{H-H}$ = 7.8 Hz, 4H, Dipp *meta* C**H**), 6.28 (s, 2H, IDipp C**H**), 2.55 (b, 4H, Dipp C**H**(CH$_3$)$_2$), 1.35 (b, 12H, Dipp CH(C**H**$_3$)$_2$), 1.00 (s, 9H, ^{t}Bu C(C**H**$_3$)$_3$), 0.95 (d, $^3J_{H-H}$ = 6.9 Hz, 12H, Dipp CH(C**H**$_3$)$_2$).

^{13}C{^{1}H} NMR (151 MHz, C$_6$D$_6$): δ(ppm) 207.25 (d, $^1J_{C-P}$ = 77.0 Hz, **C**PN$_3$^{t}Bu), 190.92 (d, $^3J_{C-P}$ = 3.5 Hz, IDipp {HCN(Dipp)}$_2$**C**Au), 149.88 (b, B(**C**$_6$F$_5$)$_3$), 148.30 (b, B(**C**$_6$F$_5$)$_3$), 145.66 (Dipp *ortho* C**H**), 140.69 (b, B(**C**$_6$F$_5$)$_3$), 139.11 (b, B(**C**$_6$F$_5$)$_3$), 137.90 (b, B(**C**$_6$F$_5$)$_3$), 136.33 (b, B(**C**$_6$F$_5$)$_3$), 134.54 (Dipp *ipso* C**H**), 130.92 (Dipp *para* C**H**), 124.31 (Dipp *meta* C**H**), 123.92 (IDipp **C**H), 63.73 (^{t}Bu **C**(CH$_3$)$_3$), 30.33 (d, $^3J_{C-P}$ = 5.1 Hz, ^{t}Bu C(**C**H$_3$)$_3$), 29.01 (Dipp **C**H(CH$_3$)$_2$), 24.82 (Dipp CH(**C**H$_3$)$_2$), 23.68 (Dipp CH(**C**H$_3$)$_2$).

^{31}P{^{1}H} NMR (162 MHz, C$_6$D$_6$): δ(ppm) 203.5 ppm (s, C**P**N$_3$^{t}Bu)

^{19}F{^{1}H} NMR (377 MHz, C$_6$D$_6$): δ(ppm) −124.46 to −135.30 (m, B(C$_6$F$_5$)$_3$ *ortho* C**F**), −156.82– −161.17 (m, B(C$_6$F$_5$)$_3$ *para* C**F**), −161.18 to −168.19 (m, B(C$_6$F$_5$)$_3$ *meta* C**F**)

^{11}B{^{1}H} NMR (128 MHz, C$_6$D$_6$): δ(ppm) −5.52 ppm (b, **B**(C$_6$F$_5$)$_3$)

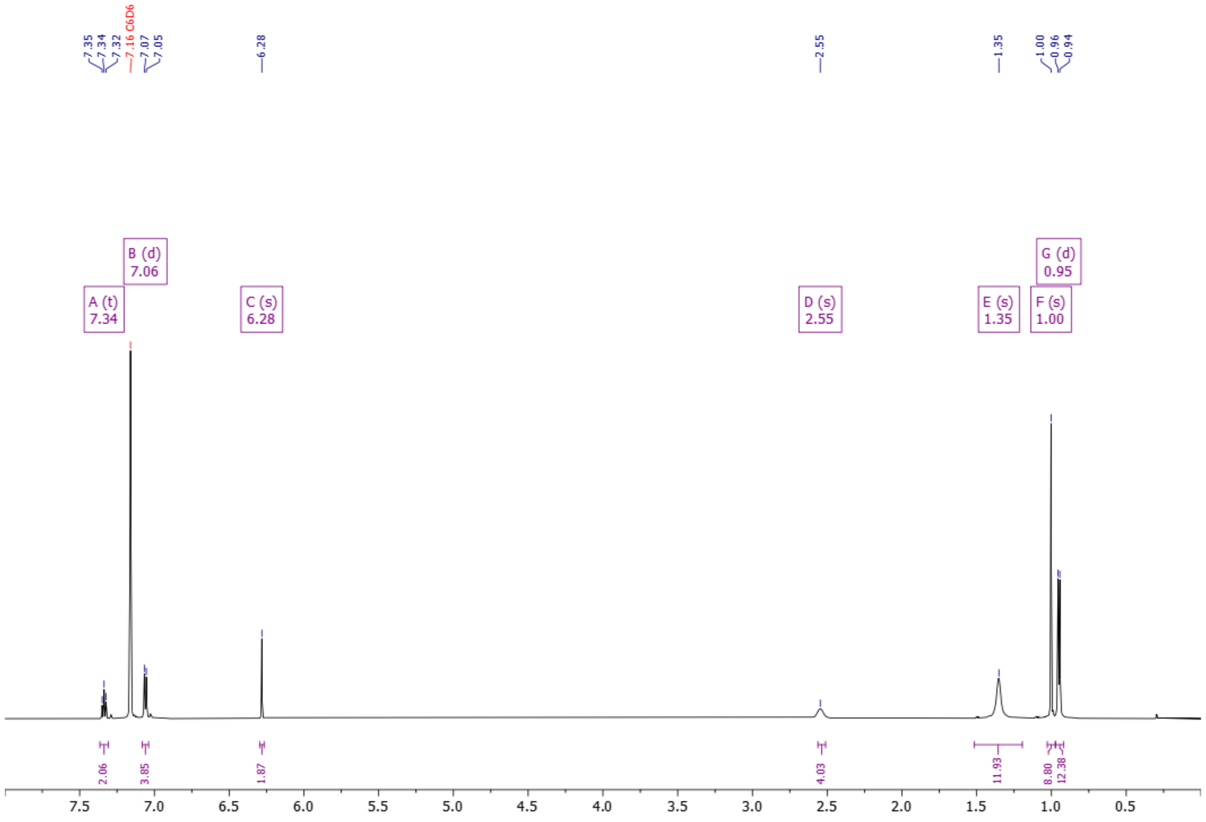

Figure S20. ^{1}H NMR (400 MHz) spectrum of **4a** in C_6D_6.

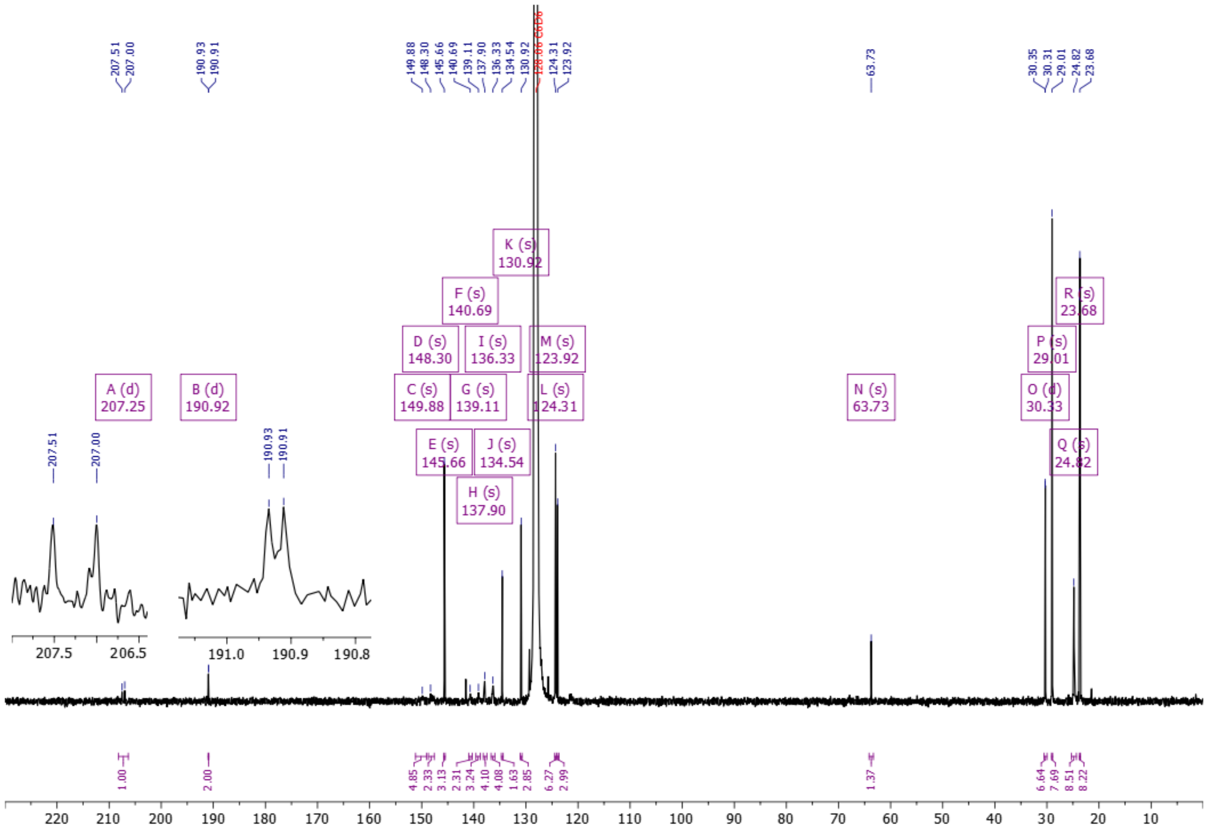

Figure S21. $^{13}C\{^1H\}$ NMR (151 MHz) spectrum of **4a** in C_6D_6.

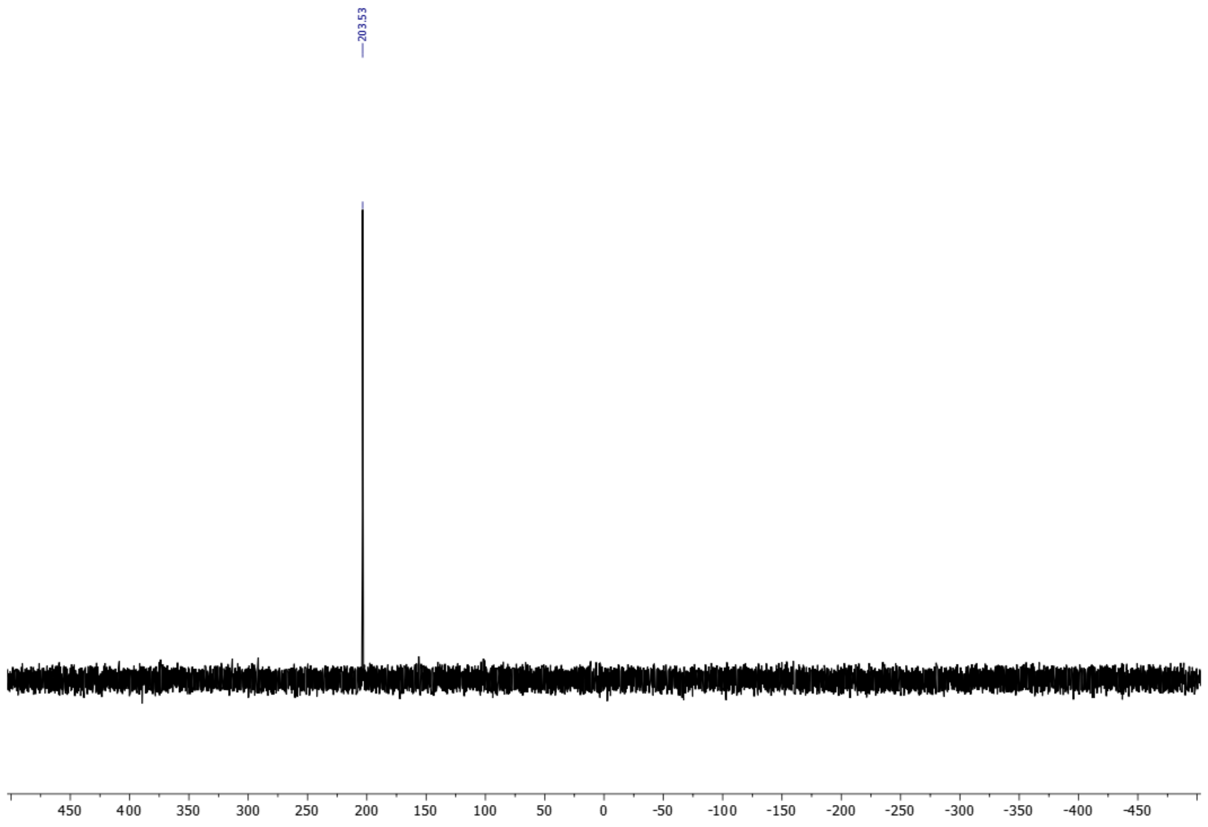

Figure S22. $^{31}P\{^{1}H\}$ NMR (162 MHz) spectrum of **4a** in C_6D_6.

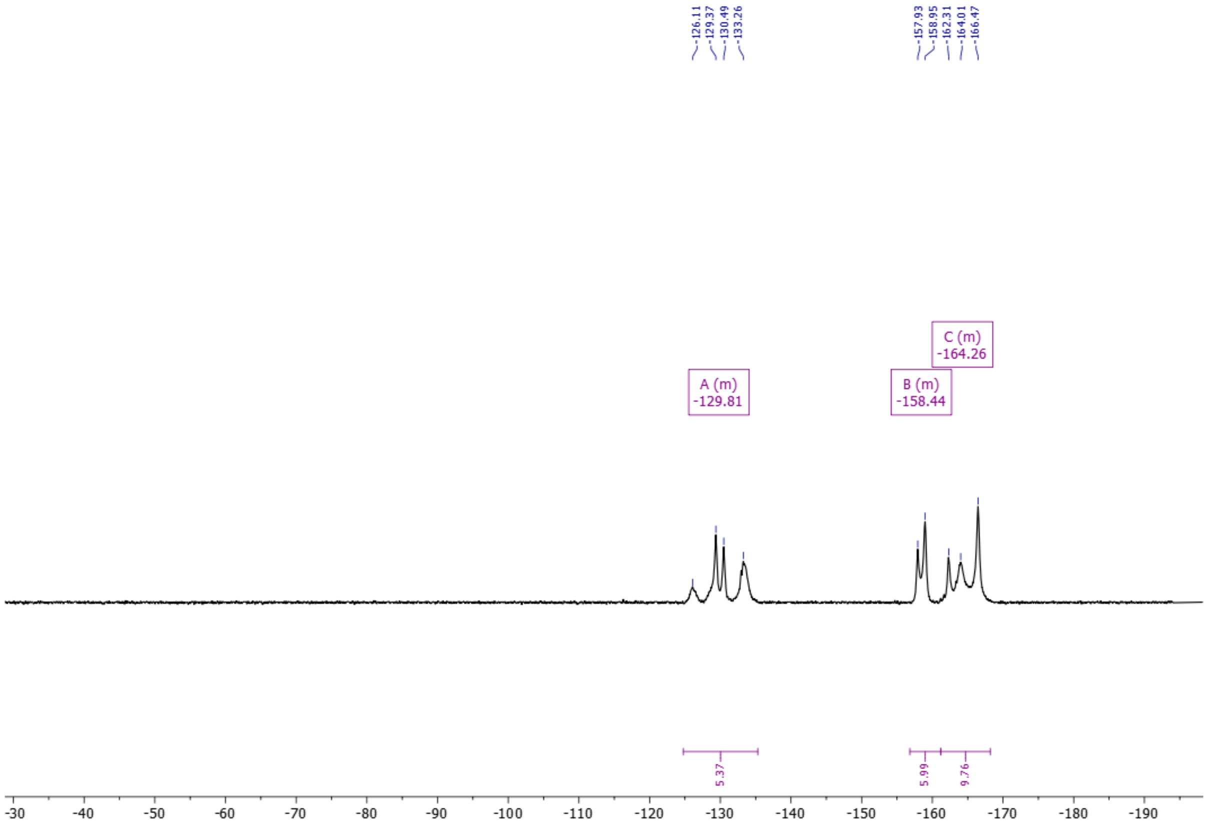

Figure S23. $^{19}F\{^{1}H\}$ NMR (377 MHz) spectrum of **4a** in C_6D_6.

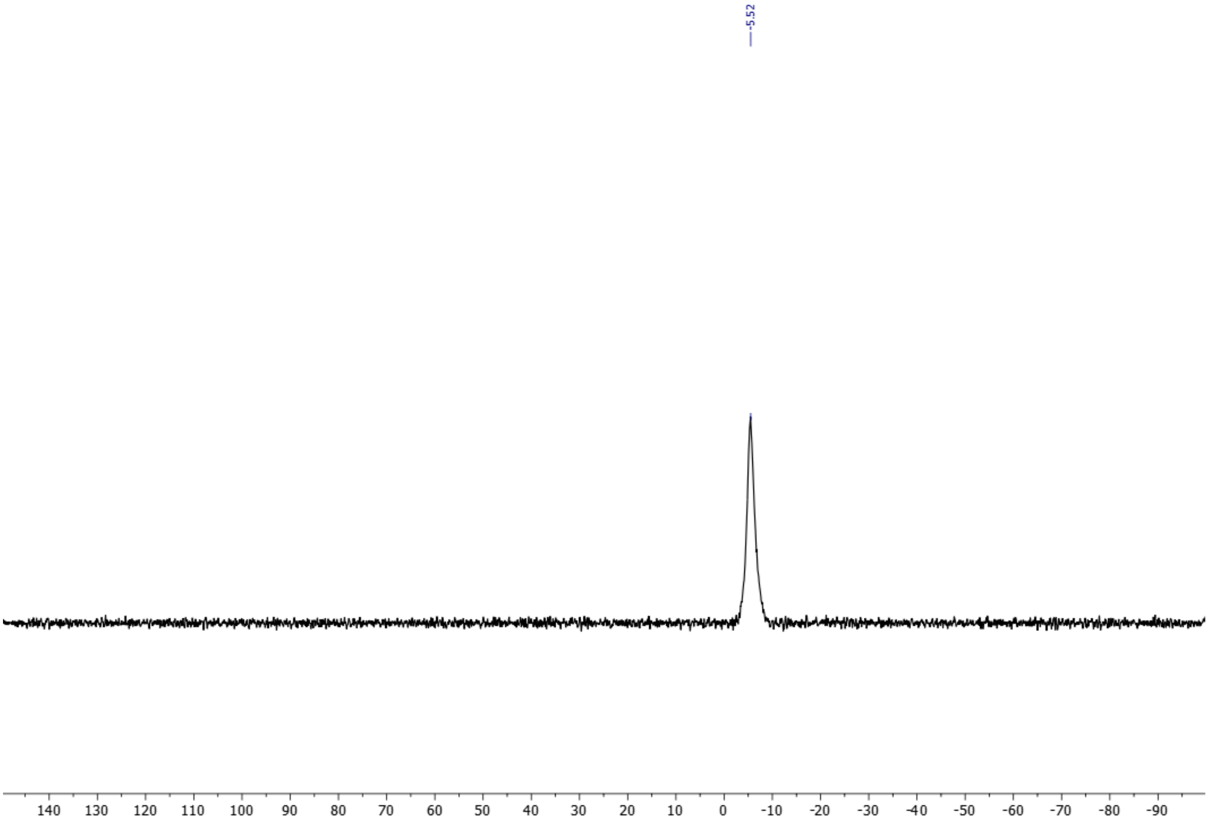

Figure S24. $^{11}B\{^{1}H\}$ NMR (128 MHz) spectrum of **4a** in C_6D_6.

1.2.9 Synthesis of Au(IDipp)(CPN$_3$Dipp)(B(C$_6$F$_6$)$_3$) (4c)

Au(IDipp)(CPN$_3$^{t}Bu) (20 mg, 0.03 mmol) and $B(C_6F_5)_3$ (14 mg, 0.03 mmol) were dissolved in toluene (0.5 mL) and stirred for 1 h. The solvent was removed *in vacuo* and the residue extracted into DCM (1 mL) and filtered. Storage at −35 ºC over 7 days yielded colorless crystals which were isolated by filtration, washed with pentane (3 × 0.5 mL), then dried under vacuum. Yield: 13 mg, 0.0097 mmol, 41%. Anal. Calcd. (%) for $C_{58}H_{54}AuBF_{15}N_5P$: C, 51.80; H, 4.05; N, 5.21. Found: C, 50.88; H, 3.91; N, 5.90.

^{1}H NMR (600 MHz, C_6D_6): δ(ppm) 7.27 (t, $^{3}J_{H-H}$ = 7.8 Hz, 2H, Dipp Ar**H**), 7.04 (m, $^{3}J_{H-H}$ = 7.8 Hz, 5H, Dipp Ar**H**), 6.92 (d, $^{3}J_{H-H}$ = 7.8 Hz, 2H, Dipp Ar**H**), 6.27 (s, 2H, IDipp C**H**), 2.51 (b, 4H, Dipp C**H**(CH$_3$)$_2$), 2.27 (b, 2H, Dipp C**H**(CH$_3$)$_2$), 1.30 (b, 12H, Dipp CH(C**H**$_3$)$_2$), 1.10 (b, 6H, Dipp CH(C**H**$_3$)$_2$), 0.95 (d, $^{3}J_{H-H}$ = 6.9 Hz, 12H, Dipp CH(C**H**$_3$)$_2$), 0.91 (b, 6H, Dipp CH(C**H**$_3$)$_2$).

$^{13}C\{^{1}H\}$ NMR (151 MHz, C_6D_6): δ(ppm) 210.38 (d, $^{1}J_{C-P}$ = 80.3 Hz, **C**PN$_3$^{Dipp}), 190.40 (d, $^{3}J_{C-P}$ = 2.2 Hz, IDipp {HCN(Dipp)}$_2$**C**Au), 149.64 (b, B(**C**$_6$F$_5$)), 148.04 (b, B(**C**$_6$F$_5$)), 145.88 (Dipp Ar-**C**), 145.58 (Dipp Ar-**C**), 140.75 (b, B(**C**$_6$F$_5$)), 139.11 (b, B(**C**$_6$F$_5$)), 138.03 (b, B(**C**$_6$F$_5$)), 136.40 (b, B(**C**$_6$F$_5$)), 134.94 (d, $^{3}J_{C-P}$ = 3.8 Hz, Dipp Ar**C**N) 134.43 (Dipp Ar-**C**), 131.02 (Dipp Ar-**C**), 124.30 (Dipp Ar-**C**), 124.21 (Dipp Ar-**C**), 123.85 (IDipp **C**H), 29.00 (Dipp **C**H(CH$_3$)$_2$),

28.43 (Dipp **C**H(CH_3)$_2$), 24.69 (Dipp CH(**C**H_3)$_2$), 24.55 (Dipp CH(**C**H_3)$_2$), 23.79 (Dipp CH(**C**H_3)$_2$), 23.69 (Dipp CH(**C**H_3)$_2$).

One of the Dipp Ar-**C** peaks could not be located.

$^{31}P\{^1H\}$ NMR (162 MHz, C_6D_6): δ(ppm) 220.4 ppm (s, C**P**N_3Dipp).

$^{19}F\{^1H\}$ NMR (377 MHz, C_6D_6): δ(ppm) −124.41 to −167.21 (m, B(C_6**F**$_5$)$_3$).

$^{11}B\{^1H\}$ NMR (128 MHz, C_6D_6): δ(ppm) −5.02 ppm (b, **B**(C_6F_5)$_3$).

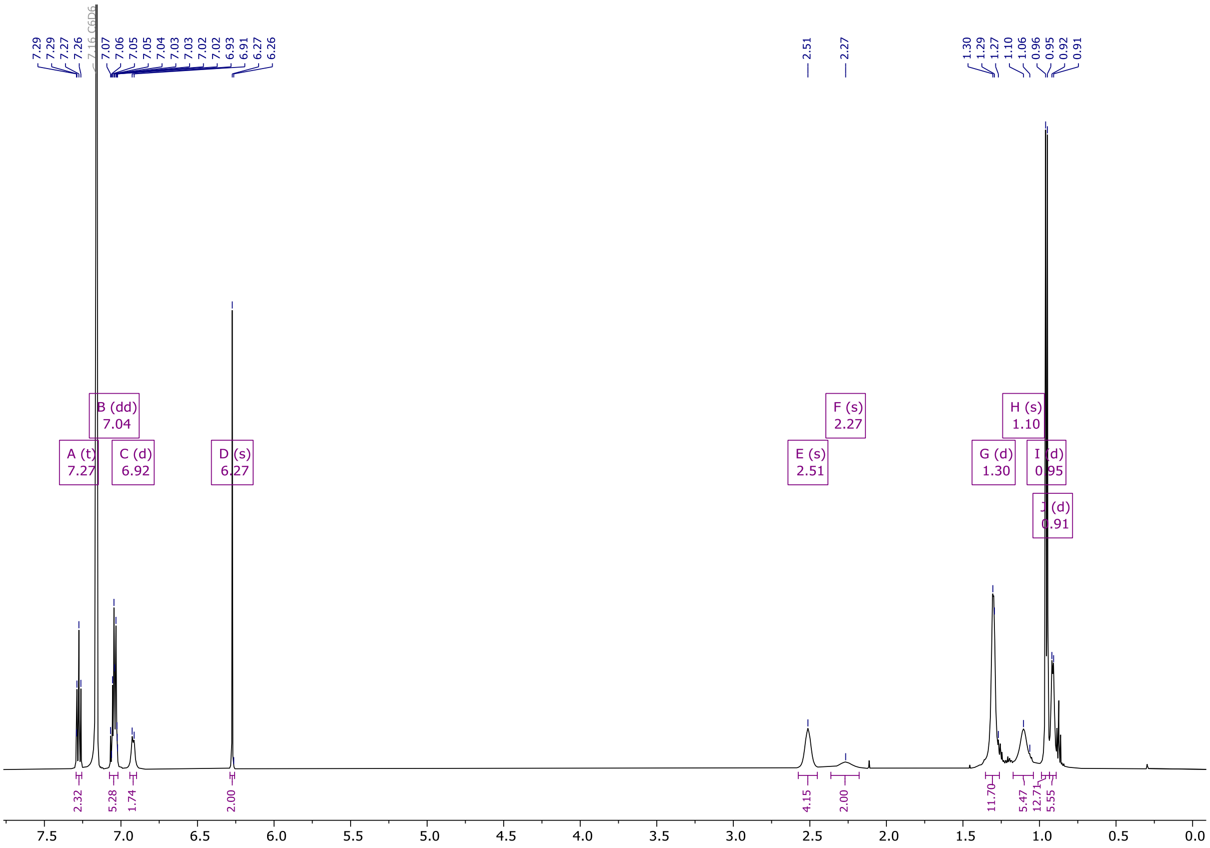

Figure S25. ^1H NMR (400 MHz) spectrum of **4c** in C_6D_6.

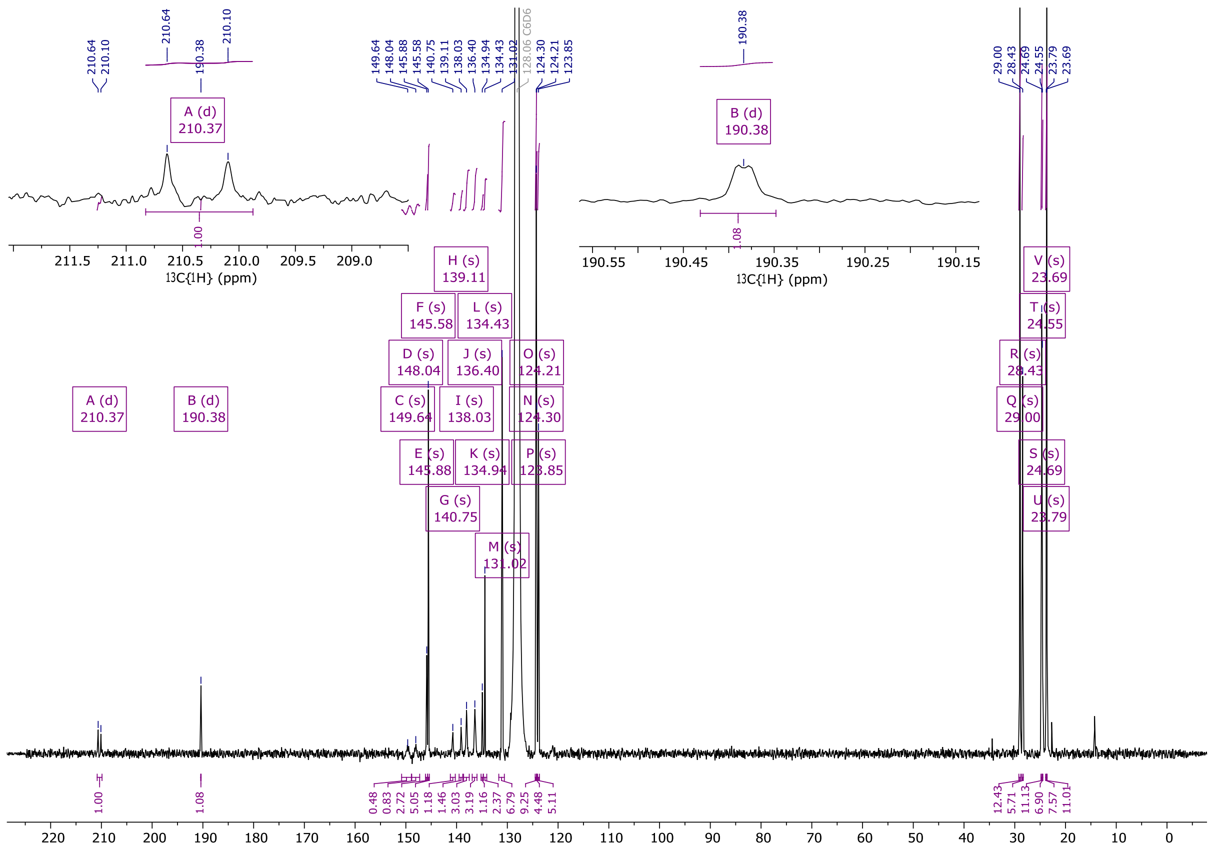

Figure S26. $^{13}C\{^{1}H\}$ NMR (151 MHz) spectrum of **4c** in C_6D_6.

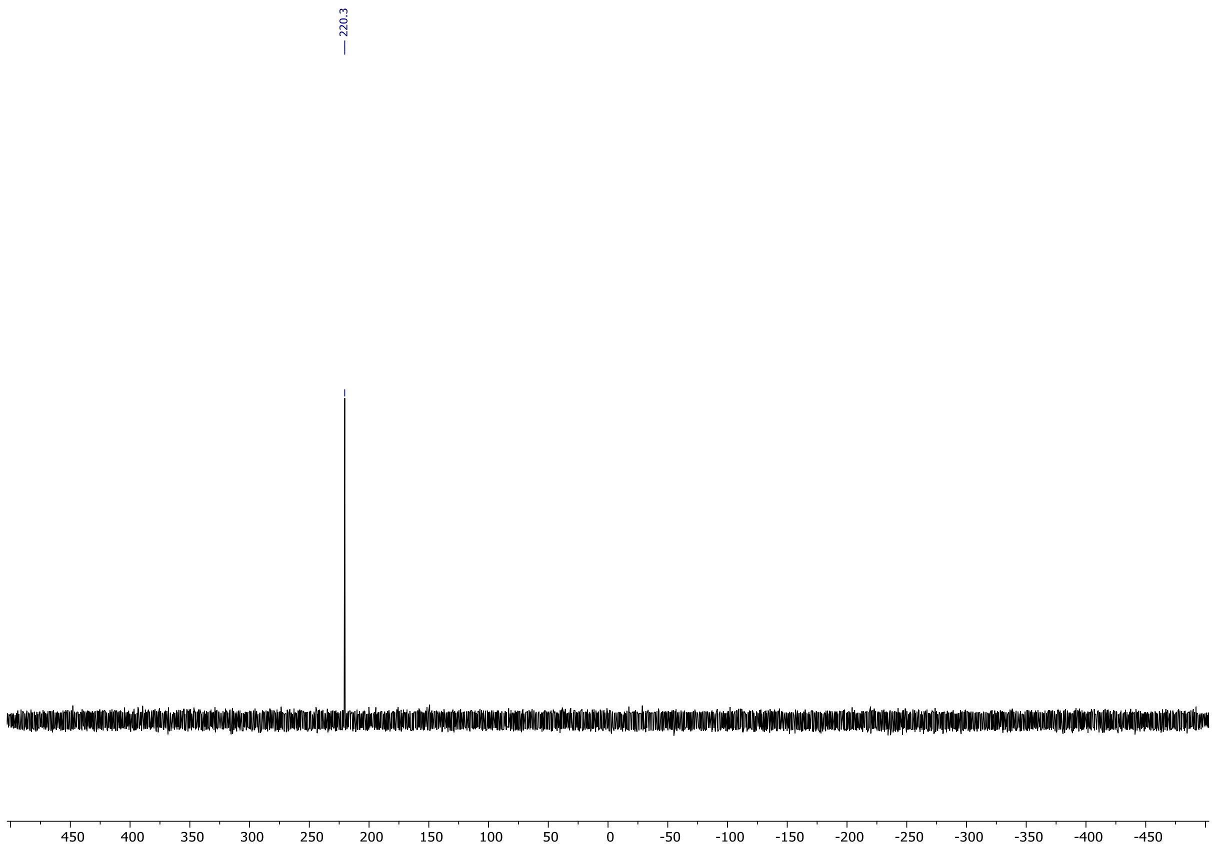

Figure S27. $^{31}P\{^{1}H\}$ NMR (162 MHz) spectrum of **4c** in C_6D_6.

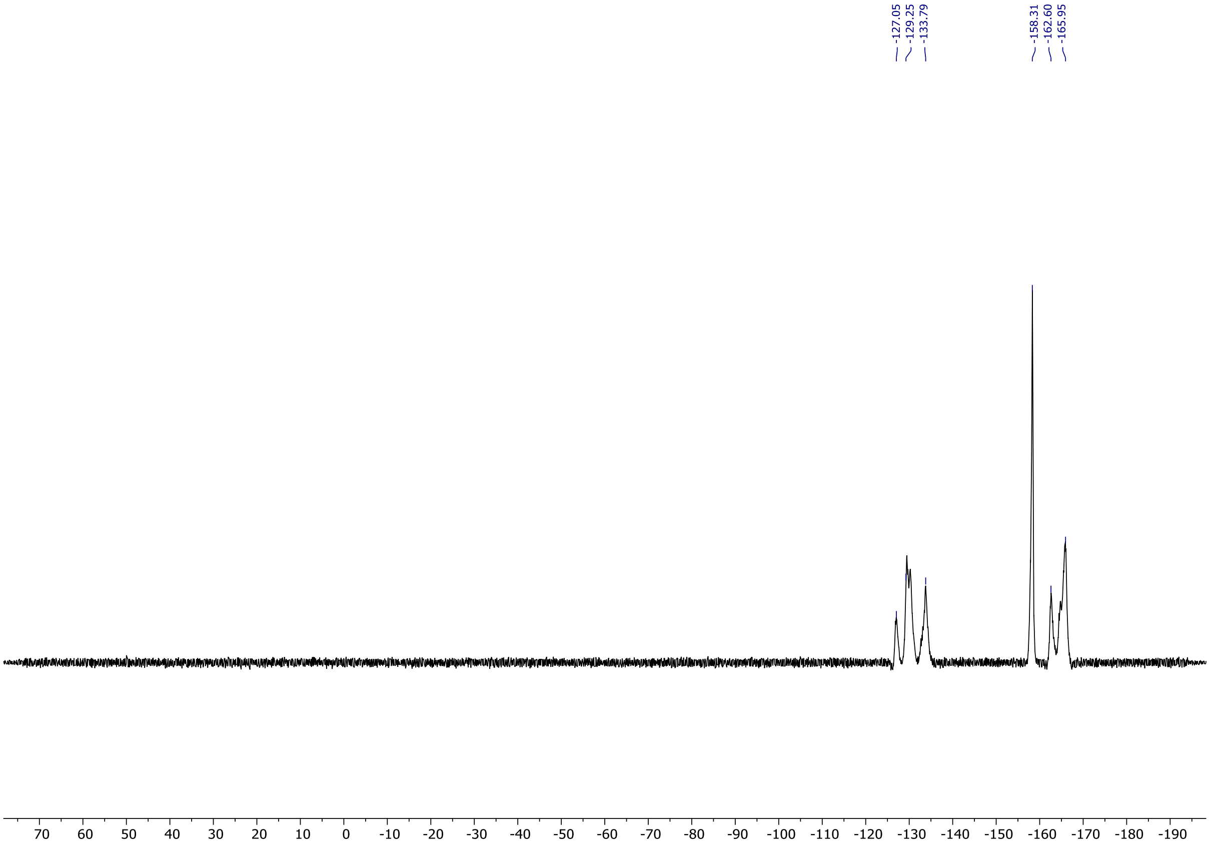

Figure S28. $^{19}F\{^{1}H\}$ NMR (377 MHz) spectrum of **4c** in C_6D_6.

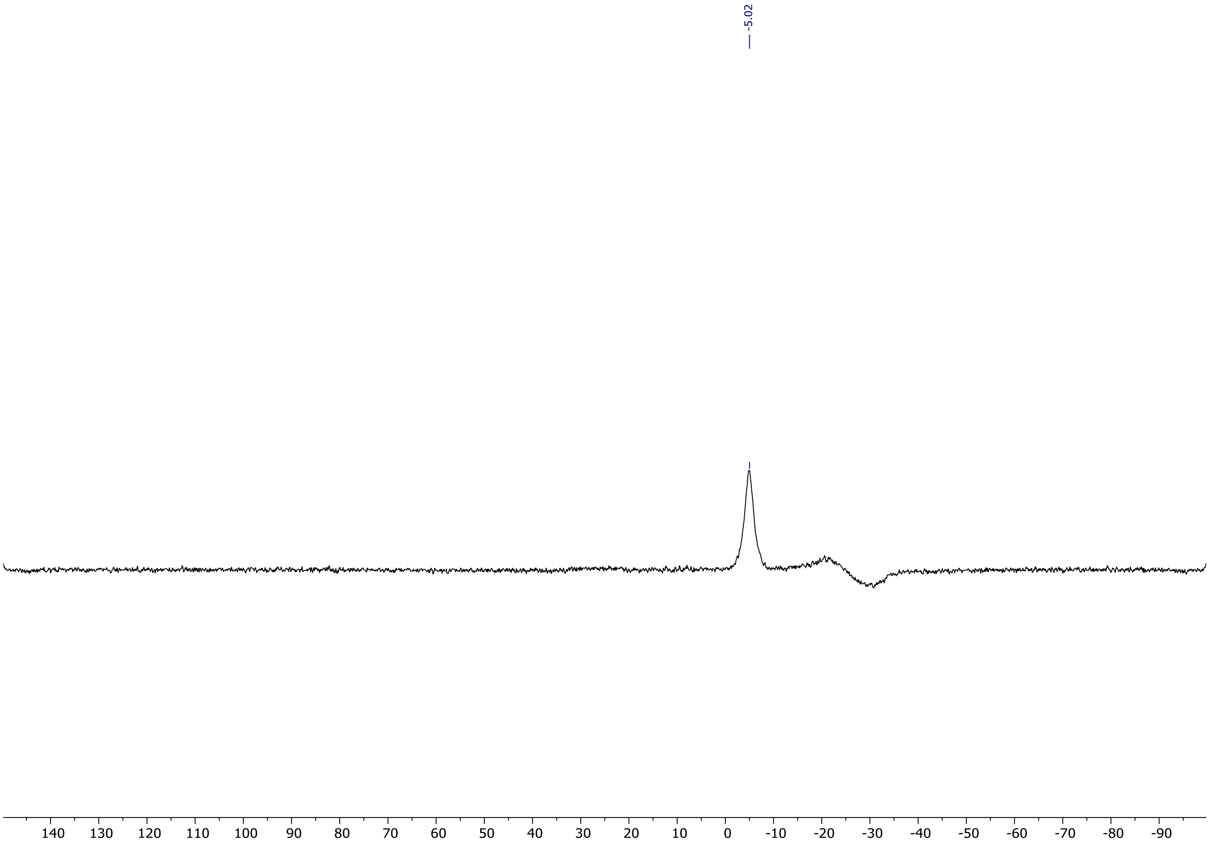

Figure S29. $^{11}B\{^{1}H\}$ NMR (128 MHz) spectrum of **4c** in C_6D_6.

1.2.10 Synthesis of $HCPN_3{}^tBu$ (5)

$\{Mg(^{Dipp}NacNac)(CPN_3{}^tBu)\}_2$ (48 mg, 0.04 mmol) and pyridinium chloride (19 mg, 0.16 mmol) were suspended in C_6D_6 (0.5 mL). The reaction mixture was sonicated for 1 h, after which the volatiles were distilled by vacuum transfer to an NMR tube.

^{1}H NMR (600 MHz, C_6D_6): δ(ppm) 8.89 (d, $^2J_{H-P}$ = 54.6 Hz, 1H, **H**$CPN_3{}^tBu$), 1.35 (d, $^4J_{H-P}$ = 0.6 Hz, 9H, ^{t}Bu C(C**H**$_3$)$_3$).

^{13}C{^{1}H} NMR (151 MHz, C_6D_6): δ(ppm) 164.10 (d, $^1J_{C-P}$ = 51.7 Hz, **C**$PN_3{}^tBu$), 61.43 (d, $^2J_{C-P}$ = 7.2 Hz, ^{t}Bu **C**(CH$_3$)$_3$), 61.43 (d, $^3J_{C-P}$ = 6.4 Hz, ^{t}Bu C(**C**H$_3$)$_3$).

^{31}P NMR (162 MHz, C_6D_6): δ(ppm) 174.9 (m, $^2J_{P-H}$ = 55.0 Hz, HC**P**$N_3{}^tBu$).

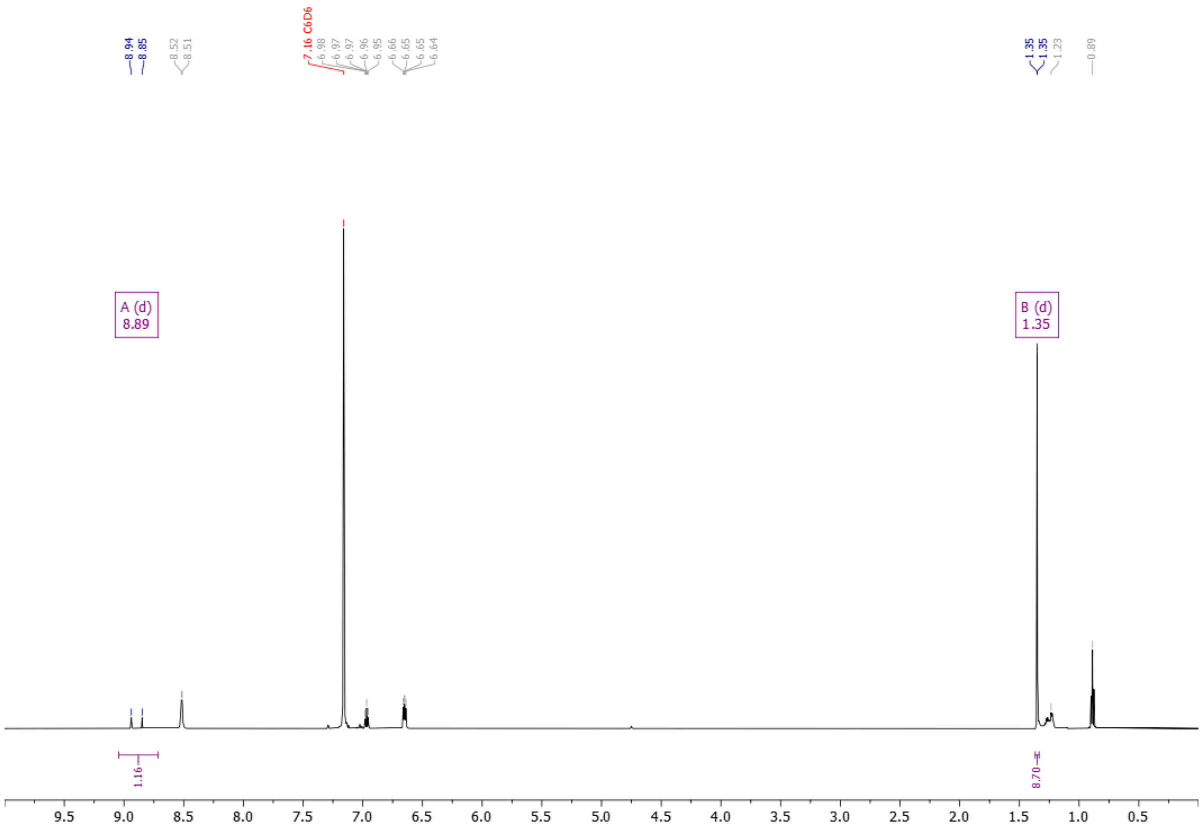

Figure S30. ^{1}H NMR (600 MHz) spectrum of **5** in C_6D_6.

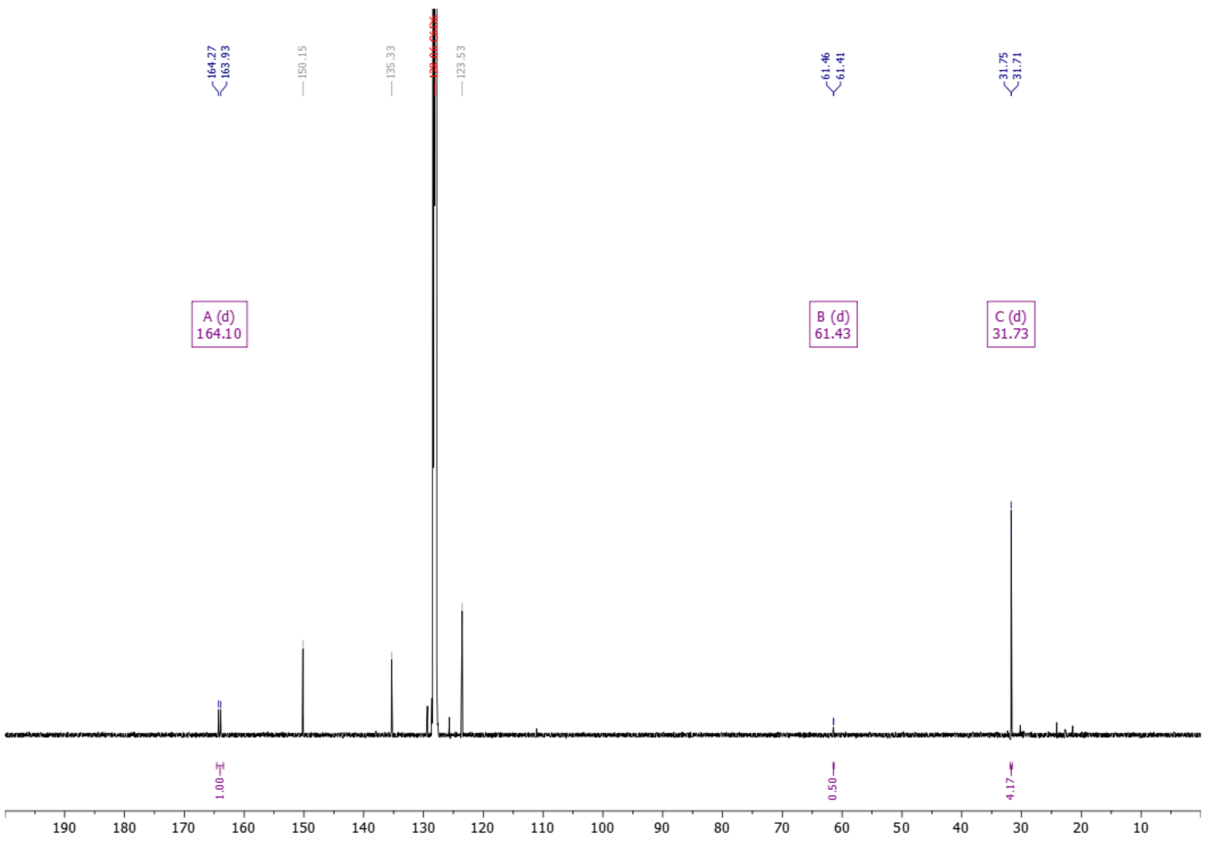

Figure S31. $^{13}C\{^1H\}$ NMR (151 MHz) spectrum of **5** in C_6D_6.

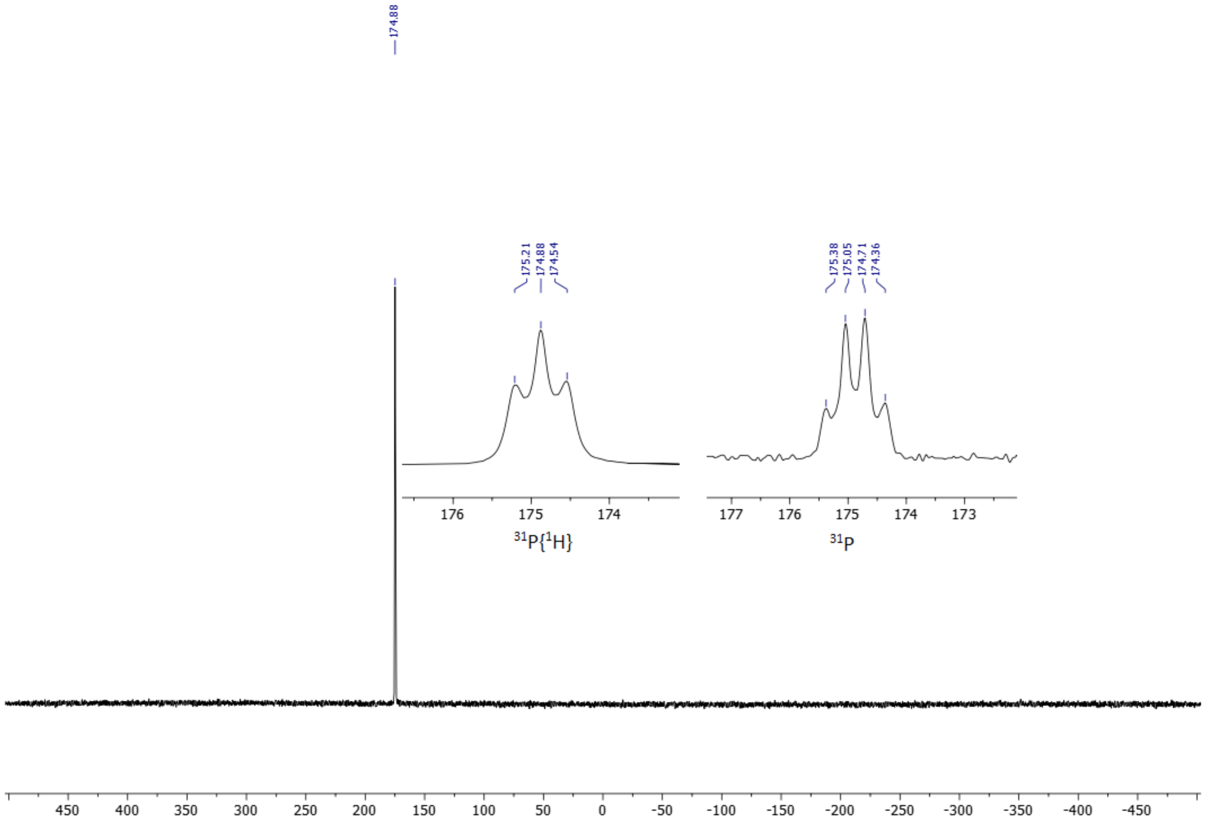

Figure S32. $^{31}P\{^1H\}$ and ^{31}P NMR (162 MHz) spectra of **5** in C_6D_6.

1.2.11 Synthesis of ICPN$_3$^{t}Bu (6a)

A solution of iodine in hexane (4 mL, 0.016 M, 0.06 mmol) was added dropwise to a stirred solution of Au(IDipp)(CPN$_3$^{t}Bu) (47 mg, 0.06 mmol) in toluene (5 mL). After stirring for 4 h, all volatiles were removed under vacuum. The residue was extracted with hexane (2 × 3 mL), which was then evaporated to dryness under vacuum to afford the product as an off-white powder. Crystals suitable for single crystal X-ray diffraction were obtained from hexane at −35 °C. Yield: 10 mg, 0.04 mmol, 58%. Anal. Calcd. (%) for $C_5H_9N_3IP$: C, 22.32; H, 3.37; N, 15.62. Found: C, 20.97; H, 2.67; N, 14.89.

^{1}H NMR (600 MHz, C_6D_6): δ(ppm) 1.18 (d, $^4J_{H\text{-}P}$ = 0.9 Hz, 9H, ^{t}Bu C(C**H**$_3$)$_3$).

^{13}C{^{1}H} NMR (151 MHz, C_6D_6): δ(ppm) 120.80 (d, $^1J_{C\text{-}P}$ = 80.6 Hz, I**C**PN$_3$^{t}Bu), 62.26 (d, $^2J_{C\text{-}P}$ = 6.0 Hz, ^{t}Bu **C**(CH$_3$)$_3$), 31.27 (d, $^3J_{C\text{-}P}$ = 6.4 Hz, ^{t}Bu C(**C**H$_3$)$_3$).

^{31}P{^{1}H} NMR (162 MHz, C_6D_6): δ(ppm) 179.9 (m, IC**P**N$_3$^{t}Bu).

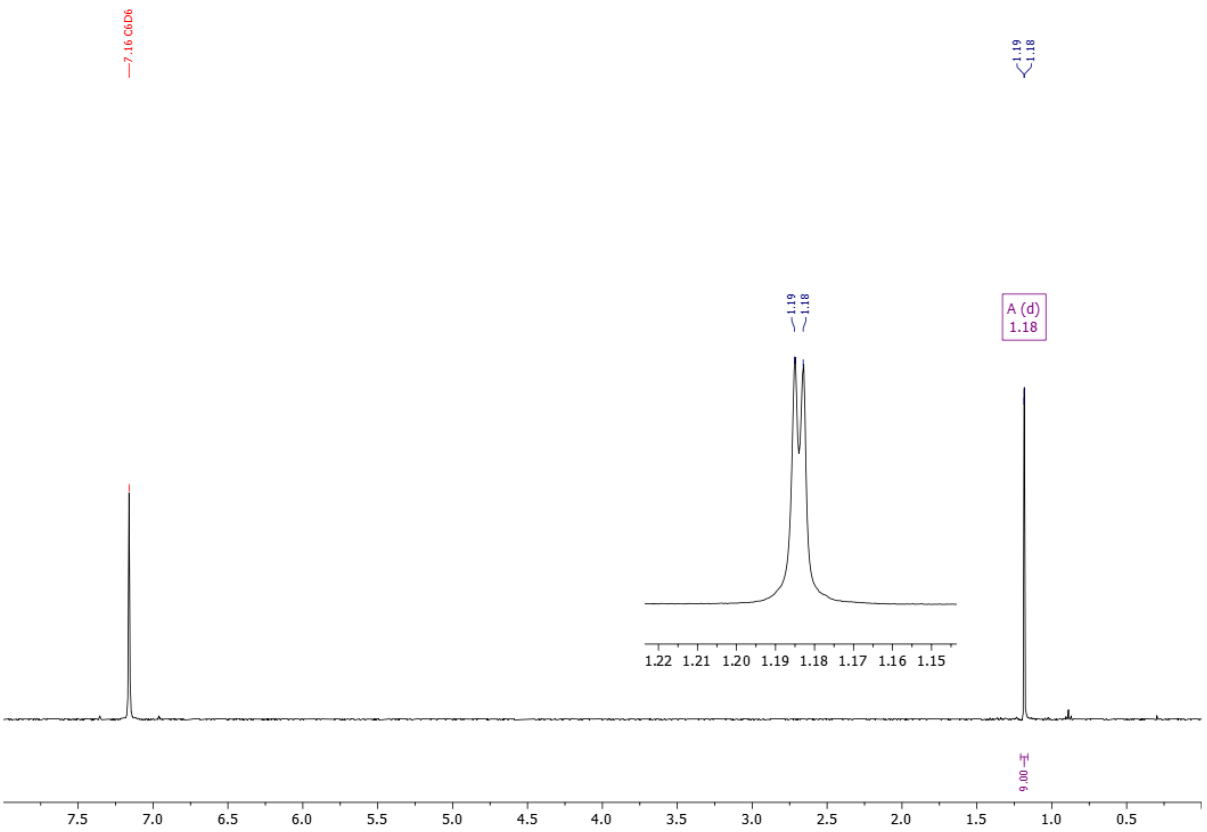

Figure S33. ^{1}H NMR (400 MHz) spectrum of **6a** in C_6D_6.

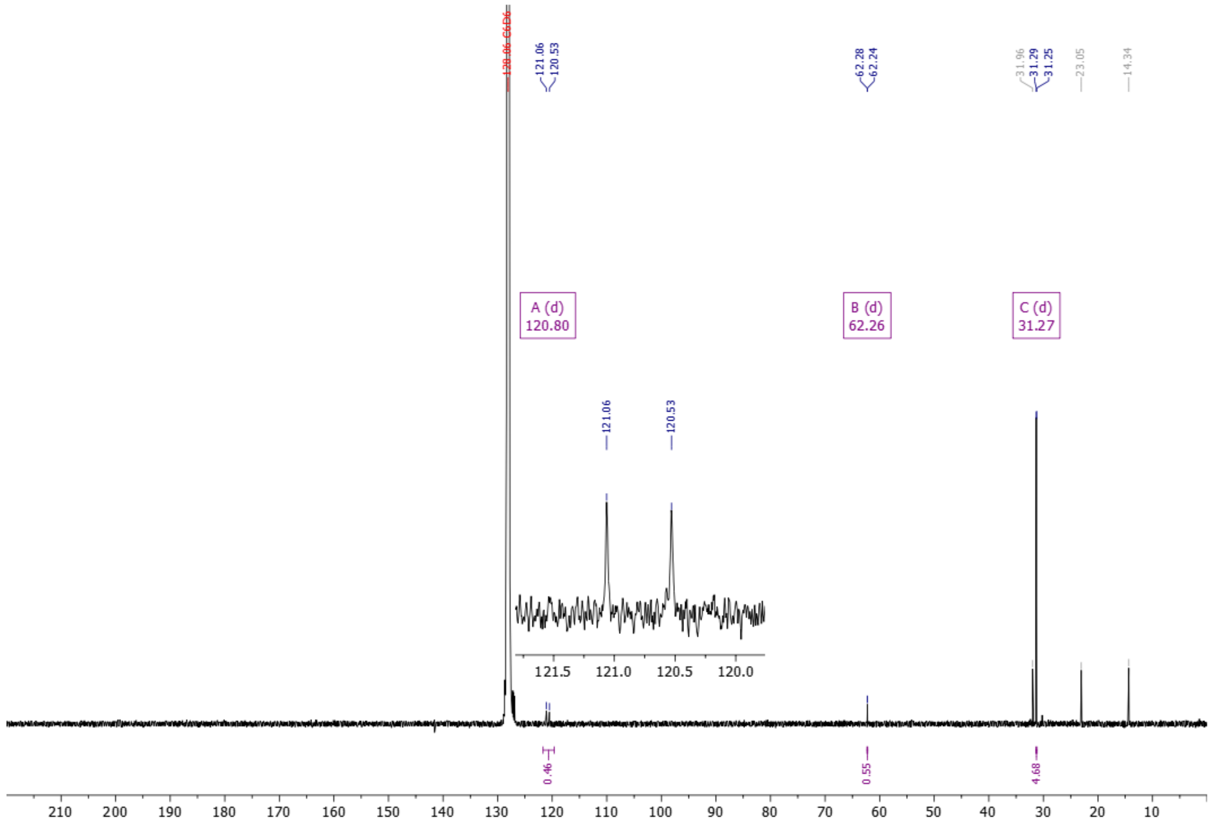

Figure S34. $^{13}C\{^{1}H\}$ NMR (151 MHz) spectrum of **6a** in C_6D_6.

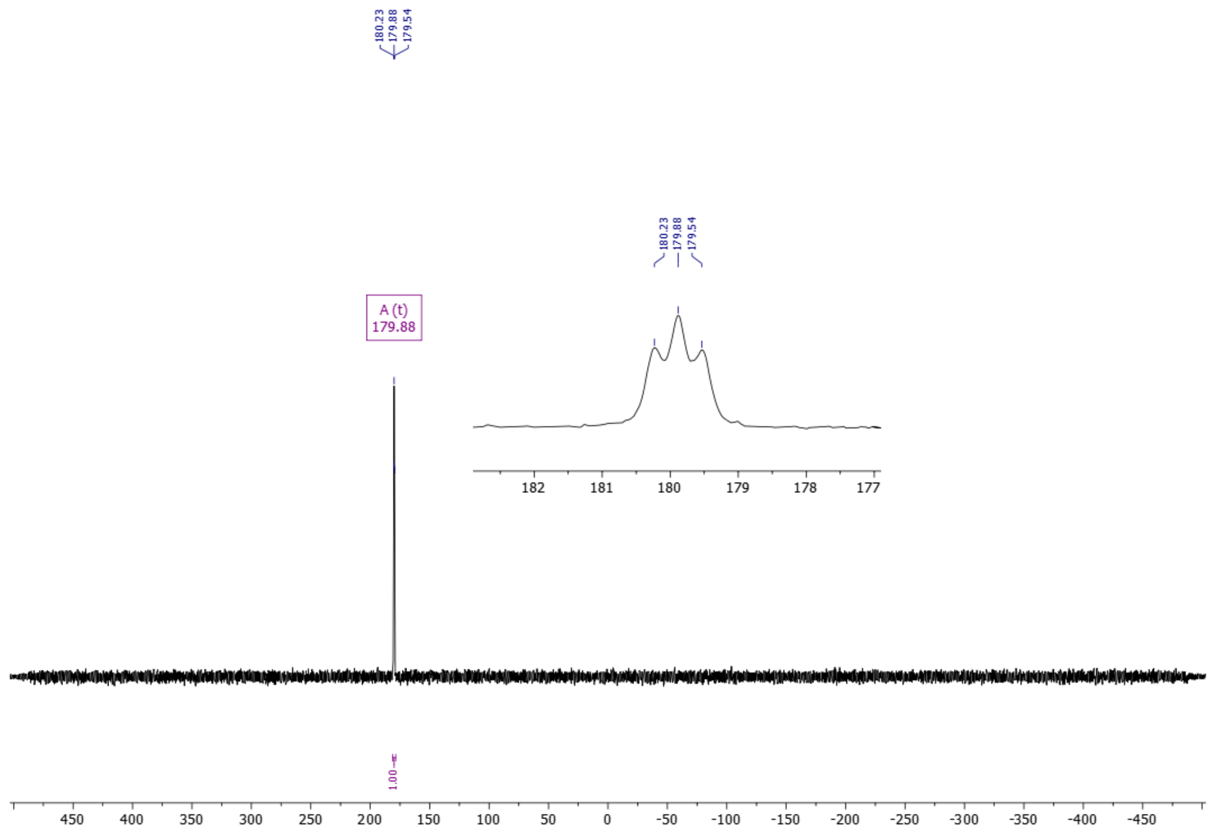

Figure S35. $^{31}P\{^{1}H\}$ NMR (162 MHz) spectrum of **6a** in C_6D_6.

1.2.12 Synthesis of ICPN$_3$Ad (6b)

A solution of iodine in hexane (0.8 mL, 0.1 M, 0.08 mmol) was added to a solution of Au(IDipp)(CPN$_3$Ad) (64 mg, 0.08 mmol) in toluene (1 mL), which was then stirred overnight. All volatiles were removed under vacuum, and the resulting residue was extracted with hexane (2 × 3 mL). The hexane solution was evaporated to dryness, yielding the product as a colorless solid. Yield: 16 mg, 0.05 mmol, 58%. Anal. Calcd. (%) for $C_{11}H_{15}N_3IP\cdot0.5(C_6H_6)$: C, 43.53; H, 4.70; N, 10.88. Found: C, 42.96; H, 4.73; N, 10.77.

^{1}H NMR (400 MHz, C_6D_6): δ(ppm) 1.96–1.91 (m, 6H, Ad C{C**H**$_2$CHCH$_2$}$_3$), 1.81–1.75 (m, 3H, Ad C{CH$_2$C**H**CH$_2$}$_3$), 1.41–1.31 (m, 6H, Ad C{CH$_2$CHC**H**$_2$}$_3$).

^{13}C{^{1}H} NMR (151 MHz, C_6D_6): δ(ppm) 120.50 (d, $^1J_{C–P}$ = 80.8 Hz, I**C**PN$_3$Ad), 62.90 (d, $^2J_{C–P}$ = 5.3 Hz, Ad **C**{CH$_2$CHCH$_2$}$_3$), 45.16 (d, $^3J_{C–P}$ = 6.5 Hz, Ad C{**C**H$_2$CHCH$_2$}$_3$), 35.93 (Ad C{CH$_2$CH**C**H$_2$}$_3$), 30.06 (d, $^4J_{C–P}$ = 0.7 Hz, Ad C{CH$_2$**C**HCH$_2$}$_3$).

^{31}P{^{1}H} NMR (162 MHz, C_6D_6): δ(ppm) 180.1 (m, IC**P**N$_3$Ad).

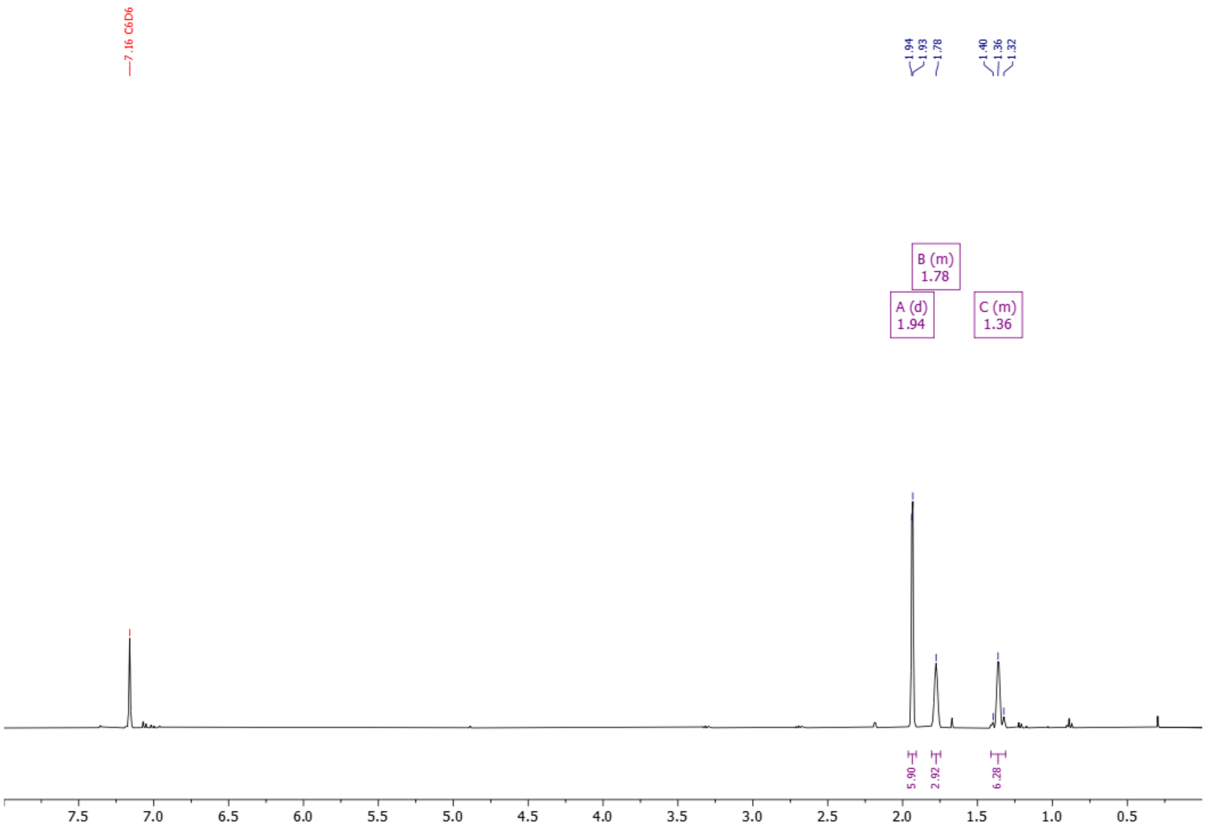

Figure S36. ^{1}H NMR (400 MHz) spectrum of **6b** in C_6D_6.

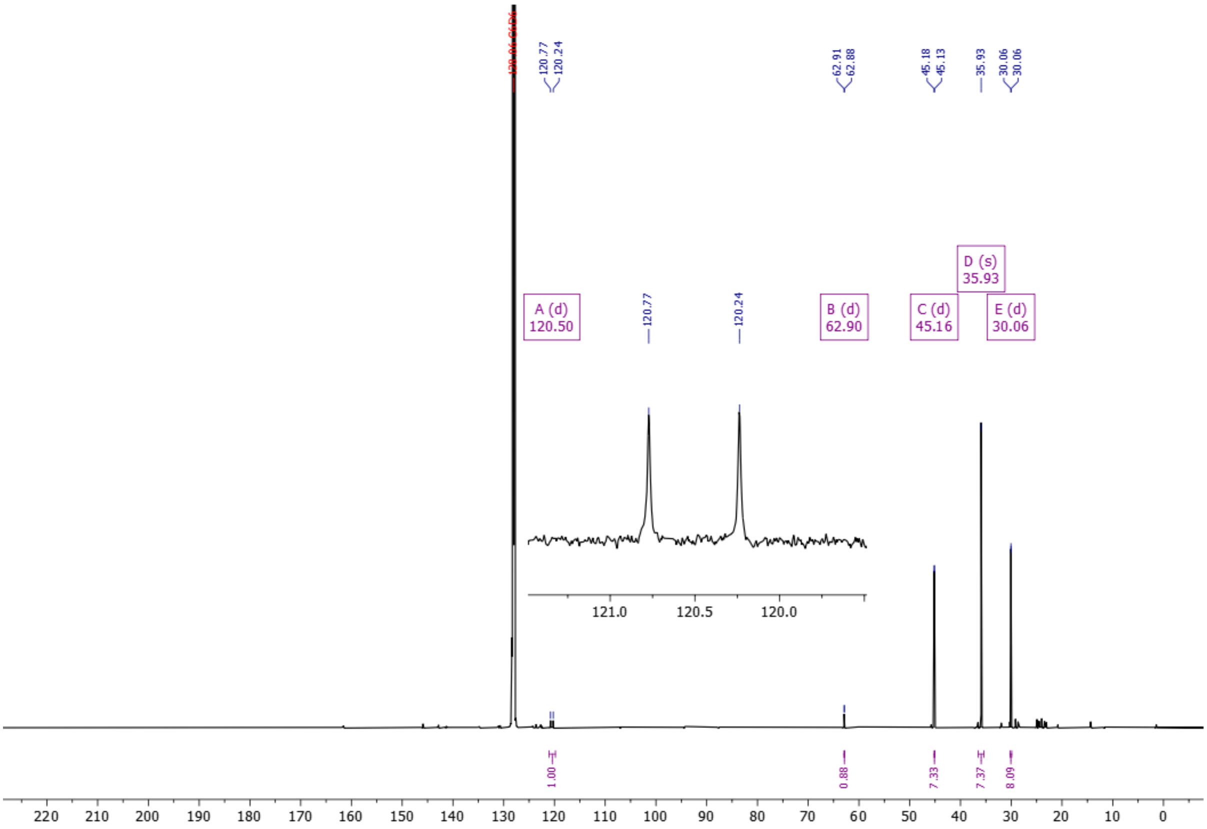

Figure S37. $^{13}C\{^{1}H\}$ NMR (151 MHz) spectrum of **6b** in C_6D_6.

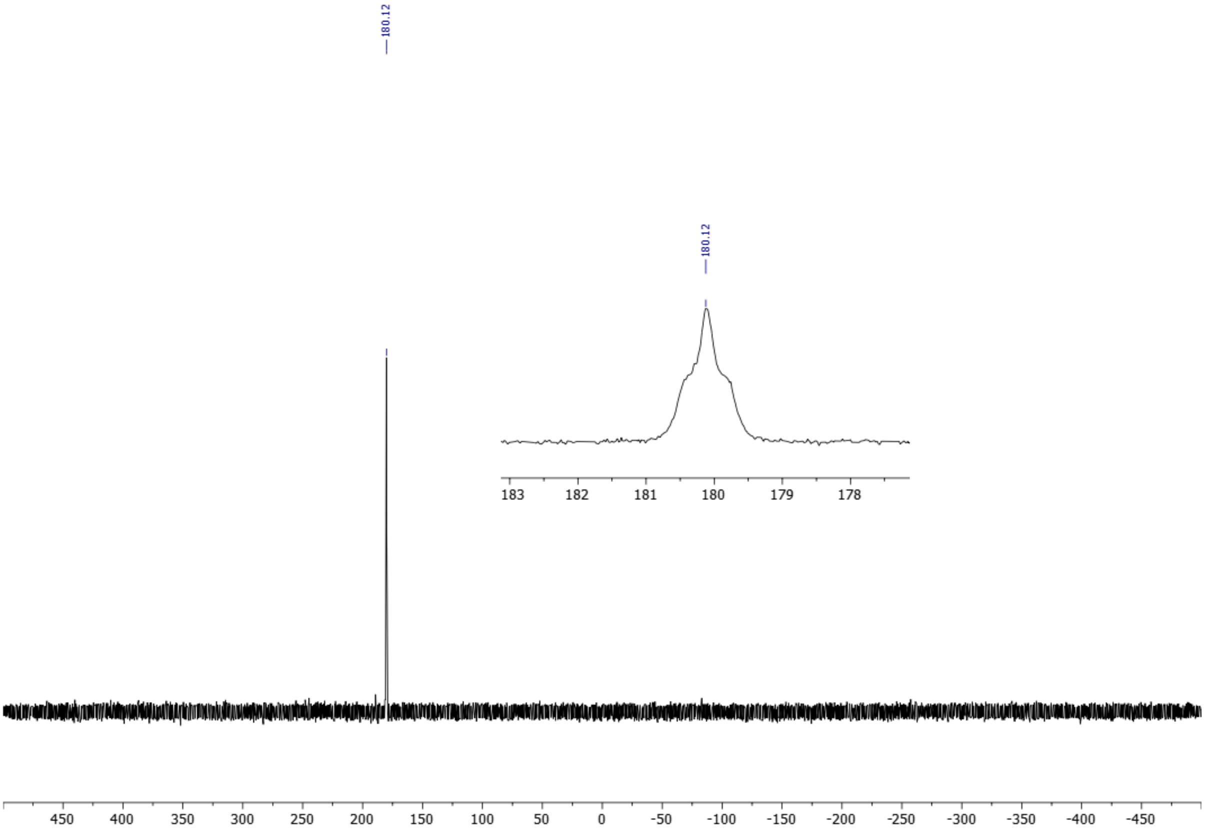

Figure S38. $^{31}P\{^{1}H\}$ NMR (162 MHz) spectrum of **6b** in C_6D_6.

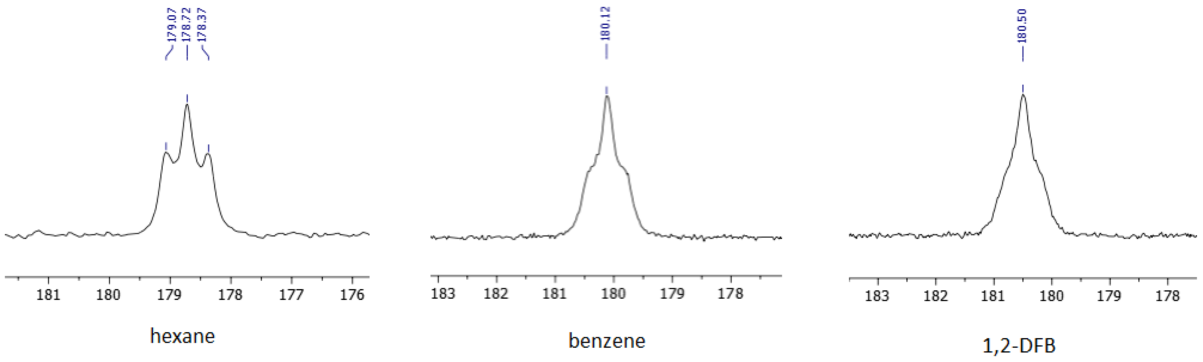

Figure S39. $^{31}P\{^{1}H\}$ NMR (162 MHz) spectra of **6b** run unlocked in hexane, benzene, and 1,2-DFB.

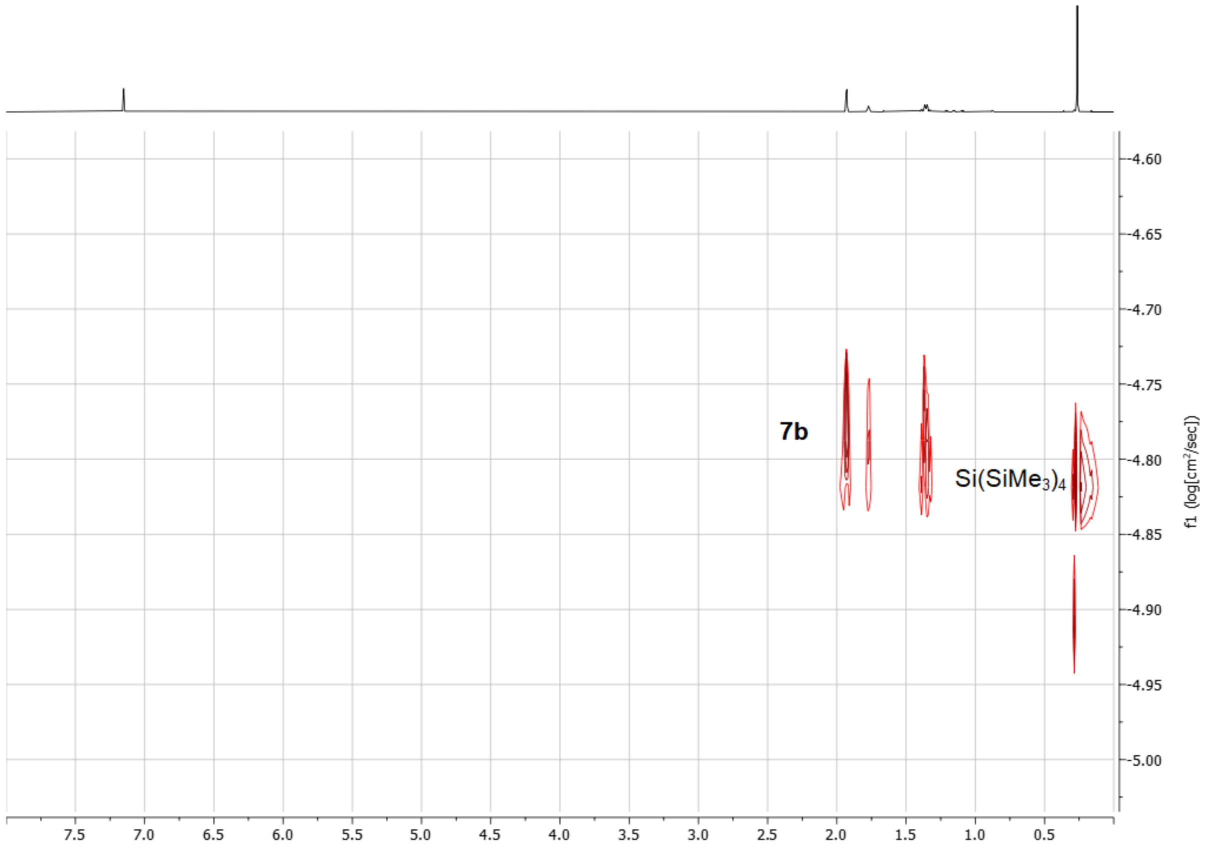

Figure S40. Convection compensated ^{1}H DOSY spectrum (500 MHz) of **6b** in benzene, using $Si(SiMe_3)_4$ as an internal standard.

Table S2. Diffusion coefficients and calculated hydrodynamic radii of **6b** and $Si(SiMe_3)_4$.

	**6b**	$Si(SiMe_3)_4$
D / m^2 sec^{-1}	1.5×10^{-9}	1.6×10^{-9}
R_h (calc) / Å	4.7	4.3
$D_{Si(SiMe3)4}/D_{\mathbf{6b}}$	**1.1**	
$R_{h\mathbf{6b}}/R_{hSi(SiMe3)4}$	**1.1**	

1.2.13 Synthesis of $ICPN_3Dipp$ (6c)

A solution of iodine in hexane (0.8 mL, 0.1 M, 0.08 mmol) was added via syringe to a solution of Au(IDipp)(CPN_3Dipp) (67 mg, 0.08 mmol) in toluene (5 mL). The solution was stirred overnight at room temperature. All volatiles were removed under vacuum, and the resulting residue was extracted with hexane (2 × 3 mL). The hexane solution was evaporated to dryness, yielding the product as a dark red oil. Yield: 22 mg, mmol, 76%. Anal. Calcd. (%) for $C_{13}H_{17}N_3IP$: C, 41.84; H, 4.59; N, 11.26. Found: C, 46.36; H, 5.25; N, 10.56.

^{1}H NMR (400 MHz, C_6D_6): δ(ppm) 6.98 (d, 2H, $^3J_{H-H}$ = 7.8 Hz, Dipp *meta* C**H**), 2.10 (sept, $^3J_{H-H}$ = 6.8 Hz, 2H, Dipp C**H**(CH_3)$_2$, 0.91 (d, $^3J_{H-H}$ = 6.8 Hz, 6H, Dipp CH(C**H**$_3$)$_2$, 0.86 (d, $^3J_{H-H}$ = 6.9 Hz, 6H, Dipp CH(C**H**$_3$)$_2$).

^{13}C{^{1}H} NMR (151 MHz, C_6D_6): δ(ppm) 146.26 (d, $^3J_{C-P}$ = 1.6 Hz, *ortho* Dipp **C**), 134.78 (d, $^2J_{C-P}$ = 6.3 Hz, Ar**C**N), 130.87 (Dipp **C**), 128.35 (Ar**C**), 124.08 (Ar**C**), 123.36 (d, $^1J_{C-P}$ = 83.5 Hz, **C**PN$_3$Ad), 28.69 (Dipp **C**H(CH_3)$_2$), 24.30 (Dipp CH(**C**H$_3$)$_2$, 24.07 (Dipp CH(**C**H$_3$)$_2$).

^{31}P{^{1}H} NMR (162 MHz, C_6D_6): δ(ppm) 194.7 (IC**P**N$_3$Dipp).

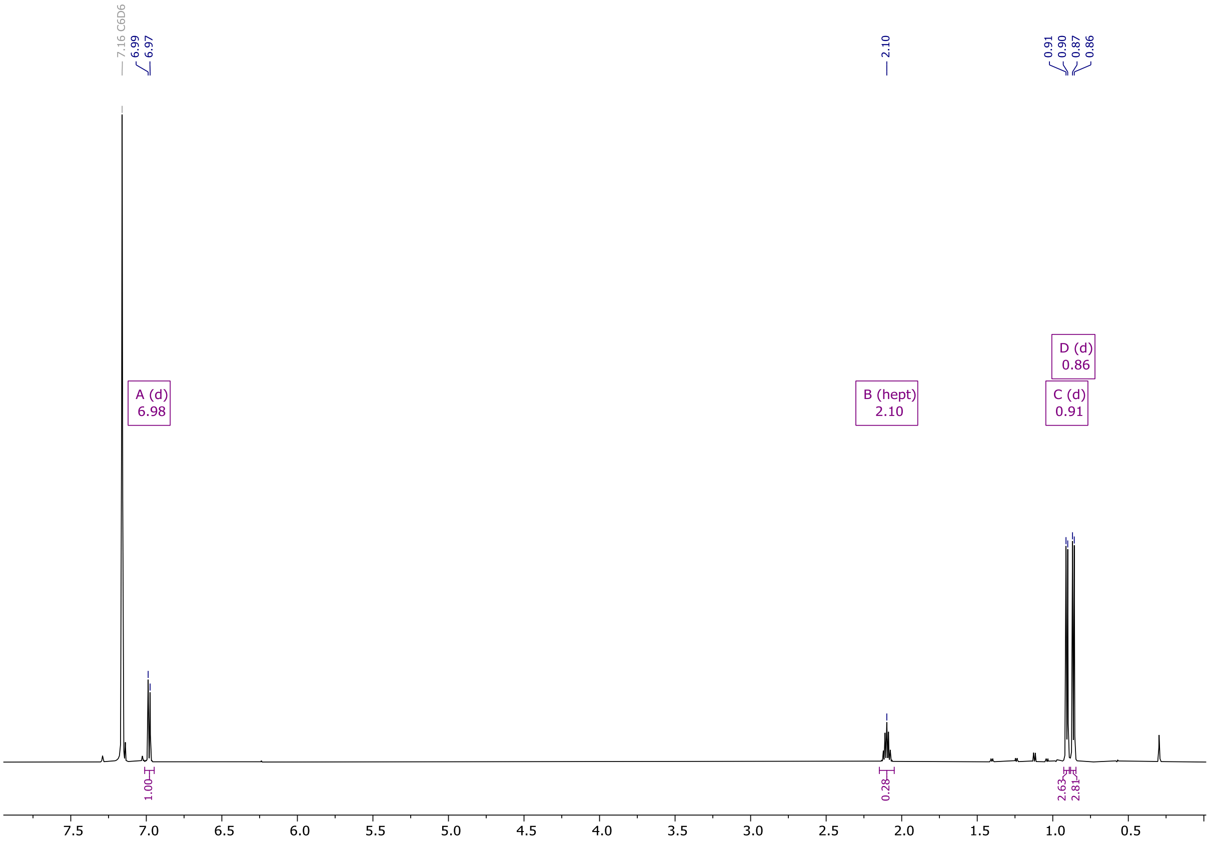

Figure S41. ^{1}H NMR (400 MHz) spectrum of **6c** in C_6D_6.

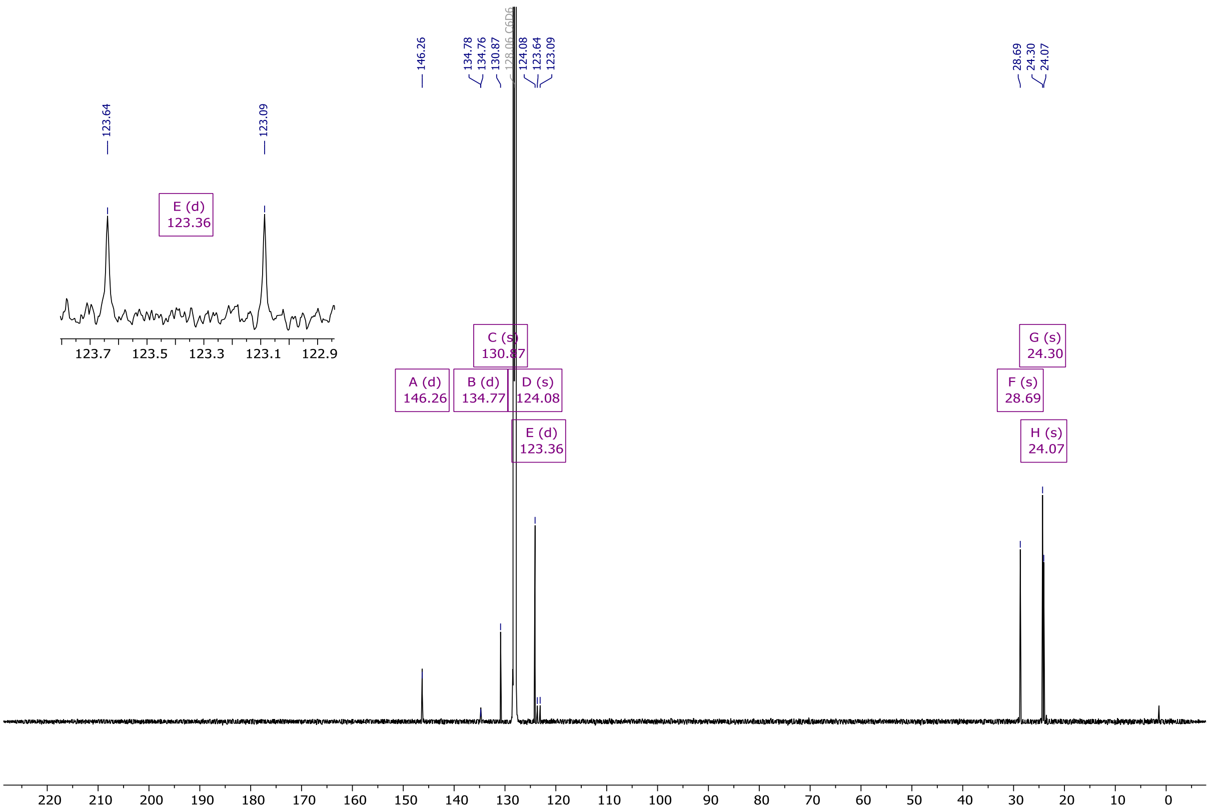

Figure S42. $^{13}C\{^1H\}$ NMR (151 MHz) spectrum of **6c** in C_6D_6.

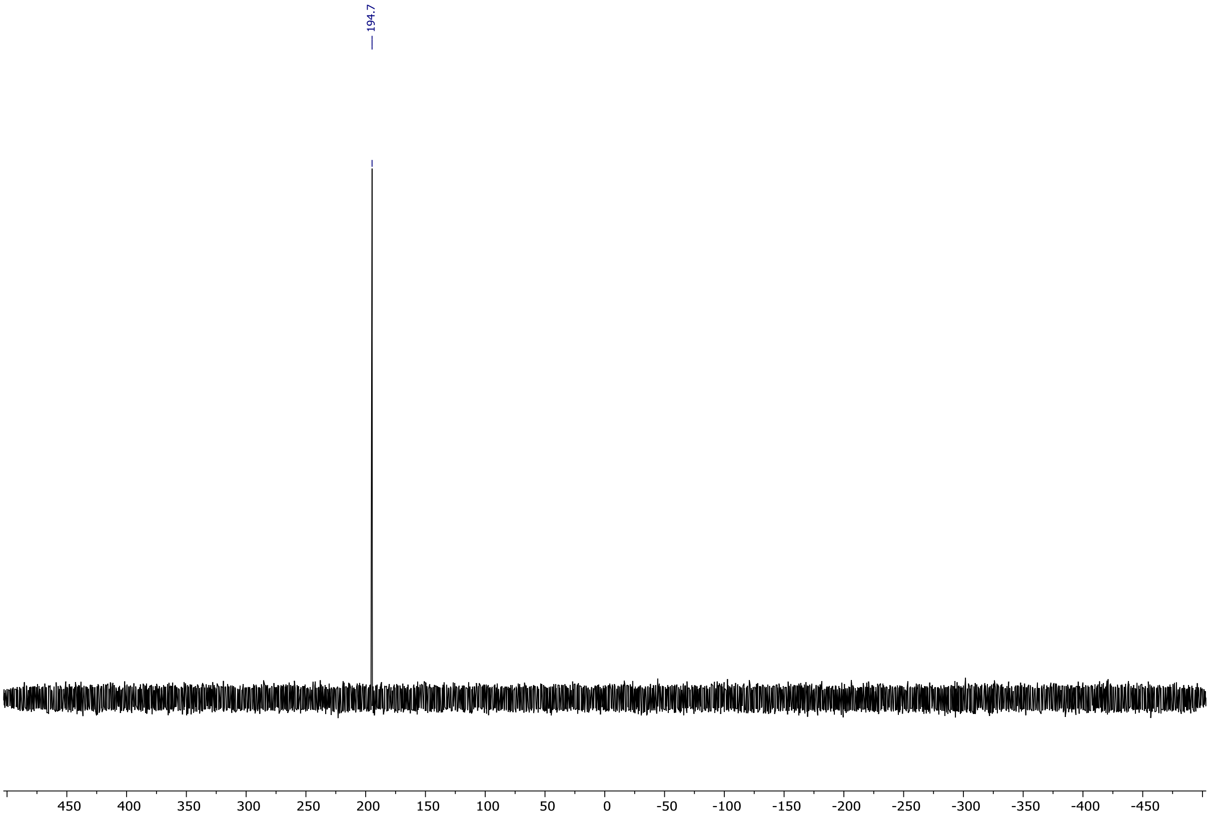

Figure S43. $^{31}P\{^1H\}$ NMR (162 MHz) spectrum of **6c** in C_6D_6.

2. Single crystal X-ray diffraction data

Single-crystal X-ray diffraction data were collected using either an Oxford Diffraction Supernova dual-source diffractometer equipped with a 135 mm Atlas CCD area detector, or a Rigaku XtaLAB Synergy R diffractometer equipped with a HyPix-Arc 150 detector. Crystals were selected under Paratone-N oil, mounted on micromount loops and quench-cooled using an Oxford Cryosystems open flow N_2 cooling device. Data were collected using mirror monochromated Cu Kα (λ = 1.54184 Å) radiation and processed using the CrysAlisPro package, including unit cell parameter refinement and inter-frame scaling (which was carried out using SCALE3 ABSPACK within CrysAlisPro).[38] Structures were subsequently solved using direct methods and refined on F^2 using the SHELXL package.[39]

Table S3. Selected X-ray data collection/refinement parameters for **1a**·C_6H_6, **1b**·tol and **1c**·C_6H_6.

	**1a**·C_6H_6	**1b**·tol	**1c**·C_6H_6
Formula	$C_{38}H_{51}AuN_5P$	$C_{45}H_{59}AuN_5P$	$C_{46}H_{59}AuN_5P$
CCDC	2263738	2263730	2263731
Fw [g mol^{-1}]	805.77	897.90	909.91
Crystal system	orthorhombic	monoclinic	monoclinic
Space group	P*nma*	*I*2/*a*	*P*2$_1$/*c*
a (Å)	16.2045(1)	20.7310(1)	12.5798(2)
b (Å)	10.6534(1)	16.1721(1)	16.7485(2)
c (Å)	23.8480(2)	26.0458(4)	22.2537(3)
α (°)	90	90	90
β (°)	90	102.725(1)	106.371(2)
γ (°)	90	90	90
V (Å^3)	4116.95(6)	8517.74(15)	4498.60(12)
Z	4	8	4
Radiation, λ (Å)	Cu Kα, 1.54184	Cu Kα, 1.54184	Cu Kα, 1.54184
Temp (K)	200(2)	150(2)	150(2)
ρ_{calc} (g cm^{-3})	1.300	1.400	1.343
μ (mm^{-1})	7.296	7.113	6.742
Reflections collected	48427	100264	88948
Indep. reflections	4547	8876	9366
Parameters	328	518	516
R(int)	0.0510	0.0394	0.0755
R1/wR2,[a] I ≥ 2σI (%)	4.29/12.04	1.81/4.27	3.62/8.90
R1/wR2,[a] all data (%)	4.48/12.14	2.05/4.39	4.33/9.44
GOF	1.270	1.044	1.062

[a] $R1 = [\Sigma||F_o| - |F_c||]/\Sigma|F_o|$; $wR2 = \{[\Sigma w[(F_o)^2 - (F_c)^2]^2]/[\Sigma w(F_o^2)^2]\}^{1/2}$; $w = [\sigma^2(F_o)^2 + (AP)^2 + BP]^{-1}$, where $P = [(F_o)^2 + 2(F_c)^2]/3$ and the A and B values are 0.0257 and 18.57 for **1a**·C_6H_6, 0.0193 and 9.81 for **1b**·tol, and 0.0399 and 9.14 for **1c**·C_6H_6.

Table S4. Selected X-ray data collection/refinement parameters for **1d**·tol, **2a** and **2b**·2tol.

	**1d**·tol	**2a**	**2b**·2tol
Formula	$C_{69}H_{84}Au_2N_{10}P_2$	$C_{68}H_{100}Mg_2N_{10}P_2$	$C_{81}H_{104}Mg_2N_{10}P_2$
CCDC	2263732	2263733	2263734
Fw [g mol^{-1}]	1509.33	1168.13	1328.30
Crystal system	triclinic	monoclinic	triclinic
Space group	*P*–1	*C*2/*m*	*P*–1
a (Å)	14.1752(4)	18.1532(2)	13.0652(5)
b (Å)	21.0565(4)	19.1427(2)	13.1025(6)
c (Å)	24.3488(6)	12.43930(10)	14.2674(7)
α (°)	94.882(2)	90	114.624(4)
β (°)	90.052(2)	122.232(1)	113.693(4)
γ (°)	101.648(2)	90	95.063(4)
V (Å^3)	7090.9(3)	3656.53(7)	1934.86(17)
Z	4	2	1
Radiation, λ (Å)	Cu Kα, 1.54184	Cu Kα, 1.54184	Cu Kα, 1.54184
Temp (K)	150(2)	150(2)	150(2)
ρ_{calc} (g cm^{-3})	1.414	1.061	1.140
μ (mm^{-1})	8.436	1.033	1.037
Reflections collected	72456	18582	18706
Indep. reflections	24975	3960	7813
Parameters	1526	199	459
R(int)	0.0726	0.0202	0.0167
R1/wR2,[a] I ≥ 2σI (%)	6.52/17.69	3.46/10.15	6.11/16.10
R1/wR2,[a] all data (%)	8.49/19.33	3.71/10.47	6.33/16.21
GOF	1.106	1.051	1.126

[a] $R1 = [\Sigma||F_o| - |F_c||]/\Sigma|F_o|$; $wR2 = \{[\Sigma w[(F_o)^2 - (F_c)^2]^2]/[\Sigma w(F_o^2)^2]\}^{1/2}$; $w = [\sigma^2(F_o)^2 + (AP)^2 + BP]^{-1}$, where $P = [(F_o)^2 + 2(F_c)^2]/3$ and the A and B values are 0.0975 and 15.88 for **1d**·tol, 0.0610 and 1.85 for **2a**, and 0.0364 and 3.66 for **2b**·2tol.

Table S5. Selected X-ray data collection/refinement parameters for **3**, **4a** and **4c**·$2CH_2Cl_2$

	**3**	**4a**	**4c**·$2CH_2Cl_2$
Formula	$C_{34}H_{50}GeN_5P$	$C_{50}H_{45}AuBF_{15}N_5P$	$C_{60}H_{57}AuBCl_4F_{15}N_5P$
CCDC	2263735	2263736	2263737
Fw [g mol^{-1}]	632.35	1239.65	1513.65
Crystal system	tetragonal	orthorhombic	monoclinic
Space group	$P4_2/n$	$P2_12_12_1$	$P2_1/n$
a (Å)	18.5981(1)	14.6085(2)	12.4556(2)
b (Å)	18.5981(1)	18.1164(2)	23.8012(3)
c (Å)	20.7899(1)	19.2324(3)	21.2193(2)
α (°)	90	90	90
β (°)	90	90	93.566(1)
γ (°)	90	90	90
V (Å^3)	7191.00(8)	5089.92(12)	6278.46(14)
Z	8	4	4
Radiation, λ (Å)	Cu Kα, 1.54184	Cu Kα, 1.54184	Cu Kα, 1.54184
Temp (K)	150(2)	150(2)	150(2)
ρ_{calc} (g cm^{-3})	1.168	1.618	1.601
μ (mm^{-1})	1.783	6.576	6.979
Reflections collected	77157	28937	76418
Indep. reflections	7522	9631	13093
Parameters	408	669	784
R(int)	0.0252	0.0262	0.0579
R1/wR2,[a] I ≥ 2σI (%)	3.01/8.21	2.30/5.69	3.54/8.19
R1/wR2,[a] all data (%)	3.46/8.69	2.46/5.84	4.74/8.85
GOF	1.038	1.051	1.076

[a] $R1 = [\Sigma||F_o| - |F_c||]/\Sigma|F_o|$; $wR2 = \{[\Sigma w[(F_o)^2 - (F_c)^2]^2]/[\Sigma w(F_o^2)^2]\}^{1/2}$; $w = [\sigma^2(F_o)^2 + (AP)^2 + BP]^{-1}$, where $P = [(F_o)^2 + 2(F_c)^2]/3$ and the A and B values are 0.0418 and 3.32 for **3**, 0.025 and 4.24 for **2b**·2tol and 0.0351 and 11.40 for **4c**·$2CH_2Cl_2$.

Table S6. Selected X-ray data collection/refinement parameters for **6a** and **6b**.

	**6a**	**6b**
Formula	$C_5H_9IN_3P$	$C_{11}H_{15}IN_3P$
CCDC	2263739	2263740
Fw [g mol^{-1}]	269.02	347.13
Crystal system	tetragonal	monoclinic
Space group	$P4_32_12$	$P2_1/c$
a (Å)	8.9844(1)	13.2814(4)
b (Å)	8.9844(1)	6.9637(2)
c (Å)	22.4971(6)	13.6715(3)
α (°)	90	90
β (°)	90	96.888(2)
γ (°)	90	90
V (Å^3)	1815.95(6)	1255.32(6)
Z	8	4
Radiation, λ (Å)	Cu Kα, 1.54184	Cu Kα, 1.54184
Temp (K)	100(2)	150(2)
ρ_{calc} (g cm^{-3})	1.968	1.837
μ (mm^{-1})	28.879	21.062
Reflections collected	20189	8550
Indep. reflections	1866	2600
Parameters	95	145
R(int)	0.1251	0.0317
R1/wR2,[a] I ≥ 2σI (%)	5.23/14.80	2.35/5.27
R1/wR2,[a] all data (%)	5.81/15.33	3.39/5.82
GOF	1.067	1.061

[a] $R1 = [\Sigma||F_o| - |F_c||]/\Sigma|F_o|$; $wR2 = \{[\Sigma w[(F_o)^2 - (F_c)^2]^2]/[\Sigma w(F_o^2)^2]\}^{1/2}$; $w = [\sigma^2(F_o)^2 + (AP)^2 + BP]^{-1}$, where $P = [(F_o)^2 + 2(F_c)^2]/3$ and the A and B values are 0.0981 and 0.18 for **6a**, and 0.025 and 4.24 for **6b.**

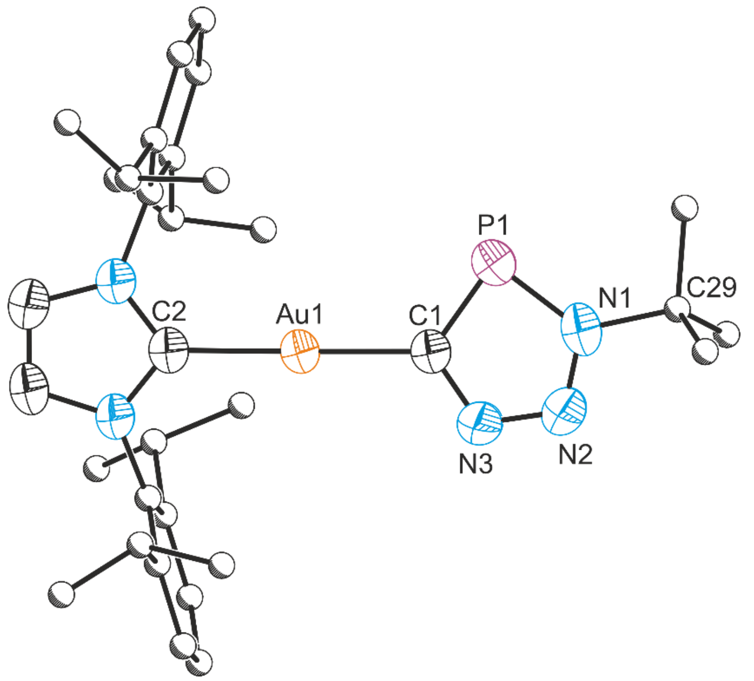

Figure S44. Solid-state structure of **1a**. Anisotropic displacement ellipsoids set at 50% probability. Hydrogen atoms and solvent of crystallization omitted for clarity. Carbon atoms of Dipp and ^{t}Bu substituents pictured as spheres of arbitrary radius. Selected bond lengths (Å) and angles (°): Au1–C1 2.022(8), Au1–C2 2.028(8), C1–P1 1.690(8), P1–N1 1.685(8), N1–N2 1.293(11), N2–N3 1.314(12), N3–C1 1.331(12), N1–C29 1.521(12); C1-Au1-C2 179.5(3), Au1-C1-P1 126.0(5), C1-P1-N1 88.4(4), P1-N1-N2 114.7(6), N1-N2-N3 111.0(8), N2-N3-C1, 115.7(8), N3-C1-P1, 110.2(6).

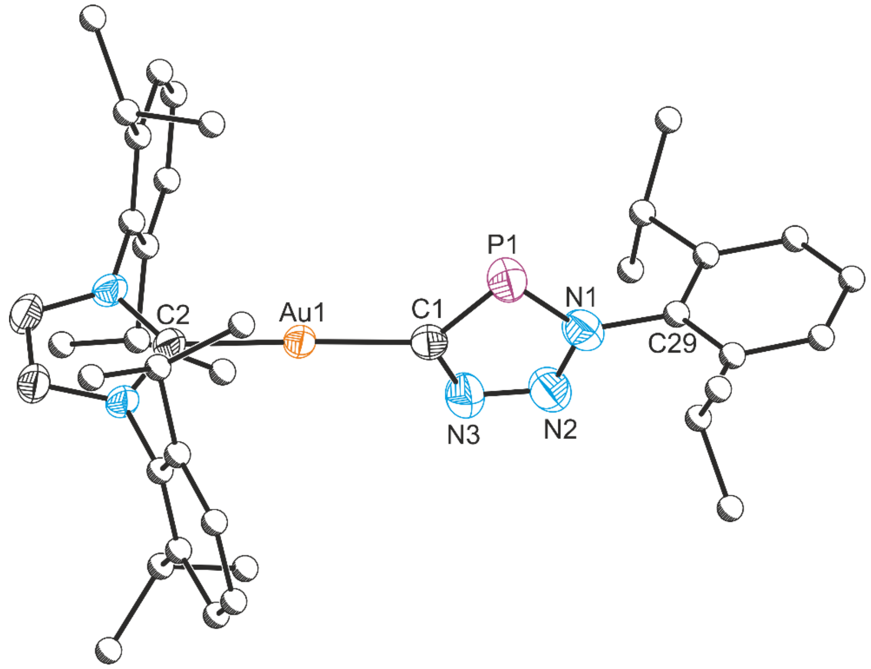

Figure S45. Solid-state structure of **1c**. Anisotropic displacement ellipsoids set at 50% probability. Hydrogen atoms and solvent of crystallization omitted for clarity. Carbon atoms of Dipp substituents pictured as spheres of arbitrary radius. Selected bond lengths (Å) and angles (°): Au1–C1 2.019(4), Au1–C2 2.015(4), C1–P1 1.719(4), P1–N1 1.704(4), N1–N2 1.346(6), N2–N3 1.311(6), N3–C1 1.346(6), N1–C29 1.455(6); C1-Au1-C2 177.25(16), Au1-C1-P1 126.5(2), C1-P1-N1 87.7(2), P1-N1-N2 114.3(3), N1-N2-N3 111.2(4), N2-N3-C1, 115.2(4), N3-C1-P1, 111.6(3).

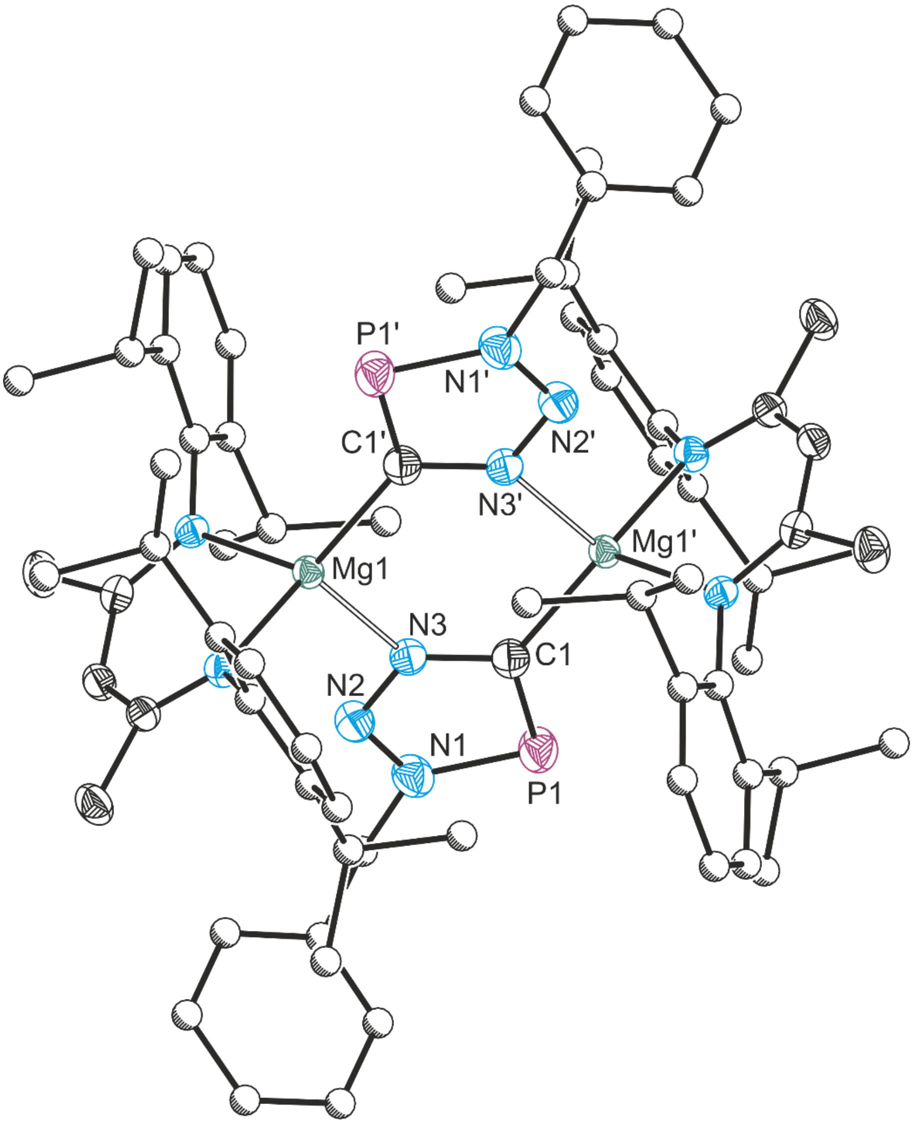

Figure S46. Bottom: Solid-state structure of **2b**. Anisotropic displacement ellipsoids depicted at 50% probability. Hydrogen atoms and solvent of crystallization omitted for clarity. Carbon atoms of Dipp and Bn substituents pictured as spheres of arbitrary radius. Selected bond lengths (Å) and angles (°): Mg1–C1′ 2.164(3), C1–P1 1.725(3), P1–N1 1.707(2), N1–N2 1.312(3), N2–N3 1.319(3), N3–C1 1.377(3), N3–Mg1 2.109(2); Mg1-C1-P1 131.84(15), C1-P1-N1 88.89(12), P1-N1-N2 115.32(18), N1-N2-N3 110.5(2), N2-N3-C1, 117.0(2), N3-C1-P1, 108.31(18), N2-N3-Mg1 107.72(16). Symmetry operation ′ : 1–*x*, 1–*y*, 1–*z*.

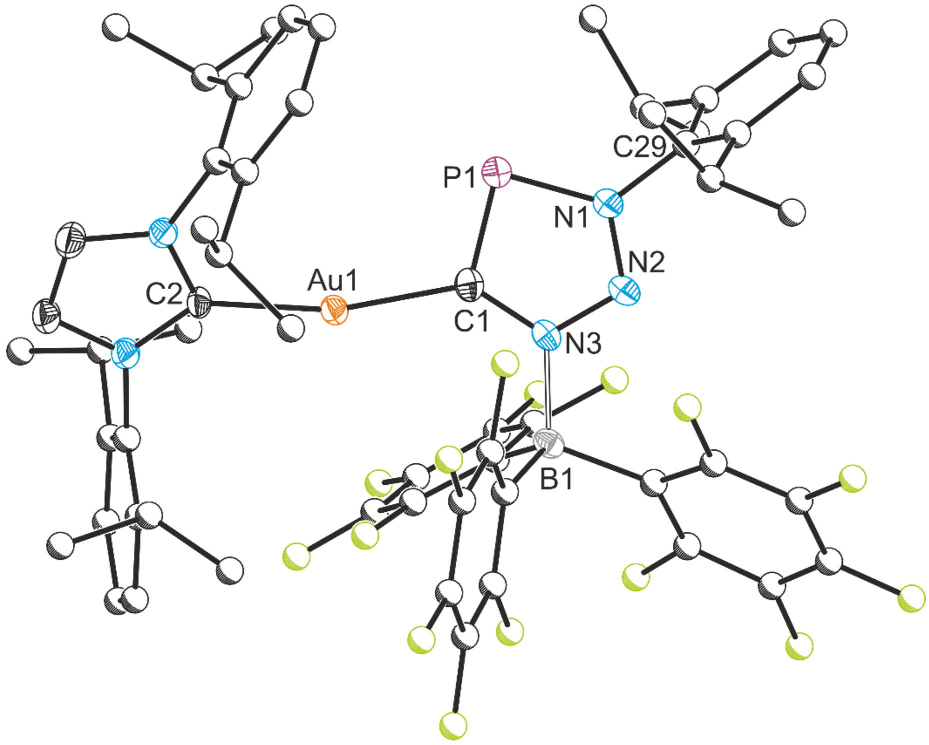

Figure S47. Bottom: Solid-state structure of **4c**. Anisotropic displacement ellipsoids depicted at 50% probability. Hydrogen atoms and solvent of crystallization omitted for clarity. Carbon atoms of Dipp and C_6F_5 substituents pictured as spheres of arbitrary radius. Selected bond lengths (Å) and angles (°): Au1–C1 2.028(3), Au1–C2 2.019(3), C1–P1 1.718(4), P1–N1 1.714(3), N1–N2 1.314(4), N2–N3 1.328(4), N3–C1 1.364(5), N1–C29 1.449(5), N3–B1 1.604(5); C1-Au1-C2 165.09(14), Au1-C1-P1 113.93(19), C1-P1-N1 87.64(16), P1-N1-N2 116.5(2), N1-N2-N3 109.1(3), N2-N3-C1 116.9(3), N3-C1-P1 109.8(3), N2-N3-B1 118.3(3).

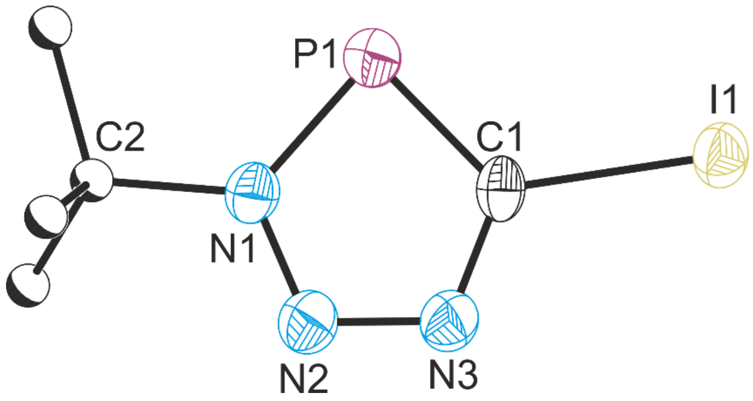

Figure S48. Solid-state structure of **6a**. Anisotropic displacement ellipsoids depicted at 50% probability. Hydrogen atoms omitted for clarity. Carbon atoms of ^{t}Bu substituent pictured as spheres of arbitrary radius. Selected bond lengths (Å) and angles (°): I1–C1 2.083(6), C1–P1 1.727(7), P1–N1 1.706(6), N1–N2 1.329(8), N2–N3 1.321(8), N3–C1 1.342(9), N1–C2 1.506(9); I1-C1-P1 125.3(4), C1-P1-N1 85.3(3), P1-N1-N2 115.8(5), N1-N2-N3 112.4(6), N2-N3-C1 111.8(6), N3-C1-P1 114.8(5).

3. Computational details

3.1. General computational methods

Density functional theory (DFT) calculations were performed using the ORCA 5.0.2 software package.[40–42] All methods were used as implemented. Geometries were optimized using the B97-D3 functional using the SARC-ZORA-TZVP all-electron relativistically contracted basis set for iodine and the ZORA-def2-SVP basis set for all other atoms, correcting for relativistic effects using the zeroth order regular approximation (ZORA).[43,44] Analytical frequency calculations were carried out to verify all geometries as true minima (N_{imag} = 0). Single point calculations were performed using the ωB97X-D3 functional[45] and the Resolution of Identity approximation (RIJCOSX),[46,47] and corrected for relativistic effects using the zeroth order regular approximation (ZORA). The segmented all-electron relativistically contracted basis set SARC-ZORA-TZVPP was used for I, and the relativistically contracted triple-zeta basis set ZORA-def2-TZVPP was used for all other atoms, along with the SARC/J auxiliary basis set.[46,47] Electrostatic potential surfaces were calculated using Multiwfn 3.8.[48]

3.2. $ICPN_3R$

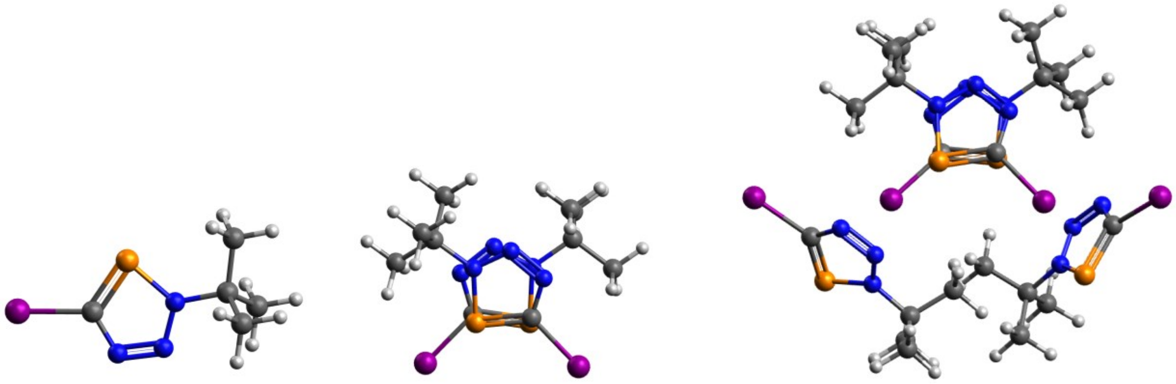

ΔG_{rel} = 0 kcal mol^{-1} ΔG_{rel} = −22.2 kcal mol^{-1} ΔG_{rel} = −29.8 kcal mol^{-1}

Figure S49. Relative energies of $ICPN_3{}^tBu$ aggregates in the gas phase.

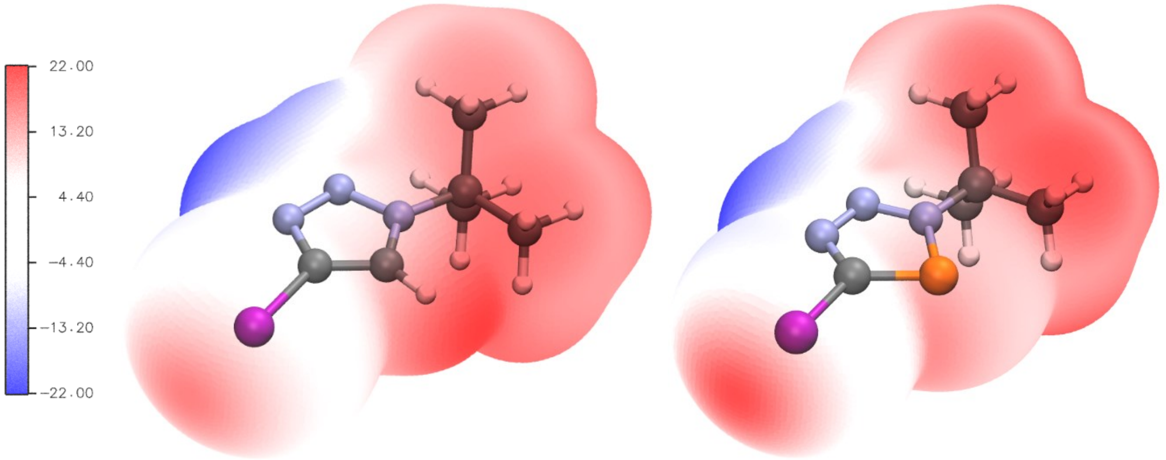

I σ-hole: +20.2 kcal mol^{-1} I σ-hole: +20.7 kcal mol^{-1}

Figure S50. Electrostatic potential plotted on the molecular Van der Waals surface for $ICCHN_3{}^tBu$ (left) and $ICPN_3{}^tBu$ (right).

3.5. XYZ coordinates

ICPN$_3$^{t}Bu

I	0.0142851176	-0.0117279802	2.1257502713
C	0.0057234191	-0.0335879140	0.0235382100
P	-0.0287438728	1.3872308643	-1.0028831404
N	0.0268568321	-1.2087170883	-0.6366960447
N	0.0178076221	-1.0606439048	-1.9306381349
N	-0.0101598158	0.2189931358	-2.3040075059
C	1.2514779643	-0.1299145896	-4.3850579489
H	1.2715528030	0.0481749690	-5.4698706787
H	2.1468539165	0.3270139836	-3.9387704394
H	1.2823975550	-1.2111998263	-4.2006874816
C	-0.0249473637	0.4843347223	-3.7805064209
C	-0.0500825247	2.0012743041	-4.0141491271
H	-0.0590438800	2.1962416890	-5.0959025912
H	-0.9488529569	2.4632742498	-3.5796881529
H	0.8387121746	2.4906433101	-3.5893099213
C	-1.2904360911	-0.1699427634	-4.3657841880
H	-1.3330269647	0.0068688949	-5.4501648912
H	-1.2836814628	-1.2515911082	-4.1808822152
H	-2.1928924720	0.2584750521	-3.9056895990

ICCHN$_3$^{t}Bu

I	0.0078168659	0.4837049775	1.9294553556
C	0.0036327784	0.0544996010	-0.1152717074
C	-0.0206046184	0.9549384318	-1.1769707664
N	0.0242676145	-1.2132097137	-0.6044482380
N	0.0160087817	-1.1517766182	-1.9111688478
N	-0.0102487559	0.1489336058	-2.2760010195
C	1.2499156609	-0.0953187666	-4.3614724976
H	1.2645310993	0.1218184526	-5.4392110986
H	2.1475875807	0.3449459005	-3.9032591358
H	1.2827271947	-1.1824207532	-4.2159434340
C	-0.0254027553	0.4922344687	-3.7285767279
C	-0.0509657325	2.0185839402	-3.8870648362
H	-0.0605752355	2.2627063519	-4.9583223780
H	-0.9514112765	2.4596933596	-3.4351959737
H	0.8396631575	2.4883035508	-3.4446688133
C	-1.2894683286	-0.1364997220	-4.3442788848
H	-1.3264523510	0.0803282081	-5.4215513624
H	-1.2842989577	-1.2241369605	-4.1990823780
H	-2.1946321537	0.2739185709	-3.8732663889
H	-0.0380405687	2.0370631148	-1.2079608673

4. References

[33] P. Margaretha, S. Solar, O. E. Polansky, *Angew. Chem. Int. Ed.* **1971**, *10*, 412–413.

[34] K. Barral, A. D. Moorhouse, J. E. Moses, *Org. Lett.* **2007**, *9*, 1809–1811.

[35] H. Gallardo, A. J. Bortoluzzi, D. M. P. De Oliveira Santos, *Liq. Cryst.* **2008**, *35*, 719–725.

[36] Y. Ding, H. W. Roesky, M. Noltemeyer, H. G. Schmidt, P. P. Power, *Organometallics* **2001**, *20*, 1190–1194.

[37] R. Evans, Z. Deng, A. K. Rogerson, A. S. McLachlan, J. J. Richards, M. Nilsson, G. A. Morris, *Angew. Chem. Int. Ed.* **2013**, *52*, 3199–3202.

[38] *CrysAlisPro*, Agilent Technologies, Version 1.171.41.117a.

[39] (a) G. M. Sheldrick in *SHELXL97, Programs for Crystal Structure Analysis* (Release 97-2), Institut für Anorganische Chemie der Universität, Tammanstrasse 4, D-3400 Göttingen, Germany, 1998; (b) G. M. Sheldrick, *Acta Crystallogr. Sect. A* **1990**, *46*, 467−473; (c) G. M. Sheldrick, A*cta Crystallogr. Sect. A* **2008**, *64*, 112−122.

[40] F. Neese, *Wiley Interdiscip. Rev. Comput. Mol. Sci.* **2012**, *2*, 73–78.

[41] F. Neese, *Wiley Interdiscip. Rev. Comput. Mol. Sci.* **2018**, *8*, 1–6.

[42] F. Neese, F. Wennmohs, U. Becker, C. Riplinger, *J. Chem. Phys.* **2020**, *152*, 224108.

[43] S. Grimme, *J. Comput. Chem.* **2006**, *27*, 1787–1799.

[44] F. Weigend, R. Ahlrichs, *Phys. Chem. Chem. Phys.* **2005**, *7*, 3297–3305.

[45] Y. S. Lin, G. De Li, S. P. Mao, J. Da Chai, *J. Chem. Theory Comput.* **2013**, *9*, 263–272.

[46] D. A. Pantazis, X. Y. Chen, C. R. Landis, F. Neese, *J. Chem. Theory Comput.* **2008**, *4*, 908–919.

[47] M. Bühl, C. Reimann, D. A. Pantazis, T. Bredow, F. Neese, *J. Chem. Theory Comput.* **2008**, *4*, 1449–1459.

[48] T. Lu, F. Chen, *J. Comput. Chem.* **2012**, *33*, 580–592.
